# Identification and Comparison of Candidate Olfactory Genes in the Olfactory and Non-Olfactory Organs of Elm Pest *Ambrostoma quadriimpressum* (Coleoptera: Chrysomelidae) Based on Transcriptome Analysis

**DOI:** 10.1371/journal.pone.0147144

**Published:** 2016-01-22

**Authors:** Yinliang Wang, Qi Chen, Hanbo Zhao, Bingzhong Ren

**Affiliations:** 1 Jilin Provincial Key Laboratory of Animal Resource Conservation and Utilization, Northeast Normal University, Changchun, China; 2 Key Laboratory of Vegetation Ecology, MOE, Northeast Normal University, Changchun, China; USDA-ARS, UNITED STATES

## Abstract

The leaf beetle *Ambrostoma quadriimpressum* (Coleoptera: Chrysomelidae) is a predominant forest pest that causes substantial damage to the lumber industry and city management. However, no effective and environmentally friendly chemical method has been discovered to control this pest. Until recently, the molecular basis of the olfactory system in *A*. *quadriimpressum* was completely unknown. In this study, antennae and leg transcriptomes were analyzed and compared using deep sequencing data to identify the olfactory genes in *A*. *quadriimpressum*. Moreover, the expression profiles of both male and female candidate olfactory genes were analyzed and validated by bioinformatics, motif analysis, homology analysis, semi-quantitative RT-PCR and RT-qPCR experiments in antennal and non-olfactory organs to explore the candidate olfactory genes that might play key roles in the life cycle of *A*. *quadriimpressum*. As a result, approximately 102.9 million and 97.3 million clean reads were obtained from the libraries created from the antennas and legs, respectively. Annotation led to 34344 Unigenes, which were matched to known proteins. Annotation data revealed that the number of genes in antenna with binding functions and receptor activity was greater than that of legs. Furthermore, many pathway genes were differentially expressed in the two organs. Sixteen candidate odorant binding proteins (OBPs), 10 chemosensory proteins (CSPs), 34 odorant receptors (ORs), 20 inotropic receptors [1] and 2 sensory neuron membrane proteins (SNMPs) and their isoforms were identified. Additionally, 15 OBPs, 9 CSPs, 18 ORs, 6 IRs and 2 SNMPs were predicted to be complete ORFs. Using RT-PCR, RT-qPCR and homology analysis, AquaOBP1/2/4/7/C1/C6, AquaCSP3/9, AquaOR8/9/10/14/15/18/20/26/29/33, AquaIR8a/13/25a showed olfactory-specific expression, indicating that these genes might play a key role in olfaction-related behaviors in *A*. *quadriimpressum* such as foraging and seeking. AquaOBP4/C5, AquaOBP4/C5, AquaCSP7/9/10, AquaOR17/24/32 and AquaIR4 were highly expressed in the antenna of males, suggesting that these genes were related to sex-specific behaviors, and expression trends that were male specific were observed for most candidate olfactory genes, which supported the existence of a female-produced sex pheromone in *A*. *quadriimpressum*. All of these results could provide valuable information and guidance for future functional studies on these genes and provide better molecular knowledge regarding the olfactory system in *A*. *quadriimpressum*.

## Introduction

Insects evolved a highly sensitive and acute peripheral system that can selectively detect environmental molecules. These molecules contain information that regulates a series of important insect behaviors, such as mating [2], foraging [3], oviposition [4], and host-seeking [5]. Several types of olfactory proteins play key roles in determining or helping to complete the selective detection process for the odorants, including odorant-binding proteins, odorant receptors, ionotropic receptors and sensory neuron membrane proteins and odorant-degrading enzymes [6]. Through the great efforts of many scholars during the past decade, a convincing model for the peripheral detection of odorants in insects was established, the function, structure and mechanism of many OBPs and ORs have been studied through biochemical, molecular biological and electrophysiological experiments [4, 7–9]. Recently, the identification of a new family of IRs, which are likewise stimulated by odorants but expressed in different olfactory neurons, have provided new insights into the molecular mechanisms of odorant reception in insects [1].

Although the knowledge of the molecular olfactory system has grown rapidly since the first insect ORs were identified [10], our current knowledge of the olfactory system in insects is still highly reliant on a few insects, such as *D*. *melanogaster* and *Anopheles gambiae*. Additionally, many phenomena cannot be explained because of insect diversity; for example, the behaviors and habitats between Dipteran species and other insects may exhibit differences not only in the type of odorants used but also in the reception process of these semiochemicals. Therefore, the current knowledge of insect olfactory genes remains limited. To the best of our knowledge, olfactory genes have been systematically analyzed in a limited number of coleopteran species, including *Tribolium castaneum*, *Dendroctonus ponderosaein*, *Ips typographus* (genome sequences are available), *Monochamus alternatus*, *Dastarcus helophoroides*, *Rhizopertha dominica*, *Leptinotarsa decemlineata*, *Tenebrio molitor*, *Megacyllene caryae*, *Anomala corpulenta* and *Dendroctonus valens* (based on RNA sequencing data) [11–20]. With the rapid growth of sequencing and bioinformatics, there has been a marked increase in the number of insect olfactory genes identified based on genome or transcriptome analysis. Despite these advances, our molecular understanding of olfaction for a large number of coleopteran species that have substantial influences on agriculture, forestry and human health remains completely unknown.

The beetle *Ambrostoma quadriimpressum* (Coleoptera: Chrysomelidae) is a major forest pest. It is a monophagous species found in the East Asian region, especially in Northeast China, where it feeds exclusively on the shoots and leaves of elms, such as *Ulmus pumila*, *Ulmus macrocarpa* and *Ulmus japonica*. This forest pest lives on the elm for nearly 6 months every year from April to October. After October, they crawl into the soil for winter, but they emerge back onto the elm for oviposition in April of the next year. Both larval and adult *A*. *quadriimpressum* feed on elm leaves. When an outbreak occurs, nearly all of the elm leaves are eaten, resulting in an irregular death before the elm is fully grown. These pests seriously damage wooded forests and city green belts and have caused billions of dollars in property loss in the lumber industry and city management [21].

Previous behavioral research has demonstrated that male *A*. *quadriimpressum* are attracted by a hexane extract from the female elytra and attempt to mate with artificial females bathed in this extract, highly supporting the existence of a female-produced contact sex pheromone in *A*. *quadriimpressum* [22]. Furthermore, GC-MS analysis indicated that the extracts contained mainly straight-chain hydrocarbon compounds with mono-, di-, and trimethyl branches, with four compounds that were unique to females and eight hydrocarbons present at significantly higher levels than in males, suggesting a candidate contact sex pheromone role for these chemicals [22]. Moreover, because a series of elm volatiles and herbivore induced plant volatiles (HIPVs) might elicit significant EAG and attract/repel responses in *A*. *quadriimpressum* [23], these results indicated that olfaction might play crucial roles in mating, foraging, and host-seeking behaviors in *A*. *quadriimpressum*; however, the molecular basis for the olfactory system in *A*. *quadriimpressum* remains completely unknown.

In the past, the prevention and control methods of this pest consisted of using highly toxic chemicals, such as phosphamidon, carbofuran and omethoate [24]. These chemicals not only seriously pollute the environment but are also have hazardous off-target effects in humans, bees and cattle. A broader understanding of olfaction in *A*. *quadriimpressum* is the first step in identifying an effective and safe control method for this pest. Thus, systematic identification of odorant genes in *A*. *quadriimpressum* is urgently needed to facilitate future functional studies on odorant genes and will help elucidate the mechanism of olfactory-related behaviors in *A*. *quadriimpressum*, allowing the exploration of new chemical control methods.

In this study, antennae and leg transcriptomes were analyzed and compared based on deep sequencing data to identify the existence and sequences of OBPs, CSPs, ORs, IRs and SNMP genes in *A*. *quadriimpressum* for future functional studies. Moreover, the expression profiles of both male and female candidate olfactory genes were analyzed and validated by bioinformatics, semi-quantitative RT-PCR and RT-qPCR experiments to explore the candidate olfactory genes that may play a key role in the life cycle of *A*. *quadriimpressum*.

## Material and Methods

### Insects

Newly emerged adult beetles (*A*. *quadriimpressum*) were collected from elm (*Ulmus pumila*) trees in June in Jilin province (E125.3 N43.9, E124.2 N 44.5), China, and maintained in an artificial climate box (Boxun, Shanghai, China) kept at 28°C and 85% humidity, where they were fed fresh elm leaves under a 12:12-h light–dark cycle. The total number of adults was more than three hundred, and the male/female ratio was approximately 1:1. The sample collection was authorized by the forestry bureau of Jilin province.

### RNA Extraction, cDNA Synthesis and Sequencing

Antennae, hindlegs, underwing and thorax muscles were carefully separated from the insect (to avoid age differences, total RNA was extracted from the adults, which were collected at the same location and date) by DEPC-treated forceps under a stereomicroscope (Motic, Hong Kong, China). These appendages were collected from 60 mixed samples (male/female ratio 1:1 for RNA sequencing) including 30 females and 30 males. Thirty paired samples of hindleg RNA (for semi-quantitative RT-PCR and RT-qPCR) was also collected from from thirty paired hindlegs as a non-olfactory tissue control, and the collected tissue was stored on ice with DEPC-treated 1:1 water/ethanol solutions before being used. Total RNA was isolated from homogenized antennas in Trizol reagent (Invitrogen, Carlsbad, CA, USA) following the manufacturer’s protocols, followed by a ethanol/isopropanol precipitation with a final solution of 30 μl RNase-free water. After extraction, the total RNA sample was assessed with a NanoDrop 2000 (Thermo Fisher Scientific, Waltham, MA, USA) and in a 1% agarose gel. UV absorption values at 230 nm/260 nm and 260 nm/280 nm were recorded to monitor the purity of the RNA products, and the mRNA smear above the 28S rRNA band was also checked to verify RNA integrity. For semi-quantitative RT-PCR and RT-qPCR experiments, cDNA samples were quantified by total RNA before RT, with a final concentration of 176 ng/μl (according to the lowest concentration of all the samples; other samples were all diluted to this concentration for cDNA quantification) in all of the mixed, male, and female antenna and leg samples. Then, the same amount of total RNA from each sample was transcribed into cDNA in a reaction (total volume of 20 μl) containing 3 μl (528 ng) total RNA solution, 4 μl first-strand buffer (250 mM Tris pH 8.3, 375 mM KCl, and 15 mM MgCl_2_), 1 μl 10-mM dNTP mix, 1 μl RNaseout, 1 μl DTT (0.1 M), 1 μl oligo-(dT)20 primer (50 μM) and 1 μl Superscript III reverse transcriptase (200 units/μl) (Invitrogen, Carlsbad, CA, USA). The cDNA synthesis was performed for 45 min at 50°C, followed by 15 min at 70°C. Finally, quantified sex- and organ-specific samples were diluted 1:50 prior to use as PCR templates for quantitative analysis. For RNA sequencing, total RNA samples from the antenna and legs were evaluated on an Agilent 2010 Bioanalyzer (Agilent Technologies, Santa Clara, CA, USA). High-quality RNA was sent to Biomarker Technologies Corporation (Beijing, China) for cDNA library construction and sequencing. mRNA was purified using the interaction of the poly (A) tails and magnetic oligo (dT) beads. RNA sequencing libraries were generated using the NEBNext^®^ Ultra RNA Library Prep Kit for Illumina sequencing (New England Biolabs, Ipswich, MA, USA) with multiplex primers according to the manufacturer’s protocol. The cDNA library was constructed with average inserts of 200 bp (150–250 bp) using a non-stranded library preparation. The antenna and leg cDNA samples were bar-coded (antenna: CTTGTA; legs: AGTCAA) and purified using AMPure XP Beads (Beckman Coulter, Inc.). The short cDNA fragments were cloned using end-repair adapter ligation. Then, suitable fragments were selected with Agencourt AMPure XP beads (Beckman Coulter, Inc.) and enriched by PCR amplification. Sequencing was performed via a paired-end 125-cycle rapid run on an Illumina HiSeq2000.

### Assembly and Functional Annotation

High-quality clean reads were obtained by removing the adaptor sequences, duplicated sequences, ambiguous reads (‘N’), and low-quality reads. Transcriptomes were separately assembled de novo using Trinity (http://trinityrnaseq.sourceforge.net/) as previously described [13]. In brief, clean reads with a certain overlap length were initially combined to form long fragments without N, termed contigs. Related contigs were clustered using the TGICL software to yield unigenes (without N) that could not be extended on either end, and redundancies were removed to acquire non-redundant unigenes. Sense and antisense strand ORFs were predicted by Getorf (http://emboss.sourceforge.net/apps/cvs/emboss/apps/getorf.html). Unigenes were first annotated with the COG, GO, and KEGG databases. The Swissprot and NR databases were used for further exploration of the candidate olfactory genes of *Ambrostoma quadriimpressum*. The olfactory genes of *Tribolium castaneum*, *Ips typographus* and *Dendroctonus ponderosae* were also blasted with a local Unigene database. After identification, candidate olfactory genes were classified according to their length, ORF, start and stop codons, conserved Cys locations, signal peptides and transmembrane domains. For OBPs and CSPs, the obtained ORF sequences were first aligned by MUSCLE [25]; additionally, the number and location of Cys amino acids from each candidate gene were manually checked and aligned with their “best hit” OBPs from other coleopteran species, Signal peptides were predicted using SignalP 4.1 server (www.cbs.dtu.dk/services/SignalP). For ORs and IRs, transmembrane regions were predicted by the TMHMM2.0 online tool (http://www.cbs.dtu.dk/services/TMHMM/). Further alignments of SNMPs and their isoforms in *A*. *quadriimpressum* and *Tribolium castaneum* were performed by ESPript (http://espript.ibcp.fr/ESPript/cgi-bin/ESPript.cgi). The expression level of each identified gene from the antenna and leg was predicted with the FPKM method (Fragments per Kb per million reads) [26], and the FKPM values were analyzed and output as TIF files with Graphpad Prism 5.0 (GraphPad Software, La Jolla, CA, USA). The nucleotide sequences of each identified olfactory gene are listed in S1 Table.

### Motif analysis

A total of 197 OBPs and 86 CSPs from *A*. *quadriimpressum*, *T*. *castaneum*, *D*. *ponderosaein*, *I*. *typographus*, *M*. *alternatus*, *L*. *decemlineata*, *T*. *molitor*, *A*. *corpulenta* and *D*. *valens* were used for motif and pattern analysis among Coleoptera. The sequence integrity of the chosen OBPs and CSPs were confirmed manually by ORF length and the location of conserved Cys amino acids. The MEME 4.10.2 and MAST online software (http://meme.nbcr.net/meme/) were used to discover and analyze the motifs [27]. The parameter settings were as follows: minimum width = 6, maximum width = 10, and maximum number of motifs to find = 8. After that, each identified motif was further evaluated against the Ensembl Genome and Swiss Protein databases to find the best match by MAST. It should be noted that the PBPs family was not used in this analysis because the putative PBPs recovered from Coleoptera were mostly partial sequences.

### Homology Analysis

A neighbor-joining (NJ) tree was constructed with MEGA version 6 [28] and the Jones-Taylor-Thornton model. The olfactory gene sequences of other insects were first transcribed into their amino acid sequences using the ORF finder (http://www.ncbi.nlm.nih.gov/gorf/gorf.html and aligned by MUSLE. Olfactory genes of other coleopteran species were obtained from the NCBI and EMBL-EBI (http://www.ebi.ac.uk/services) databases. Bootstrap support values were based on 1000 replicates. All of the candidate olfactory genes were named according to the nomenclature system described previously [29]. The olfactory genes from different species were marked with different colors and generated with Figtree 1.42 software (http://tree.bio.ed.ac.uk/software/figtree/).

### RT-PCR and RT-qPCR Validation

Fifteen OBPs, 9 CSPs, 17 ORs, 9 IRs and 2 SNMPs that were predicted to be highly abundant in antenna or legs or had complete ORFs were selected for further analysis. The cDNA samples for semi-quantitative reverse-transcription PCR and quantitative reverse-transcription PCR were prepared as described above. For the expression profile and semi-quantitative RT-PCR experiments, 1-μl quantified cDNA samples from the antenna and legs of both males and females were used as the template. ß-Actin was used as an internal control. The PCR conditions were as follows: 1 min and 30 s at 94°C and then 30 cycles at 94°C for 30 s, 45°C for 30 s, 68°C for 30 s and 68°C for 7 min, with a final volume of 25 μl. After the PCR reactions were finished, 8 μl aliquots of each PCR reaction was analyzed on 1% agarose gels. RT-qPCR (qPCR) was performed using the StepOne Plus Real-Time PCR Detection System (Bio-Rad, Hercules, CA, USA) and TransStar Tip Top Green qPCR Supermix (Transgen Biotech, Beijing, China). The ß-actin gene was used as an internal control: ß-actin forward and reverse, 5’–AACGATACCGTGTTCAATGG-3’ and 5’–CATCGTCGGTCGTCCAAG-3’. PCR reagents were used following the manufacturer’s instructions, and the PCR conditions were as follows: 94°C for 30 s, followed by 40 cycles of 94°C for 5 s, 60°C for 15 s, and 72°C for 34 s. RT-qPCR data analysis was performed using the 2^-ΔΔCT^ method. All of the primers used in this experiment were designed with Array Designer 4.3 (PRIMIER Biosoft, Palo Alto, CA, USA) and are listed in S2 Table. The qPCR data were analyzed and output as TIF files using Graphpad Prism 5.0. For RT-PCR, the intensity of target bands were analyzed and compared using ImageJ 1.6 (http://rsb.info.nih.gov/ij/).

Finally, an expression level heatmap was created with Hem I [30] to visually compare all of the candidate olfactory genes based on the following set: 1. Log_2_ (Antenna FKPM/ Leg FKPM); 2. Log_2_ (Antenna relative fold change/Non-olfactory organ relative fold change (average values of wing, leg and thorax)); 3. Log_2_ (Intensity of target bands from antenna/Intensity of target bands from the non-olfactory organ). The absolute value of log2 ratio ≥ 0 was set as the threshold for determining the difference in gene expression difference between the two organs. After the expression data were obtained, they were normalized to ±1 by their range, and an expression heatmap was generated. Value> 0 indicate that the gene shows olfactory specificity, whereas when the value < 0, it indicates that the gene does not show olfactory specificity. Considering the differences between the three methods (RNA-seq, RT-PCR and RT-qPCR), we only drew conclusions when the results of all three experiments were consistent.

## Results

### Transcriptome Overview

After the raw data were filtered using the strategy described above, approximately 102.9 million and 97.3 million clean reads comprising 25.93 and 24.51 gigabases were obtained from the antenna and leg libraries respectively. Assemblies led to the generation of 68,737 bar-coded unigenes. The dataset was 55.13 megabases in size, with a mean length of 805 nt and an N50 of 1,609 nt. Therefore, 21.12% of the Unigenes exceeded 1,000 nt in length. Through annotation by BLASTx using the GO, Swissprot, COG, KEGG and NR databases, 34344 unigenes were matched to known proteins (Table 1). All of the SRA data used in this study were uploaded to NCBI with accession numbers SRS876374 and SRS876375.

**Table 1:** Overview of transcriptome data from the antenna and leg of *A*. *quadriimpressum*.

**Raw Data**			**Contig**			**Transcripts**		
	**Antenna**	**Leg**	**Contigs Length**	**Total Number**	**Percentage**	**Transcripts Length**	**Total Number**	**Percentage**
**ReadSum**	102967703	97331200	0–300	1768015	0.9722	200–300	32669	0.2315
**BaseSum**	25934264413	24518009356	300–500	19682	0.0108	300–500	24451	0.1733
**GC(%)**	41.22	43.05	500–1000	14181	0.0078	500–1000	25375	0.1798
**N(%)**	0.14	0.14	1000–2000	9404	0.0052	1000–2000	26314	0.1865
**Q20%**	94.61	94.56	2000+	7260	0.004	2000+	32288	0.2288
**CycleQ20%**	100	100	Total Number	1818543		Total Number	141097	
**Q30%**	90.86	90.67	Total Length	143898458		Total Length	196851121	
			N50 Length	145		N50 Length	2712	
			Mean Length	79.12843304		Mean Length	1395.147459	
**Unigene**			**Functional annotation of Unigene**					
**Unigenes Length**	**Total Number**	**Percentage**	**Anno_Database**		**Annotated_Number**	**300< = length<1000**		**length> = 1000**
200–300	26683	0.3882	COG_Annotation		9782	3412		4787
300–500	16116	0.2345	GO_Annotation		19209	7432		7074
500–1000	11421	0.1662	KEGG_Annotation		9428	3501		3951
1000–2000	7841	0.1141	Swissprot_Annotation		20096	7387		9055
2000+	6676	0.0971	nr_Annotation		34104	13737		12171
Total Number	68737		All_Annotated		34344	13816		12176
Total Length	55233166							
N50 Length	1609							
Mean Length	803.5434482							

### Overall Comparison of Transcription in Olfactory and Non-olfactory Tissues

**Fig 1:**
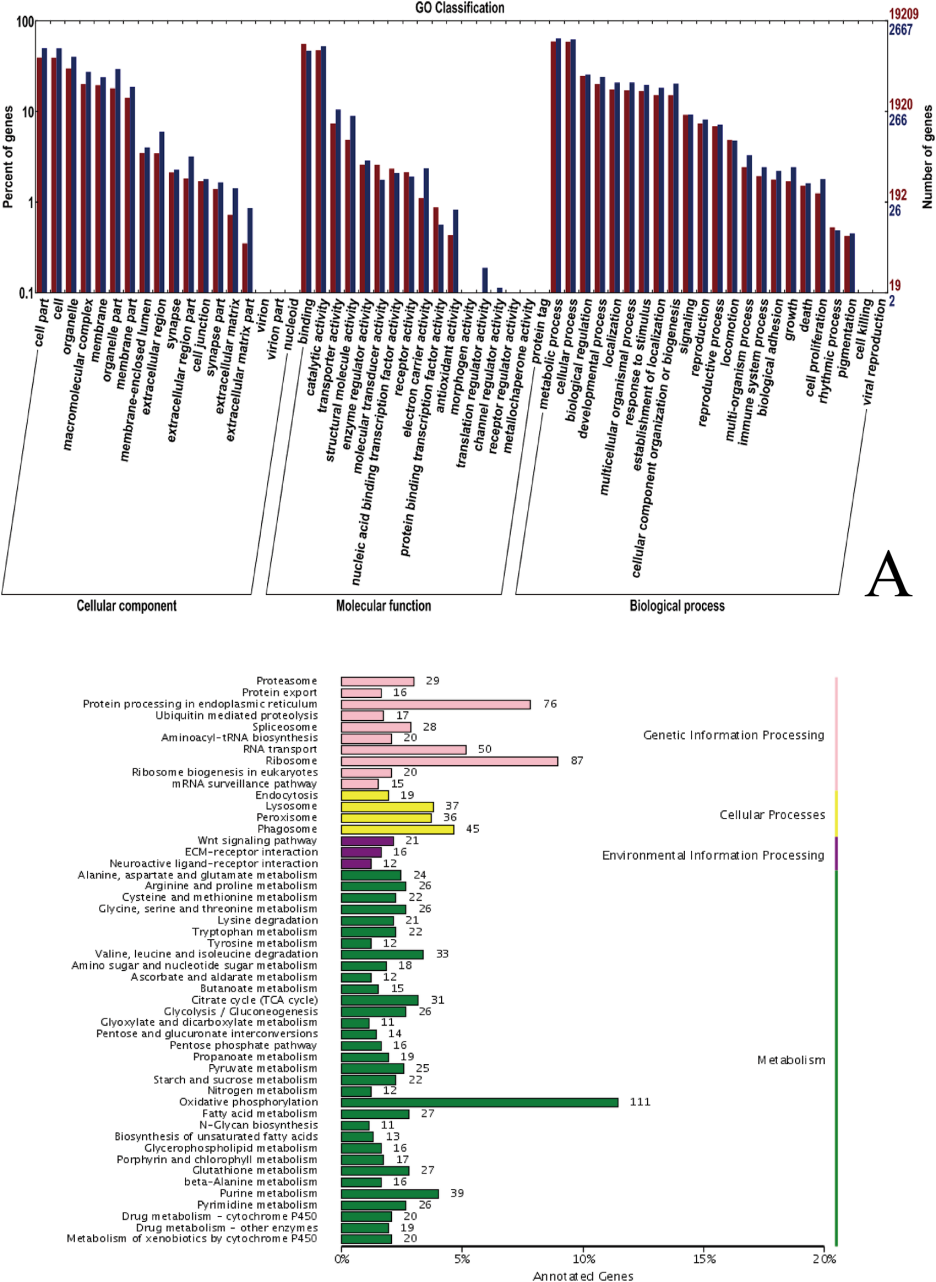
Comparison of functional annotations in olfactory and non-olfactory tissue derived transcripts. A. Comparison of GO annotation functional genes. Red indicates leg, while blue indicates antenna; B. KEGG pathway annotations. The bar represents the number of antenna/leg gene ratios for relevant pathways.

GO annotation showed that there was no large difference between the genes with different functions. In most cases, the gene numbers in the leg were only slightly higher than those in the antenna, except for genes with “binding”, “molecular transducer”, “nucleic acid binding transcription factor” and “receptor” activities. This finding is not surprising because the reception, binding and signal transduction roles of the insect antenna have been suggested at the transcriptional level (Fig 1A). KEGG pathway annotation showed that differentially expressed genes exist in many pathways. For antenna, the expression levels of genes relevant to “Oxidative phosphorylation (111/321)”, “Glycine, serine and threonine metabolism (26/53)”, “Valine, leucine and isoleucine degradation (33/90)”, “Citrate cycle (31/84)” and “Tryptophan metabolism (22/61)” pathways were much higher than those of in legs (Fig 1B), which suggested that the olfactory reception process in the antenna of *A*. *quadriimpressum* is highly reliant on oxidative phosphorylation for energy and amino acid metabolic pathways. Detailed information on these differentially expressed genes will require further studies.

### Identification of Candidate Olfactory Genes

The unigenes related to candidate chemosensory receptors were identified by a keyword search of the BLASTx annotation. To further explore the olfactory genes, a local blast was performed on the *A*. *quadriimpressum* unigene database based on the known olfactory sequences of *Tribolium castaneum*, *Ips typographus* and *Dendroctonus ponderosae*, and 16 OBPs, 10 CSPs, 34 ORs, 20 IRs and 2 SNMPs and their isoforms were identified. As such, 15 OBPs, 9 CSPs, 18 ORs, 6 IRs and 2 SNMPs were predicted to be complete ORFs by the following standards: 1. For OBPs and CSPs, those with a complete ORF length greater than 100 aa with locally conserved Cys amino acids. 2. Complete ORF of ORs and IRs were identified by their length, number and the location of their transmembrane regions. 3. SNMPs were aligned and compared with other coleopteran species to confirm their importance. The detailed information for each identified olfactory gene is listed in Table 2 according to their category. The calculated expression values based on the FKPM method of all olfactory genes are also listed (Table 2).

**Table 2:** Detailed information on the candidate olfactory genes of *A*. *quadriimpressum*.

Gene name	Unigene ID	ORF (aa)	Status	FPKM (Antenna /Leg)	BLASTx best hit	Gene ID	Signal Peptide	TMD
**AquaOBP 1**	c18871.graph_c0	126	Complete ORF	326.34/3.01	odorant binding protein 05 [Tribolium castaneum]	EFA05677.1		
**AquaOBP C1**	c28544.graph_c0	128	Complete ORF	87.41/1.59	odorant binding protein C13 [Tribolium castaneum]	EEZ97789.1		
**AquaOBP 2**	c29644.graph_c0	125	Complete ORF	22.75/0.61	odorant binding protein 05 [Tribolium castaneum]	EFA05677.1		
**AquaOBP C2**	c24269.graph_c0	169	Complete ORF	10.71/36.01	minus-C odorant binding protein 4 [Batocera horsfieldi]	ADD82417.1		
**AquaOBP 3**	c29196.graph_c1	142	Complete ORF	1.25/0.49	odorant binding protein 09 [Tribolium castaneum]	EFA10713.1		
**AquaOBP C3**	c29310.graph_c0	137	Complete ORF	0.55/190.4	minus-C odorant binding protein 4 [Batocera horsfieldi]	ADD82417.1		
**AquaOBP 4**	c29019.graph_c0	145	Complete ORF	1225.47/1.8	odorant binding protein 09 [Tribolium castaneum]	EFA10713.1		
**AquaOBP C4**	c35447.graph_c0	134	Complete ORF	0.83/33.47	minus-C odorant binding protein 4 [Batocera horsfieldi]	ADD82417.1		
**AquaOBP 5**	c24520.graph_c0	250	Complete ORF	2.94/3.3	odorant binding protein 11 [Tribolium castaneum]	EFA05695.1		
**AquaOBP C5**	c30996.graph_c0	135	Complete ORF	185.9/1.96	minus-C odorant binding protein 2 [Batocera horsfieldi]	ADD70031.1		
**AquaOBP 6**	c6376.graph_c0	150	Complete ORF	134.95/172.13	odorant binding protein 19 [Tribolium castaneum]	EFA02960.1		
**AquaOBP C6**	c24312.graph_c0	150	Complete ORF	184.45/105.61	odorant binding protein 1 [Monochamus alternatus]	AGH70097.1		
**AquaOBP 7**	c32760.graph_c0	153	Complete ORF	38.38/1.23	PREDICTED: general odorant-binding protein 28a [Tribolium castaneum]	XP_008200270.1		
**AquaOBP C7**	c18087.graph_c0	119	Complete ORF	0.02/0.75	odorant binding protein C20 [Tribolium castaneum]	EFA01425.1		
**AquaOBP 8**	c13307.graph_c0	152	Complete ORF	0.46/0	putative odorant binding protein [Stomoxys calcitrans]	ADG96060.1		
**AquaCSP 1**	c27044.graph_c0	117	Complete ORF	12.74/65.65	PREDICTED: Tribolium castaneum chemosensory protein 1 (Csp1), transcript variant X2, mRNA	XM_008202713.1	Yes	
**AquaCSP 2**	c24876.graph_c0	110	5' 3'lost	0.64/0	Tribolium castaneum chemosensory protein 10 (Csp10), mRNA	NM_001045813.1	No	
**AquaCSP 3**	c43925.graph_c0	107	Complete ORF	0.77/0	Tribolium castaneum chemosensory protein 8 (Csp8), mRNA	NM_001045825.1	Yes	
**AquaCSP 4**	c23773.graph_c0	129	Complete ORF	405.75/9.56	Tribolium castaneum chemosensory protein 12 (Csp12), mRNA	NM_001045815.1	Yes	
**AquaCSP 5**	c22795.graph_c0	138	Complete ORF	0.55/0.58	Tribolium castaneum chemosensory protein 11 (Csp11), mRNA	NM_001045814.1	Yes	
**AquaCSP 6**	c29991.graph_c0	120	Complete ORF	2.21/0	Tribolium castaneum chemosensory protein 5 (Csp5), mRNA	NM_001045822.1	Yes	
**AquaCSP 7**	c37487.graph_c0	318	Complete ORF	2.05/4.73	PREDICTED: Tribolium castaneum chemosensory protein 6 (Csp6), transcript variant X2, mRNA	XM_008195554.1	Yes	
**AquaCSP 8**	c42137.graph_c0	135	Complete ORF	915.66/11.27	Tribolium castaneum chemosensory protein 4 (Csp4), mRNA	NM_001045820.1	Yes	
**AquaCSP 9**	c29933.graph_c0	128	Complete ORF	119.04/4.45	PREDICTED: Tribolium castaneum chemosensory protein 7 (Csp7), transcript variant X1, mRNA	XM_008195555.1	Yes	
**AquaCSP 10**	c14115.graph_c0	122	Complete ORF	121.34/156.05	PREDICTED: Tribolium castaneum chemosensory protein 7 (Csp7), transcript variant X1, mRNA	XM_008195555.1	Yes	
**AquaOrco**	c41146.graph_c0	479	Complete ORF	40.85/18.62	PREDICTED: Tribolium castaneum odorant receptor coreceptor (LOC661975), mRNA	XM_008196471.1		7
**AquaOR 1**	c25389.graph_c0	209	5'lost	1.29/0	PREDICTED: Tribolium castaneum olfactory receptor 12 (LOC663463), mRNA	XM_008203047.1		3
**AquaOR 2**	c20513.graph_c0	221	5'lost	0.8/0	PREDICTED: Tribolium castaneum odorant receptor 9a-like (LOC103314420), mRNA	XM_008200489.1		2
**AquaOR 3**	c30597.graph_c0	353	5’lost	2.2/0	PREDICTED: Tribolium castaneum putative odorant receptor 71a (LOC103314297), mRNA	XM_008199934.1		7
**AquaOR 4**	c32448.graph_c0	423	Complete ORF	5.05/0.07	PREDICTED: Tribolium castaneum putative odorant receptor 71a (LOC103314297), mRNA	XM_008199934.1		7
**AquaOR 5**	c19494.graph_c0	194	5'lost	0.38/0.27	PREDICTED: Tribolium castaneum putative odorant receptor 71a (LOC103314297), mRNA	XM_008199934.1		3
**AquaOR 6**	c43305.graph_c0	364	Complete ORF	0.65/0	PREDICTED: Tribolium castaneum olfactory receptor 12 (LOC663463), mRNA	XM_008203047.1		6
**AquaOR 7**	c5656.graph_c0	106	5’3'lost	0.66/0.03	PREDICTED: Tribolium castaneum olfactory receptor 20 (LOC655178), mRNA	XM_961697.1		2
**AquaOR 8**	c35091.graph_c0	369	Complete ORF	2.1/0.34	PREDICTED: Tribolium castaneum odorant receptor (LOC100142012), mRNA	XM_001814810.1		5
**AquaOR 9**	c25921.graph_c0	405	Complete ORF	5.43/2.42	PREDICTED: Tribolium castaneum olfactory receptor 12 (LOC663463), mRNA	XM_008203047.1		6
**AquaOR 10**	c39137.graph_c0	327	Complete ORF	5.14/1.33	PREDICTED: Tribolium castaneum odorant receptor 25 (LOC100141798), mRNA	XM_001810453.2		5
**AquaOR 11**	c34947.graph_c0	247	3'lost	2.58/0.03	PREDICTED: Tribolium castaneum odorant receptor 67c-like (LOC103313327), mRNA	XM_008196326.1		4
**AquaOR 12**	c20227.graph_c0	128	5'lost	0.14/0.79	PREDICTED: Tribolium castaneum olfactory receptor 3 (LOC664552), mRNA	XM_008194185.1		3
**AquaOR 13**	c56184.graph_c0	107	5'lost	0.23/0	PREDICTED: Tribolium castaneum odorant receptor Or1-like (LOC103312189), mRNA	XM_008192202.1		2
**AquaOR 14**	c38388.graph_c0	249	5'lost	16.84/0.41	PREDICTED: Tribolium castaneum odorant receptor Or1-like (LOC103312189), mRNA	XM_008192202.1		2
**AquaOR 15**	c20583.graph_c0	378	Complete ORF	2.15/0.1	PREDICTED: Tribolium castaneum olfactory receptor 12 (LOC663463), mRNA	XM_008203047.1		5
**AquaOR 16**	c45693.graph_c0	167	5’3'lost	0.64/0	PREDICTED: Tribolium castaneum odorant receptor (LOC661659), transcript variant X2, mRNA	XM_008200026.1		2
**AquaOR 17**	c18751.graph_c0	314	Complete ORF	0.98/0	PREDICTED: Tribolium castaneum olfactory receptor 12 (LOC663463), mRNA	XM_008203047.1		5
**AquaOR 18**	c20804.graph_c0	377	Complete ORF	1.55/0.02	Tribolium castaneum or8 gene for olfactory receptor 8	AM689910.1		4
**AquaOR 19**	c7871.graph_c0	123	5’3'lost	0.76/0	PREDICTED: Tribolium castaneum odorant receptor Or1-like (LOC103312189), mRNA	XM_008192202.1		2
**AquaOR 20**	c32943.graph_c0	387	Complete ORF	1.59/0	PREDICTED: Tribolium castaneum odorant receptor (LOC100142012), mRNA	XM_001814810.1		7
**AquaOR 21**	c30258.graph_c0	148	5'lost	2.21/0.22	PREDICTED: Tribolium castaneum odorant receptor 85b-like (LOC103314252), mRNA	XM_008199720.1		3
**AquaOR 22**	c34706.graph_c0	372	Complete ORF	1.5/0.04	PREDICTED: Tribolium castaneum odorant receptor 49b-like (LOC100141914), mRNA	XM_001812209.1		6
**AquaOR 23**	c29459.graph_c0	323	Complete ORF	1.67/0.01	PREDICTED: Tribolium castaneum odorant receptor Or2-like (LOC103314064), mRNA	XM_008198934.1		5
**AquaOR 24**	c30896.graph_c0	375	Complete ORF	9.89/0.5	PREDICTED: Tribolium castaneum olfactory receptor 12 (LOC663463), mRNA	XM_008203047.1		6
**AquaOR 25**	c31144.graph_c0	129	3'lost	1.44/0.55	Tribolium castaneum or11 gene for olfactory receptor 11	AM689913.1		0
**AquaOR 26**	c11347.graph_c0	347	Complete ORF	2.95/0	PREDICTED: Tribolium castaneum olfactory receptor 15 (LOC100142153), mRNA	XM_008200490.1		6
**AquaOR 27**	c31817.graph_c0	291	5'lost	0.9/0.22	PREDICTED: Tribolium castaneum odorant receptor Or1-like (LOC103312189), mRNA	XM_008192202.1		5
**AquaOR 28**	c43416.graph_c0	151	5' 3'lost	0.68/0	PREDICTED: Tribolium castaneum odorant receptor Or1-like (LOC103312189), mRNA	XM_008192202.1		3
**AquaOR 29**	c34669.graph_c0	303	Complete ORF	5.34/0.29	PREDICTED: Tribolium castaneum odorant receptor (LOC661659), transcript variant X1, mRNA	XM_967808.2		5
**AquaOR 30**	c22874.graph_c0	256	3'lost	0.98/0	PREDICTED: Tribolium castaneum odorant receptor 85b-like (LOC103314252), mRNA	XM_008199720.1		4
**AquaOR 31**	c27286.graph_c0	341	Complete ORF	0.35/2.82	PREDICTED: Tribolium castaneum odorant receptor 67c-like (LOC103313327), mRNA	XM_008196326.1		5
**AquaOR 32**	c31772.graph_c0	380	Complete ORF	20.18/1.63	PREDICTED: Tribolium castaneum odorant receptor 46a, isoform B-like (LOC103312238), mRNA	XM_008192395.1		6
**AquaOR 33**	c35731.graph_c1	392	Complete ORF	2.03/0.91	PREDICTED: Tribolium castaneum olfactory receptor 12 (LOC663463), mRNA	XM_008203047.1		5
**AquaIR 1**	c30209.graph_c0	512	5'3'lost	1.5/0.24	PREDICTED: Tribolium castaneum glutamate receptor ionotropic, NMDA 2B (LOC660406), transcript variant X2, mRNA	XM_008193055.1		1
**AquaIR 2**	c46148.graph_c0	111	5'3'lost	0.12/0.33	PREDICTED: Tribolium castaneum glutamate receptor ionotropic, kainate 2-like (LOC655107), mRNA	XM_008192857.1		0
**AquaIR 3**	c27639.graph_c0	255	5'lost	0.66/0.17	PREDICTED: Tribolium castaneum glutamate receptor ionotropic, NMDA 2B (LOC660406), transcript variant X1, mRNA	XM_008193054.1		2
**AquaIR 4**	c38283.graph_c0	890	Complete ORF	0.46/2.95	PREDICTED: Tribolium castaneum glutamate receptor ionotropic, kainate 2-like (LOC663773), mRNA	XM_008203439.1		3
**AquaIR 5**	c34644.graph_c0	889	Complete ORF	17.27/59.19	PREDICTED: Tribolium castaneum glutamate receptor ionotropic, kainate 2-like (LOC655031), transcript variant X3, mRNA	XM_961527.2		3
**AquaIR 6**	c36987.graph_c0	423	5'lost	9.84/74.13	PREDICTED: Tribolium castaneum glutamate receptor ionotropic, kainate 3-like (LOC657048), transcript variant X2, mRNA	XM_008200236.1		3
**AquaIR 7**	c40757.graph_c0	485	3'lost	3.73/34.03	PREDICTED: Tribolium castaneum glutamate receptor ionotropic, kainate 2-like (LOC655031), transcript variant X3, mRNA	XM_961527.2		1
**AquaIR 8a**	c33131.graph_c0	721	Complete ORF	43.23/9.22	PREDICTED: Tribolium castaneum glutamate receptor ionotropic, kainate 2 (LOC656746), mRNA	XM_963253.2		3
**AquaIR 9**	c36130.graph_c0	343	5'3'lost	1.75/0.15	PREDICTED: Tribolium castaneum glutamate receptor ionotropic, kainate 2-like (LOC655262), mRNA	XM_961791.3		1
**AquaIR 10**	c46223.graph_c0	158	5’3'lost	0.23/0.31	PREDICTED: Tribolium castaneum glutamate receptor ionotropic, kainate 2-like (LOC655107), mRNA	XM_008192857.1		1
**AquaIR 11**	c36036.graph_c0	822	Complete ORF	3.15/13.03	PREDICTED: Tribolium castaneum glutamate receptor ionotropic, kainate 2-like (LOC663805), mRNA	XM_969840.2		3
**AquaIR 12**	c40210.graph_c1	260	3'lost	3.68/30.82	PREDICTED: Tribolium castaneum glutamate receptor ionotropic, kainate 3-like (LOC657048), transcript variant X1, mRNA	XM_008200235.1		2
**AquaIR 13**	c37308.graph_c0	143	5'lost	2.05/0.37	PREDICTED: Tribolium castaneum glutamate receptor ionotropic, kainate 5 (LOC658055), mRNA	XM_008197243.1		1
**AquaIR 14**	c23242.graph_c0	250	5'3'lost	1.15/0.21	PREDICTED: Tribolium castaneum glutamate receptor ionotropic, kainate 2-like (LOC655262), mRNA	XM_961791.3		1
**AquaIR 15**	c48352.graph_c0	142	5'3'lost	0.56/0	PREDICTED: Tribolium castaneum glutamate receptor ionotropic, kainate 2-like (LOC663773), mRNA	XM_008203439.1		0
**AquaIR 16**	c38169.graph_c0	798	Complete ORF	2.47/24.34	PREDICTED: Tribolium castaneum glutamate receptor ionotropic, kainate 2 (LOC654966), mRNA	XM_961435.1		3
**AquaIR 25a**	c35322.graph_c0	922	Complete ORF	24.81/0.49	PREDICTED: Tribolium castaneum glutamate receptor ionotropic, kainate 2 (LOC659899), mRNA	XM_008202574.1		3
**AquaIR 41a**	c37132.graph_c0	571	3'lost	1.63/0.07	PREDICTED: Tribolium castaneum glutamate receptor ionotropic, delta-2 (LOC663559), mRNA	XM_008203436.1		1
**AquaIR 64a**	c25275.graph_c0	185	5'3'lost	1.46/0.67	PREDICTED: Tribolium castaneum glutamate receptor ionotropic, kainate 3-like (LOC657048), transcript variant X2, mRNA	XM_008200236.1		1
**AquaIR 75q**	c49298.graph_c0	129	5'lost	0.46/0	PREDICTED: Tribolium castaneum glutamate receptor ionotropic, kainate 2-like (LOC663773), mRNA	XM_008203439.1		1
**SNMP 1**	c37873.graph_c0	520		99.28/31.58	PREDICTED: Tribolium castaneum sensory neuron membrane protein 1 (LOC100142232), mRNA	XM_001816384.2		
	c39123.graph_c0	514		253.37/134.02	PREDICTED: Tribolium castaneum sensory neuron membrane protein 1 (LOC100142232), mRNA	XM_001816384.2		
	c6003.graph_c0	502		10.57/6.57	PREDICTED: Tribolium castaneum sensory neuron membrane protein 1 (LOC100142232), mRNA	XM_001816384.2		
	c28645.graph_c0	384		0.43/0.58	PREDICTED: Tribolium castaneum sensory neuron membrane protein 1-like (LOC100142034), mRNA	XM_001816388.2		
	c28834.graph_c0	474		0.5/0	PREDICTED: Tribolium castaneum sensory neuron membrane protein 1-like (LOC100142034), mRNA	XM_001816388.2		
	c39779.graph_c0	282		3.91/1.32	PREDICTED: Tribolium castaneum sensory neuron membrane protein 1-like (LOC100142034), mRNA	XM_001816388.2		
	c24728.graph_c0	299		0.89/0	PREDICTED: Tribolium castaneum sensory neuron membrane protein 1-like (LOC100141821), mRNA	XM_001816389.2		
	c36561.graph_c0	540		132.64/3.52	PREDICTED: Tribolium castaneum sensory neuron membrane protein 1-like (LOC100141821), mRNA	XM_001816389.2		
	c37047.graph_c0	536		221.73/108.11	PREDICTED: Tribolium castaneum sensory neuron membrane protein 1-like (LOC100141821), mRNA	XM_001816389.2		
	c39598.graph_c0	295		62.88/58.74	PREDICTED: Tribolium castaneum sensory neuron membrane protein 1-like (LOC100141821), mRNA	XM_001816389.2		
	c61337.graph_c0	35		0.33/0	PREDICTED: Tribolium castaneum sensory neuron membrane protein 1-like (LOC100141821), mRNA	XM_001816389.2		
**SNMP 2**	c32778.graph_c0	406		1.25/0	PREDICTED: Tribolium castaneum sensory neuron membrane protein 2 (LOC658533), mRNA	XM_964915.2		
	c35000.graph_c0	292		240.78/79.14	PREDICTED: Tribolium castaneum sensory neuron membrane protein 2 (LOC658533), mRNA	XM_964915.2		
	c35827.graph_c0	488		157.2/73.47	PREDICTED: Tribolium castaneum sensory neuron membrane protein 2 (LOC658533), mRNA	XM_964915.2		
	c37683.graph_c0	518		27.9/12.89	PREDICTED: Tribolium castaneum sensory neuron membrane protein 2 (LOC658533), mRNA	XM_964915.2		
	c39298.graph_c0	542		7.84/19.58	PREDICTED: Tribolium castaneum sensory neuron membrane protein 2 (LOC658533), mRNA	XM_964915.2		
	c45852.graph_c0	86		0.09/0.46	PREDICTED: Tribolium castaneum sensory neuron membrane protein 2 (LOC658533), mRNA	XM_964915.2		
	c57679.graph_c0	57		0.39/0	PREDICTED: Tribolium castaneum sensory neuron membrane protein 2 (LOC658533), mRNA	XM_964915.2		
	c28688.graph_c0	190		0.99/0	PREDICTED: Tribolium castaneum sensory neuron membrane protein 2 (LOC658533), mRNA	XM_964915.2		
	c69846.graph_c0	54		0.17/0	PREDICTED: Tribolium castaneum sensory neuron membrane protein 2 (LOC658533), mRNA	XM_964915.2		
	c16552.graph_c0	123		0.42/0	PREDICTED: Tribolium castaneum sensory neuron membrane protein 2-like (LOC103314488), mRNA	XM_008200727.1		
	c50846.graph_c0	56		0.47/0	PREDICTED: Tribolium castaneum sensory neuron membrane protein 2-like (LOC103314488), mRNA	XM_008200727.1		
	c34299.graph_c0	152		6.09/0.03	PREDICTED: Tribolium castaneum sensory neuron membrane protein 2-like (LOC103314494), mRNA	XM_008200740.1		
	c34299.graph_c1	353		6.99/0.04	PREDICTED: Tribolium castaneum sensory neuron membrane protein 2-like (LOC103314494), mRNA	XM_008200740.1		
	c38331.graph_c0	573		27.69/10.37	PREDICTED: Tribolium castaneum sensory neuron membrane protein 2-like (LOC103314494), mRNA	XM_008200740.1		
	c50128.graph_c0	70		0.55/0	PREDICTED: Tribolium castaneum sensory neuron membrane protein 2-like (LOC103314494), mRNA	XM_008200740.1		
	c51674.graph_c0	110		0.56/0	PREDICTED: Tribolium castaneum sensory neuron membrane protein 2-like (LOC103314494), mRNA	XM_008200740.1		
	c8230.graph_c0	44		0.33/0	PREDICTED: Tribolium castaneum sensory neuron membrane protein 2-like (LOC103314494), mRNA	XM_008200740.1		

### Motif pattern analysis of Candidate OBPs and CSPs

**Fig 2:**
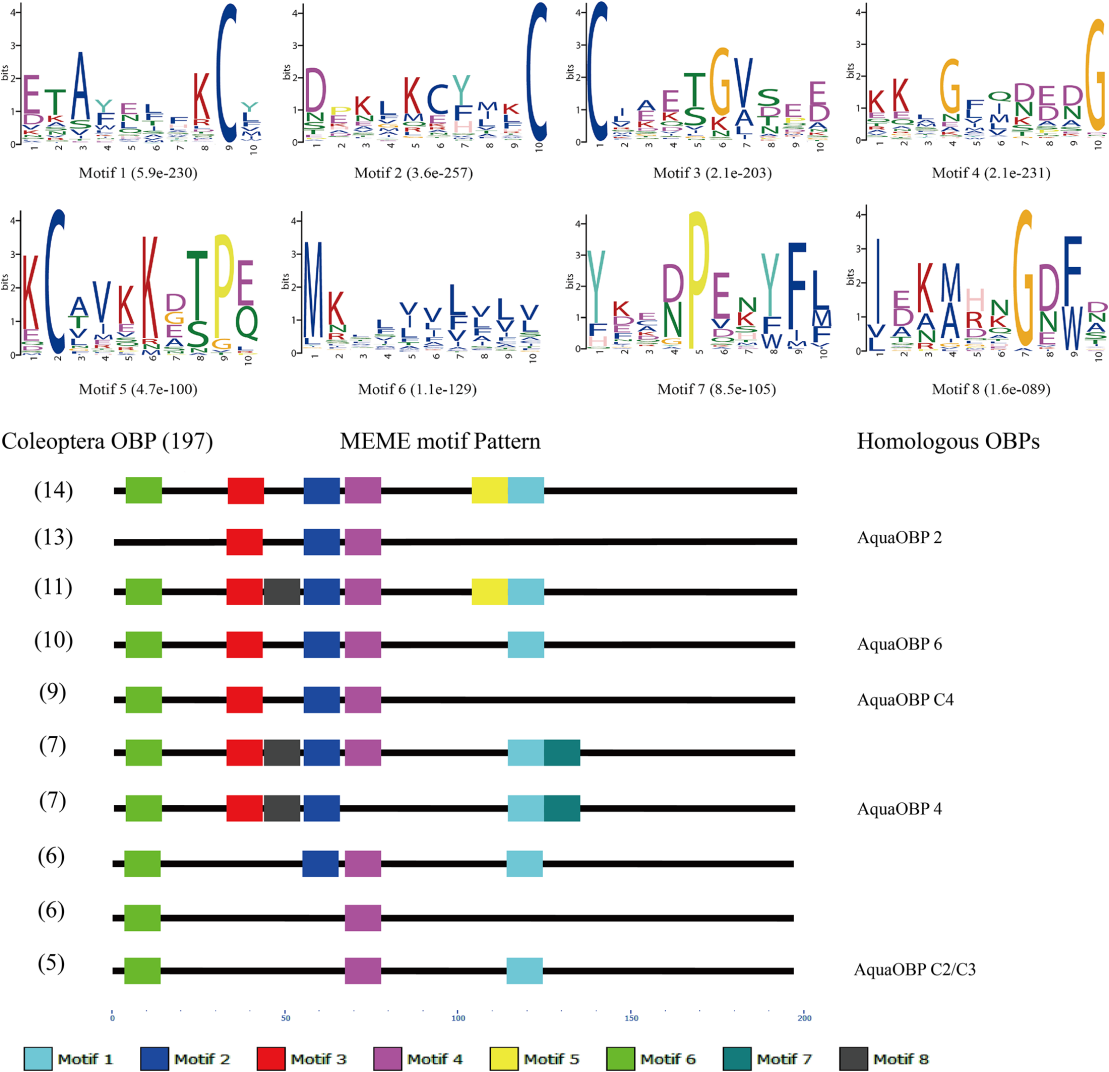
Motif analysis of coleopteran OBPs. Parameters used for motif analysis were minimum width = 6, maximum width = 10, and maximum number of motifs to find = 8. Different colored squares indicate the type and approximate location of each motif on the protein sequence, starting from the N-terminal.

Eight motifs with high E-values were discovered from 197 Coleoptera OBPs, and the 10 most common motif patterns were identified. Motif 6 and motif 1 were highly conserved and existed in nearly all of the common patterns except pattern 2 and pattern 4, as well as motif 3, motif 4, motif 8 and motif 2. Motif 5, motif 1 and motif 7 clustered together, whereas motif 6 is localized separately on the N-terminus. The most common pattern in the coleopteran OBPs was 6-3-2-4-5-1 with a percentage of 7.1% (14/197), which suggested a comparative pattern varies in Coleoptera OBPs. Moreover, AquaOBP2 showed a motif of pattern 3-2-4 (13/197), and AquaOBP6 had a 6-3-2-4-1 (10/197) motif. AquaOBPC4 had 6-3-2-4 (9/197) motif and AquaOBP4 a 6-3-8-2-1-7 (7/197) motif. Finally, AquaOBPC2 and AquaOBPC3 shared the same 6-4-1 (5/197) motif, whereas the motif pattern of the remaining AquaOBPs motif patterns exhibited more variety (Fig 2).

**Fig 3:**
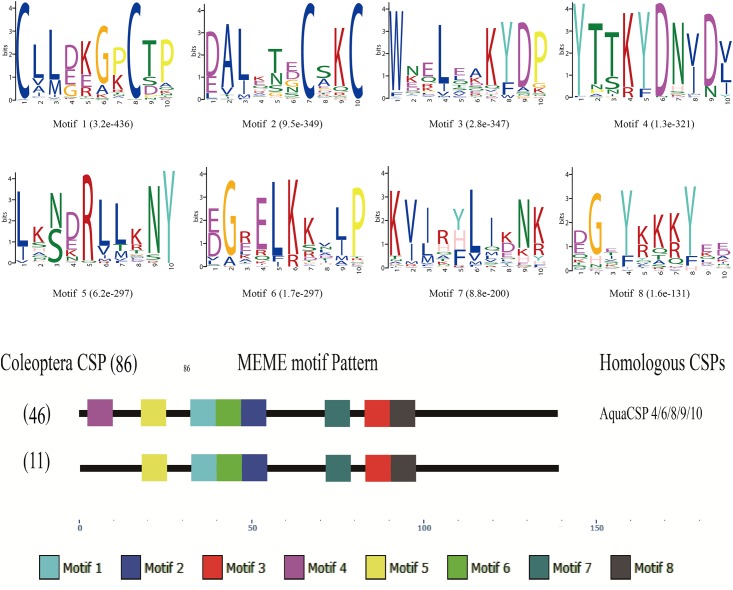
Motif analysis of coleopteran CSPs. Parameters used for motif analysis were minimum width = 6, maximum width = 10, and maximum number of motifs to find = 8. Different colored squares indicate the type and approximate location of each motif on the protein sequence, starting from the N-terminal.

Compared with OBPs, the CSP motif patterns in Coleoptera were more highly conserved with two common patterns were discovered at a percentage of 66.28% (55/86). The most common CSP patterns were 4-5-1-6-2-7-3-8 (46/86), and AquaCSP 4/6/8/9/10 contained this pattern. The second most common pattern was 5-1-6-2-7-3-8, which is missing motif 4 at the N-terminus. No AquaCSPs were observed with this last motif pattern (Fig 3).

It is interesting that motif 6 in coleopteran OBPs had high similarity (E-value 6.9e-4) with Andropin sequences in Drosophila mauritiana, and motif 7 showed similarity (E-vlaue 1.7e-1) with B-adaptin in Arabidopsis thaliana. These two proteins were previously identified as components in transmembrane signal transduction.

### Phylogenetic Analysis of Candidate Olfactory Genes

**Fig 4:**
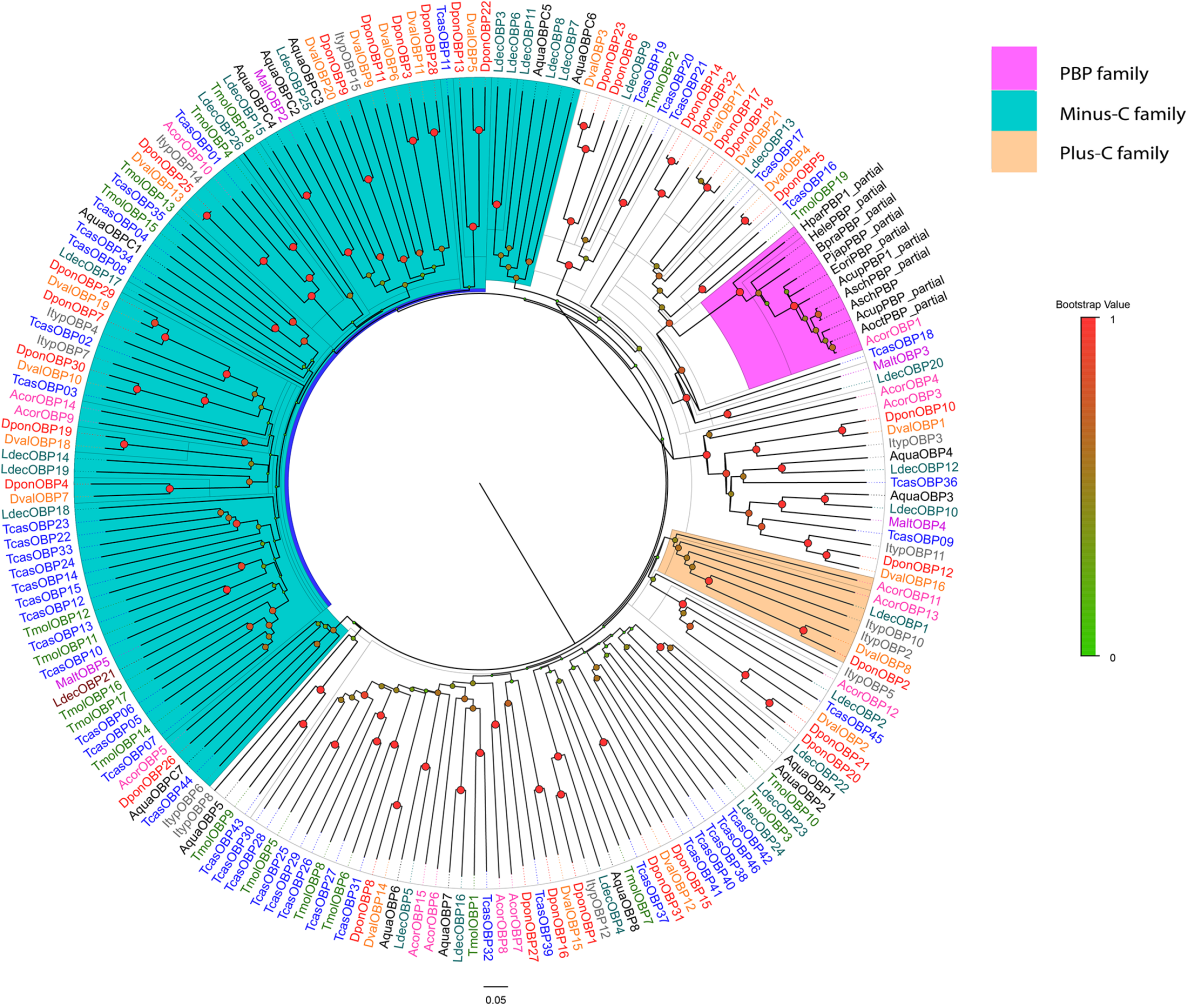
Neighbor-joining tree of AquaOBPs. Values indicated at the nodes are bootstrap values based on 1000 replicates; scale bar = 0.1. Aqua: *Ambrostoma quadriimpressum*; Tcas: *Tribolium castaneum*; Dpon: *Dendroctonus ponderosae*; Malt: *Monochamus alternatus*; Ldec: *Leptinotarsa decemlineata*; Ityp: *Ips typographus*; Tmol: *Tenebrio molitor*; Acor: *Anomala corpulenta*. Dval: *Dendroctonus valens*; Hpar: *Holotrichia parallela*; Hele: *Hylamorpha elegans*; Bpra: *Brachysternus prasinus*; Pjap: *Popillia japonica*; Eori: *Exomala orientalis*; Acup: *Anomala cuprea*; Asch: *Anomala schonfeldti*; Aoct: *Anomala octiescostata*. Different colors indicate the types of OBPs.

**Fig 5:**
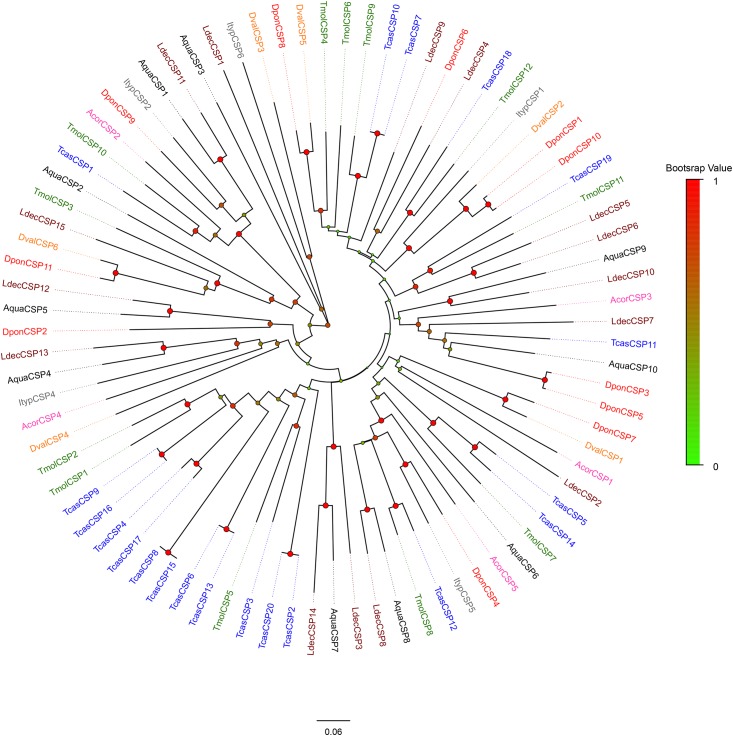
Neighbor-joining tree of AquaCSPs. Values indicated at the nodes are bootstrap values based on 1000 replicates; scale bar = 0.1. Aqua: *Ambrostoma quadriimpressum*; Tcas: *Tribolium castaneum*; Dpon: *Dendroctonus ponderosae*; Ityp: *Ips typographus*; Ldec: *Leptinotarsa decemlineata*; Tmol: *Tenebrio molitor*; Acor: *Anomala corpulenta*; Dval: *Dendroctonus valens*.

Sixteen candidate OBPs sorted phylogenetically as expected based on their highest sequence homology. Seven minus-C OBPs from *A*. *quadriimpressum* grouped together with the minus-C OBPs of other coleopteran species, whereas nine conserved OBPs of *A*. *quadriimpressum* were grouped together. No PBP or Plus-C family orthologs were observed in *A*. *quadriimpressum* (Fig 4). For CSPs, AquaCSP9 and AquaCSP10, AquaCSP6 and AquaCSP8, and AquaCSP4 and AquaCSP5 grouped into a single clade, whereas the other seven CSPs separated into different clades (Fig 5).

**Fig 6:**
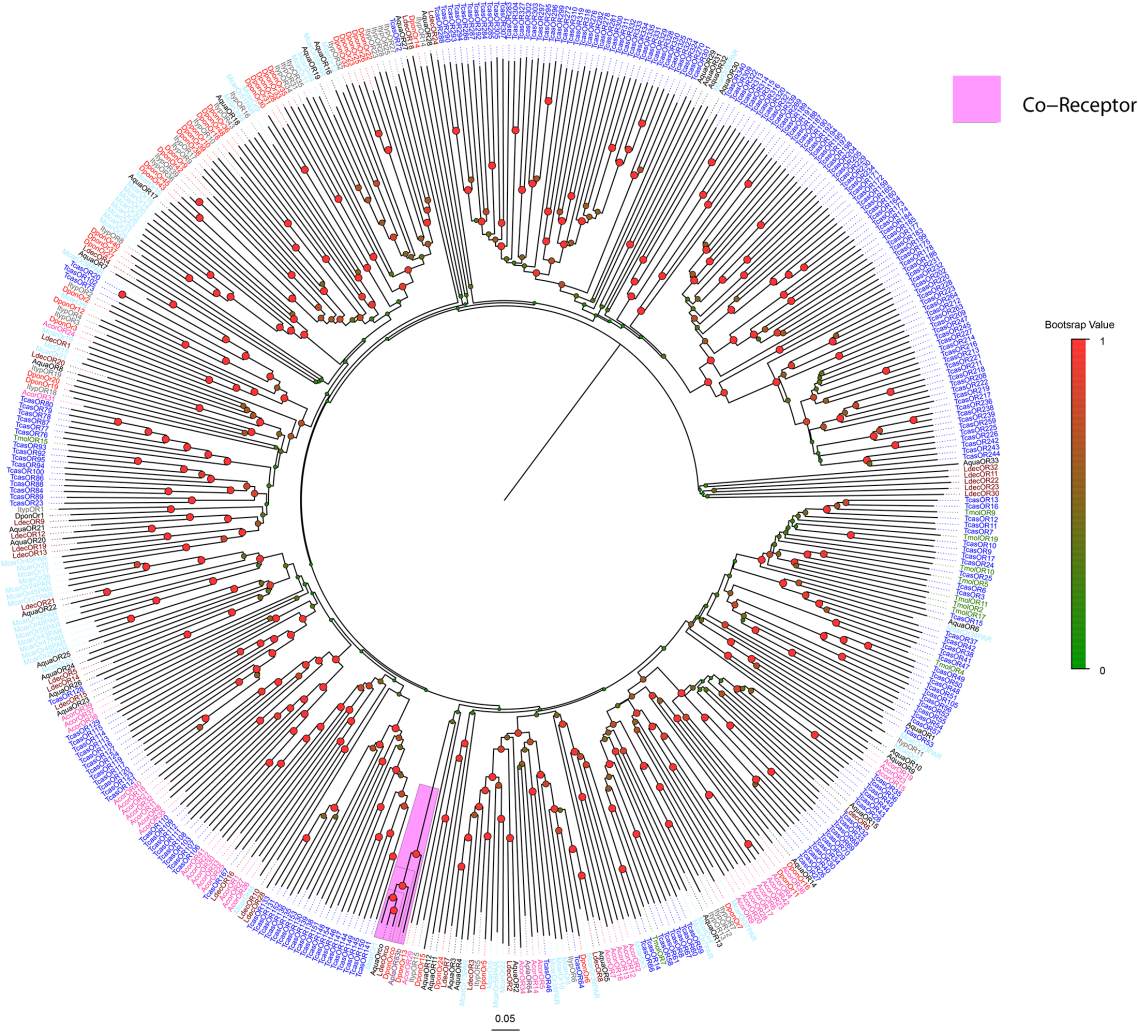
Neighbor-joining tree of AquaORs. Values indicated at the nodes are bootstrap values based on 1000 replicates; scale bar = 0.1. Aqua: *Ambrostoma quadriimpressum*; Tcas: *Tribolium castaneum*; Dpon: *Dendroctonus ponderosae*; Malt: *Monochamus alternatus*; Ldec: *Leptinotarsa decemlineata*; Ityp: *Ips typographus*; Mcar: *Megacyllene caryae*; Tmol: *Tenebrio molitor*; Acor: *Anomala corpulenta*. Dval: *Dendroctonus valens*; *Agla*: *Anoplophora glabripennis*. Purple indicates the Orco family.

**Fig 7:**
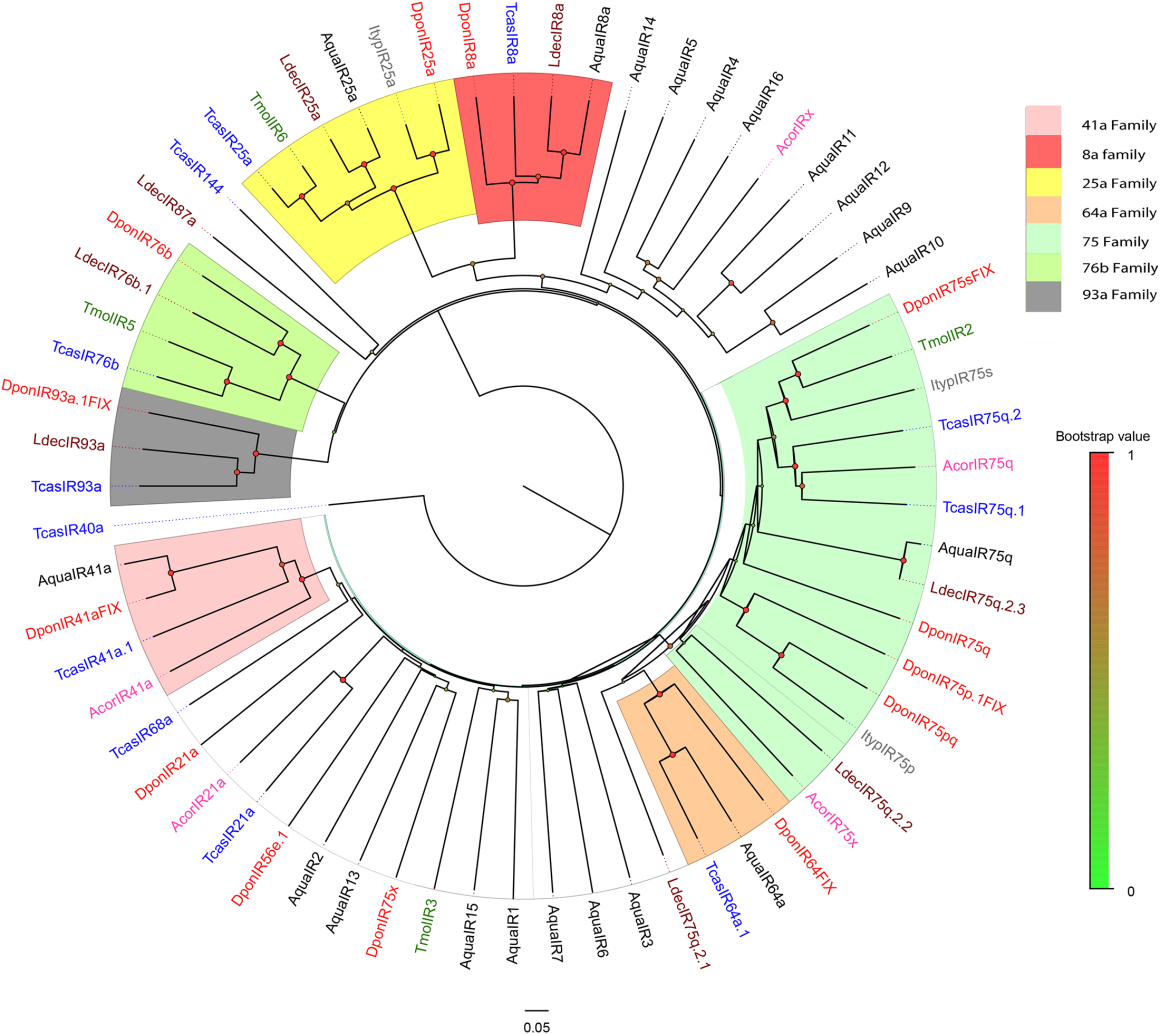
Neighbor-joining tree of AquaIRs. Values indicated at the nodes are bootstrap values based on 1000 replicates, scale bar = 0.1, Aqua: *Ambrostoma quadriimpressum*; Tcas: *Tribolium castaneum*; Dpon: *Dendroctonus ponderosae*; Malt: *Monochamus alternatus*; Ldec: *Leptinotarsa decemlineata*; Ityp: *Ips typographus*; Mcar: *Megacyllene caryae*; Tmol: *Tenebrio molitor*; Acor: *Anomala corpulenta*. Different color ranges indicate different IR families in reference to IR genes of *Drosophila melanogaster*.

Thirty-four candidate ORs were identified including AquaORCO. AquaOr29/30/31/32 were grouped together and are orthologs of McOR50. AquaOR11 and AquaOR12 are orthologs of DponOR15 and ItypOR15, and AquaOR9 and AquaOR10 are orthologs of ItypOR11 and McarOr2. Additionally, AquaOR8, AquaOR20 and AquaOR21 grouped into same clade (Fig 6). However, because of the lack of de-orphanized ORs in coleopteran insects, the biological function of these candidate ORs requires further exploration. For IRs, IR8a, IR25a, IR41a, IR64a and IR75 orthologues were identified in *A*. *quadriimpressum*, but no IR68a, IR76b and IR93a orthologues were found (Fig 7). Additionally, 15 candidate IRs clustered away from known families, and we defined them as “unnamed or separated clades”. AquaIR4/5/6/9/10/11/12/14 were grouped together with AcorIRx. AquaIR8a and AquaIR25a were identified in *A*. *quadriimpressum*. According to previous research, these two IRs were phylogenetically highly conserved and are considered to function as co-receptors [31, 32]. AquaIR64a orthologs in *Drosophila melanogaster* were shown to be required for acid avoidance behavior [33].

**Fig 8:**
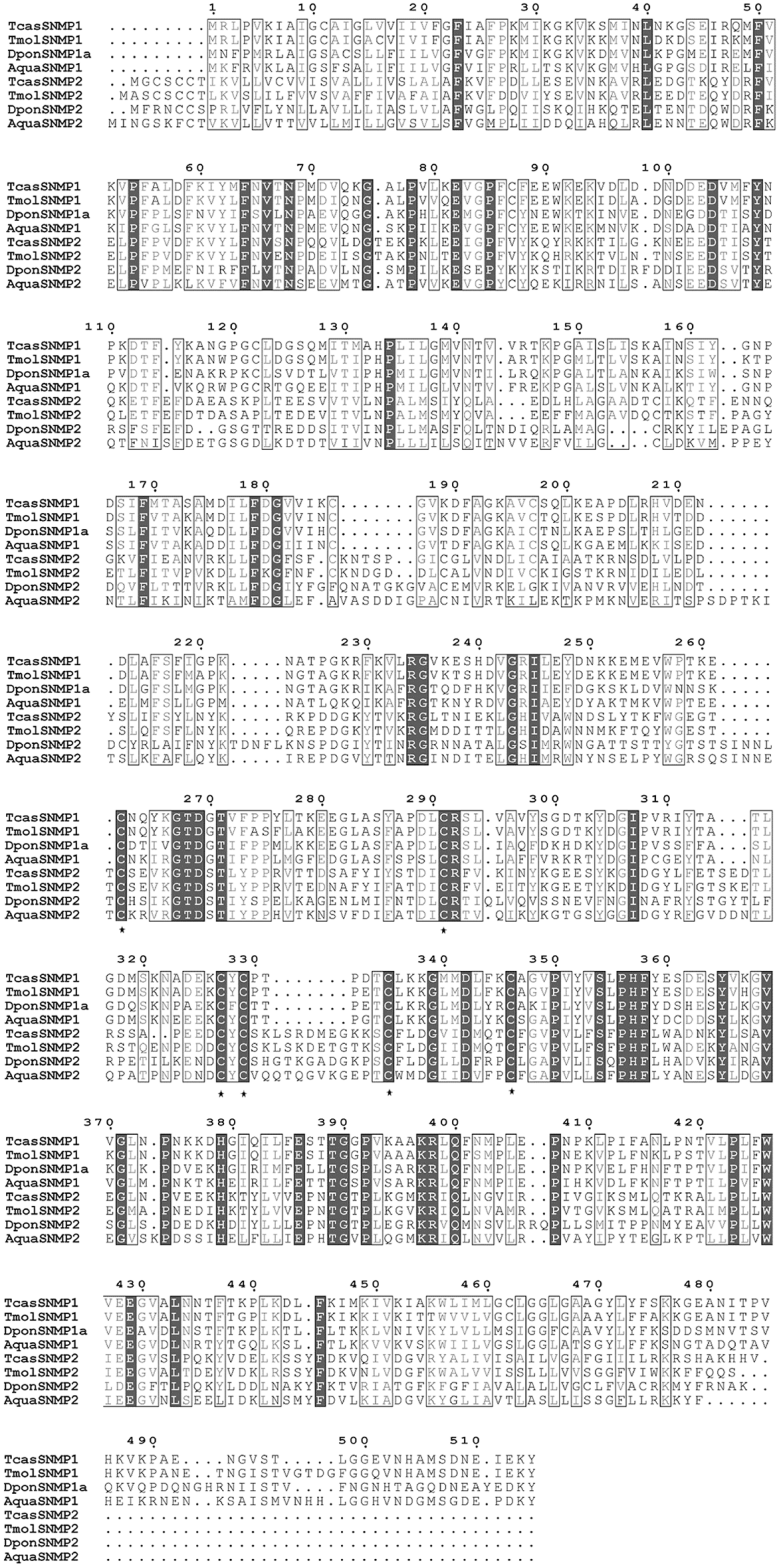
Alignment of AquaSNMPs with other coleopteran species. Aqua: *Ambrostoma quadriimpressum*; Tcas: *Tribolium castaneum*; Dpon: *Dendroctonus ponderosae*; Tmol: *Tenebrio molitor*. The dark background indicates highly conserved sites, other conserved sites are marked with boxes, * indicates a conserved cysteine site.

In total, 28 candidate SNMPs and their isoforms were identified. The amino acid sequences of two complete ORF unigenes were aligned with SNMP sequences of other coleopteran species; as a result, AquaSNMP1 and AquaSNMPs were more similar to *Tenebrio molitor* SNMPs with identities of 58% and 42%, respectively. Six conserved serines were identified in AquaSNMPs, and another 52 highly conserved sites were also observed (Fig 8).

### Tissue- and Sex-specific Expressions of Candidate Olfactory Genes

Referring to the calculated expression values determined based on the FKPM methods, 52 candidate olfactory genes were selected to further explore tissue- and sex-specific expression. The selection standard was as follows: the expression level of the gene must be sufficiently high in the antenna (olfactory organs) or the legs (non-olfactory organs) for these genes to have important functions during the insect’s lifespan. Additionally, if a gene was identified with a complete ORF to ensure the functional activity of the gene, it was included. As a result, 15 OBPs, 9 CSPs, 17 ORs, 9 IRs and 2 SNMPs were chosen for further validation.

**Fig 9:**
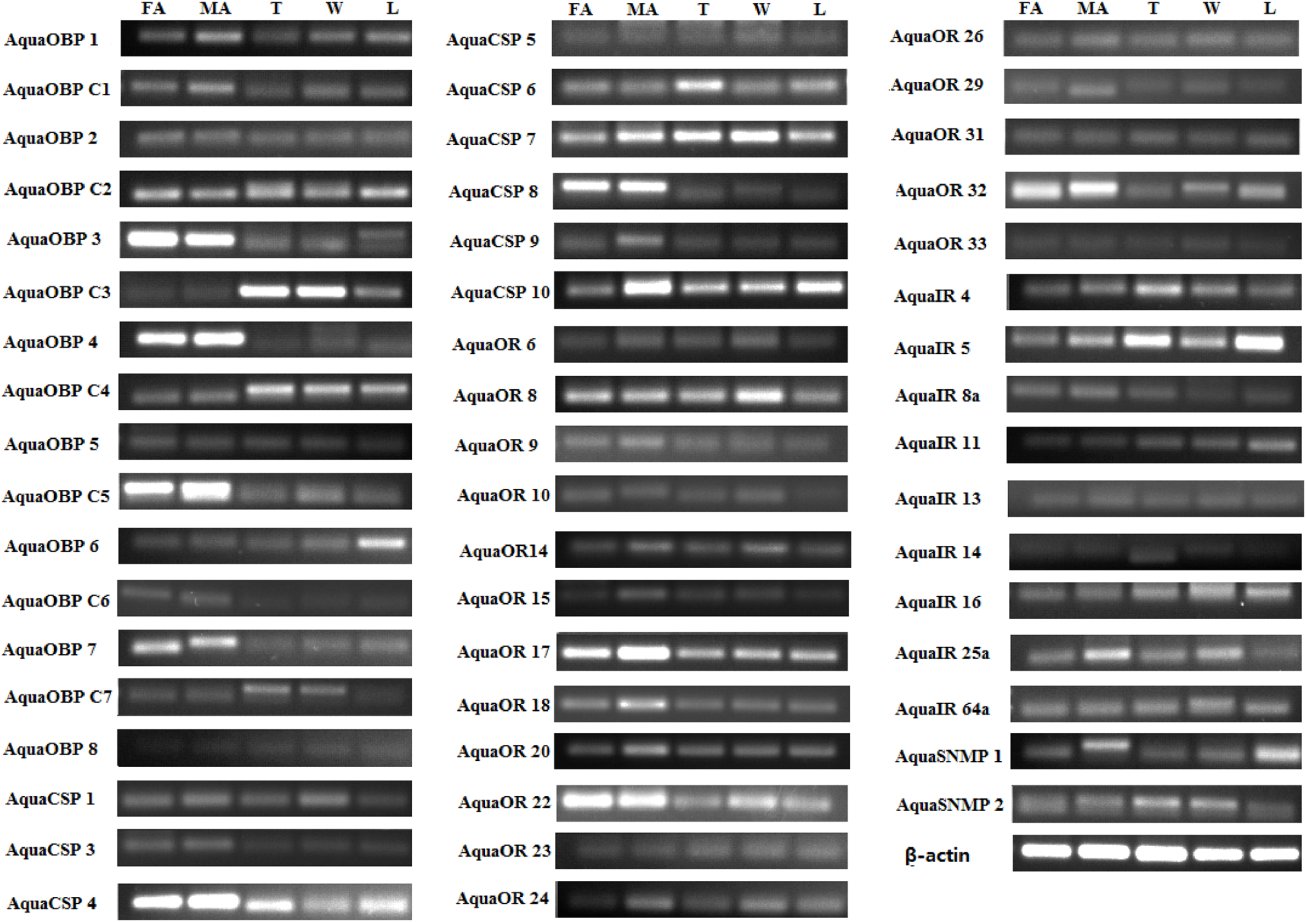
RT-PCR results of candidate olfactory genes of *A*. *quadriimpressum*. FA: female antenna; MA: male antenna; FL: female leg; ML: male leg; T: Thorax; W: hind wing. ß-actin was used as an internal control.

Semi-quantitative RT-PCR showed that all of the tested candidate olfactory genes were primarily male specific (Fig 9). AquaOBPC2/C3/C4/C7, AquaOBP8/C2/C3/C4/C7, AquaCSP5/6/7/10, AquaOR6/23/24/31, AquaIR4/5/11/14/16/64a showed non-olfactory specific expression, whereas AquaOBP1/2/4/7/C1/C5/C6, AquaCSP1/3/4/8/9, AquaOR9/10/14/15/17/20/22/26/29/32/33, AquaIR13/25a showed olfactory-specific expression. quaOBP4/C5, AquaCSP7/9/10, AquaOR17/24/32 and AquaIR4 were highly expressed in the antenna of males, suggesting that these genes are likely related to sex-specific behaviors in *A*. *quadriimpressum*, and unsurprisingly, the co-receptors AquaIR8a and AquaIR25a showed high and non-specific expression patterns.

**Fig 10:**
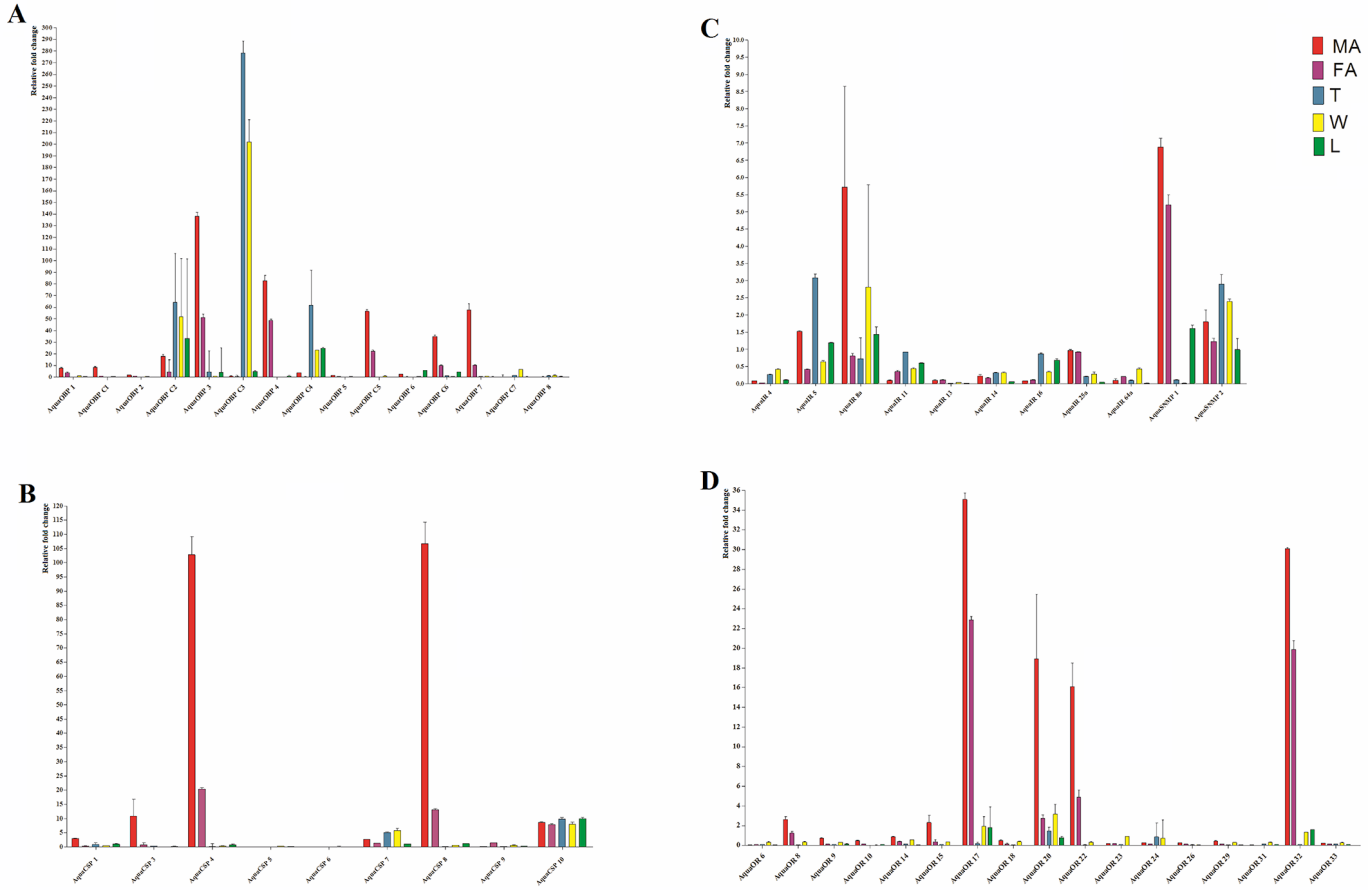
RT-qPCR results of candidate olfactory genes of *A*. *quadriimpressum*. RT-qPCR data analysis was performed using the 2^-ΔΔCT^ method. Error bars indicate the SEM. FA: female antenna; MA: male antenna; FL: female leg; ML: male leg; T: Thorax; W: hind wing. ß-actin was used as an internal control.

RT-qPCR results were mostly consistent with the RT-PCR results, AquaOBP6/8/C2/C3/C4/C7, AquaCSP5/6/7/10, AquaOR6/23/24/31 and AquaIR4/5/11/14/16/64a showed highly non-olfactory expression, whereas AquaOBP1/2/3/4/7/C1/C5/C6, AquaCSP1/3/4/8/9 and AquaOR8/9/10/14/15/17/18/20/22/26/29/32/33 showed highly olfactory-specific expression. A highly male-specific expression trend was observed for all of the candidate olfactory genes. Co-receptors AquaIR8a and AquaIR25a showed high expression levels in both males and females (Fig 10).

**Fig 11:**
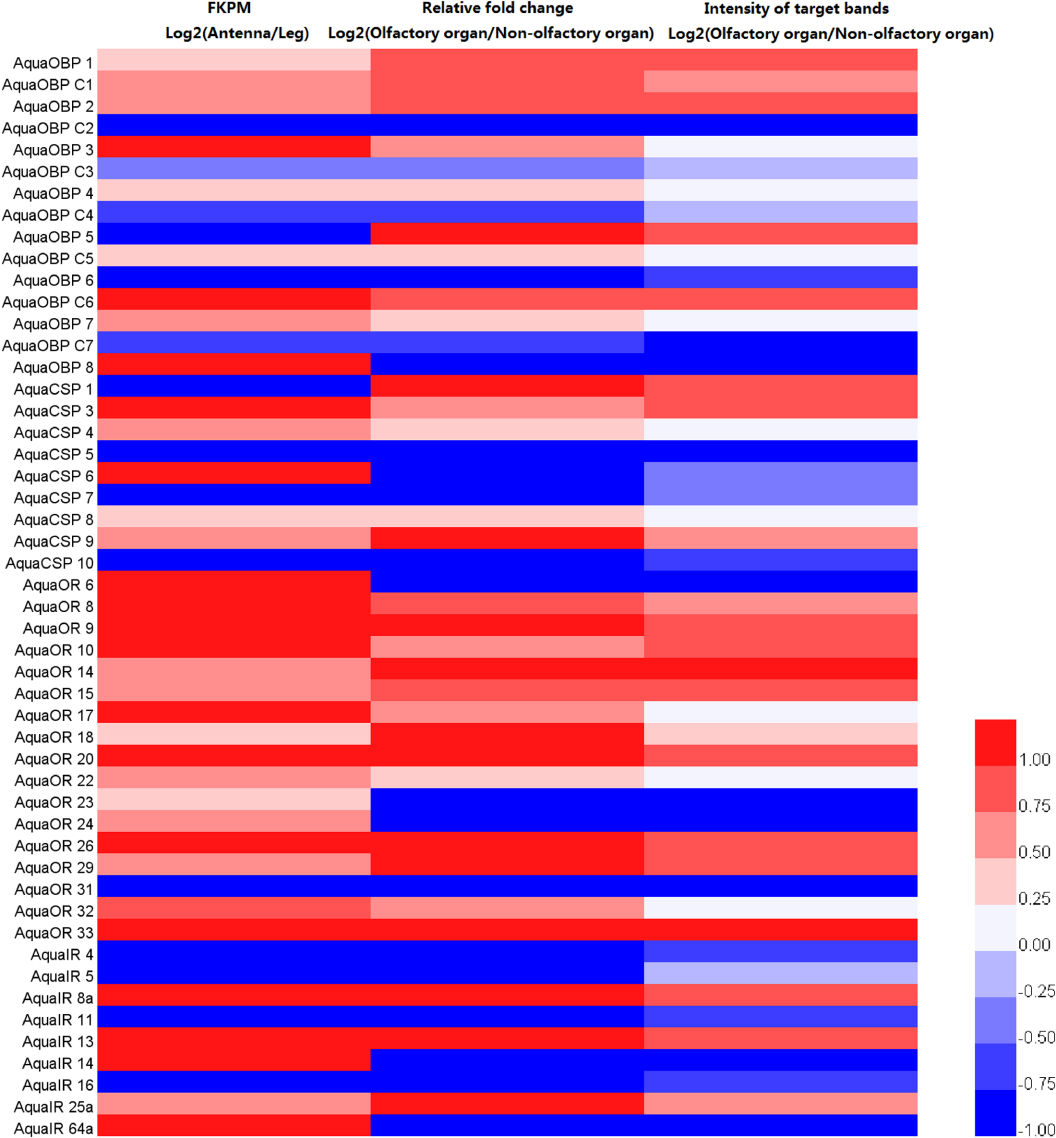
Expression level heatmap of RNA-Seq, RT-PCR and RT-qPCR results. Expression data were normalized to ±1 by their range. Values > 0, indicate that the gene has olfactory specificity and is colored red, whereas values < 0, indicate that the gene has non-olfactory specificity and is colored blue. Light and dark colors indicate the ratio of expression level (Antenna/ Non-olfactory organs).

Finally, combined with the results of three different experiments, the decision of which genes are olfactory or non-olfactory expressed was made only when the results of all three experiments were consistent with each other. As a result, AquaOBP1/2/4/7/C1/C6, AquaCSP3/9, AquaOR8/9/10/14/15/18/20/26/29/33 and AquaIR8a/13/25a showed olfactory-specific expression, whereas AquaOBP6/C2/C3/C4/C7, AquaCSP5/7/10, AquaOR31 and AquaIR4/5/11/16 showed non-olfactory-specific expression. The remaining genes either showed no obvious organ specific expression or the results of the three methods were in conflict (Fig 11). RT-qPCR results were consistent with the RT-PCR results, but conflicted with results of the FKPM analyses in 9 of the 50 genes including AquaOBP5/8. AquaCSP1/6, AquaOR6/23/24 and AquaIR14/64a. To determine the reason for the discrepancy, we double-checked the RT-qPCR and FKPM values. For AquaOBP8, AquaCSP6, AquaOR6/23/24, and AquaIR14/64a, the expression levels in thoracic muscle and hindwings were much higher than that in antennae. The calculated non-olfactory RT-PCR and RT-qPCR results were averaged across leg, thorax, and hindwings, whereas the RNA-Seq data were based on a single leg. This variation likely caused the observed mismatch.

## Discussion

Based on deep RNA sequencing of the antenna and legs, we identified 16 candidate OBPs, 10 candidate CSPs, 34 candidate ORs, 20 candidate IRs and 2 candidate SNMPs of *A*. *quadriimpressum*. To the best of our knowledge, the molecular underpinnings of the olfactory system of Coleoptera are relatively unknown, and only a few species’ olfactory genes have been identified, such as those of bark beetles [11, 13, 17], long-horned beetles [14, 18, 19], scarab beetles [12, 15] and flour beetles [16]. This is the first comprehensive study of olfactory genes in the elm pest *A*. *quadriimpressum*. Most of the identified olfactory genes were found to be complete ORFs according to their lengths and structures; these data could contribute information about this process for future functional studies.

Annotation data showed that the number of genes in antenna with binding and receptor activities was greater than that of the legs. Many pathway genes were differentially expressed in the two organs, which strongly supported the olfactory-sensing role of the antenna at a transcriptional level. The calculated expression levels based on the FKPM method showed that a lot of the identified candidate olfactory genes were more or less antenna specific rather than non-olfactory organs. In previous research, the olfactory genes that were highly expressed in antenna but not non-olfactory organs were more likely to have a direct effect on the olfactory sensing process within a species [34]. Thus, most of the candidate olfactory genes identified were involved in the chemosensory process in *A*. *quadriimpressum*. A large number of olfactory genes were found to be specifically expressed in the antenna of *A*. *quadriimpressum*. Compared with other species, whose lives are highly reliant on olfaction, such as *Anopheles gambiae* and *Drosophila melanogaster*, the number of identified active olfactory genes in *A*. *quadriimpressum* was much lower (82 olfactory genes in *A*. *quadriimpressum*, 183 in *A*. *gambiae* and 302 in *D*. *melanogaster*, data from vectorbase and flybase, Dec. 2015). This is mainly because of the lack of genomic data for *A*. *quadriimpressum*. However, compared with other coleopteran species in which the genome data are not available (91 olfactory genes in *L*. *decemlineata*, 52 in *M*. *alternatus*, 64 in *A*. *corpulenta* and 58 in *T*. *molitor*, a relatively high number of candidate active genes were identified in *A*. *quadriimpressum* and *L*. *decemlineata*, suggesting that olfactory detection might play a key role in superfamily Chrysomeloidea.

Motif analysis showed that motif 6 in coleopteran OBPs had high similarity (E-value 6.9e -4) with Andropin sequences in Drosophila mauritiana, and motif 7 showed similarity (E-vlaue 1.7e-1) with B-adaptin in Arabidopsis thaliana. These two proteins were previously identified as components in transmembrane signal transduction. [35, 36]. We still do not know if these two OBP motifs facilitate OR interactions. Future research into the functional role of the motifs should provide us a better understanding of how OBPs and ORs interact.

Combined with RT-PCR, RT-qPCR and homology analysis data, most candidate olfactory genes were shown to have male-specific expression patterns in *A*. *quadriimpressum*, suggesting that the olfactory system is highly developed in males and that olfactory detection plays a relatively important role in males. This result strongly supports the existence of a female-produced contact sex pheromone in *A*. *quadriimpressum* as previously shown on the molecular level [22], and additionally, a component of the female-produced sex pheromone and an aggregation pheromone have already been identified in other leaf beetles, such as *Leptinotarsa decemlineata* and *Phyllotreta cruciferae* [37, 38].

Notably, AquaOBP1/2/4/7/C1/C6, AquaCSP3/9, AquaOR8/9/10/14/15/18/20/ 26/29/33 and AquaIR8a/13/25a showed olfactory specific expression, suggesting that these candidate olfactory genes might play key roles in foraging and host-seeking in *A*. *quadriimpressum*. *A*. *quadriimpressum* is monophagous and a poor migrator that only feeds on the shoots and leaves of elms. Behavioral experiments demonstrated that *A*. *quadriimpressum* is attracted by elm volatiles and does not forage other plant leaves, even if starving to death [22, 23], indicating that *A*. *quadriimpressum* is highly reliant on olfaction. Functional characterization using of recombinantly expressed candidate CSPs/OBPs using volatiles from host and non-host plants is expected to lead to the development of more efficient, environmentally friendly control methods for A. quadriimpressum. Furthermore, AquaOR-AquaOrco expressing Xenopus oocytes can be established to aid in determining the narrow tuning of ORs to these chemicals. These results can provide valuable information on potential gene targets for genetic modification-based control strategies. A large number of similar studies were already performed and already put into use, especially in some well studied vector insects, such as mosquitos [39–41], but the precondition is the complete ORF sequences of these genes is available and accurate. Therefore, our results also provide necessary informations and narrowed the range of target genes to further functional expression studies.

AquaOBP4/C5, AquaCSP7/9/10, AquaOR15/17/20/24/32 and AquaIR4 were highly expressed in the antenna of males, suggesting that these genes are more likely related to sex-specific behaviors with a potential role as sex pheromone sensing protein/receptors. Many sex pheromones are already comercially used in monitoring and controlling pests, such as the use of sex pheromones in controlling some lepidopteran pests [42, 43]. The chemical constituents of *A*. *quadriimpressum* have not yet been finalized [22], therefore, further functional expression studies of these male sex-specific genes will help us to confirm the sex pheromone content of *A*. *quadriimpressum*, and then, aid in developing sex pheromone-based control methods. Homology analysis showed no orthologs were identified with PBPs from other scarab beetles such as *Popillia japonica*, *Exomala orientalis*, *Anomala cuprea* and *Brachysternus prasinus*, among others [44–46], which strongly indicated that sex pheromone sensing genes in leaf beetles is different from that of scarab beetles. As expected, AquaIR8a and AquaIR25a showed high expression levels, suggesting a co-receptor role for these two genes.

Based on RNA sequencing, a large number of candidate olfactory genes were identified in *A*. *quadriimpressum*. The transcriptomes of the antenna (olfactory organ) and the leg (non-olfactory organ) were compared. Moreover, RT-PCR, RT-qPCR and homology analysis were performed to confirm the tissue- and sex-specific expression patterns of these candidate chemosensory genes. Several potential functional olfactory genes were identified. Future studies aimed at exploring the *in vivo* functions of these genes and the subsequent development of environmentally friendly chemical control methods for *A*. *quadriimpressum* are the logical next steps.

## Supporting Information

S1 Table Nucleotide sequences of all identified candidate olfactory genes. (DOC)

S2 Table Primer used in RT-PCRs and RT-qPCRs. (DOC)
