# Supplementary material for: Identification and Comparison of Candidate Olfactory Genes in the Olfactory and Non-Olfactory Organs of Elm Pest Ambrostoma quadriimpressum (Coleoptera: Chrysomelidae) Based on Transcriptome Analysis
Source: PLoS One. 2016 Jan 22;11(1):e0147144. doi: 10.1371/journal.pone.0147144 (PMC4723088; doi:10.1371/journal.pone.0147144)
Supplement: S1 Table — (DOC) [file pone.0147144.s001.doc]

**Supplementary material 1**

**Nucleotide sequences of all identified candidate olfactory genes**

>AquaOBP 1

ATGTTCGCTTTGAATATTTTCGTGTTGTCAATACTAGCACATATTCTACTAGCAGCCGGTGCATCATCTGGTATTGATGATGAATGTGTAAAGATCGCCAATCTCACACTCGAAGATGTAGAAGGTTTTAGAATGGATGTGGAACCGAACCAAAATCAGTACTGCTTCTTTAAATGTATGATGGAGAAAATGGGTAGAATAAAACCTGATGGAACAGTAGATTTGGATGCTGTGAATAAATGTCCGAAGTTTTCTAAGTTTTCTGATAGTGACAAAAAGACAATTGAAAACTGTTTGGGACGTCTTGAAAATCTTGTGGAATGTGAGGATATGAAGAGATTCCGCAAATGCTTCGAAGGCTTCGGAGCACCCAACGAG

>AquaOBP C1

ATGAATTCTGCTTTGGTGATTATTGTTGTCTGCACCCTTCTGATTGAGGGAAAGTGCCAGAAACCAGATGGAGCATCAGAATCCATCAAGGCTTGTCTTTCGGAAACTGGTGTGCCTATGGAACTCATGCAGAATATGGAGCATAATGGTGAAGTAAGCGACGATCCAGCATTCAAAGAATATCTATTCTGTTTGGGGAAAAAGCAAGGCGTCATGACTGACTCTGGTGAGACAAATAAGGACATTTTCAAATCGATGGTACGCTACACGTTGGGAAAAAATGTCAAAGACGATGAATCAATTGAGAAGTGTTTAATCAAAATGGAGACTCCCCAAGAATCCGCCTTCCAAAGTATGAAGTGTGTCATGAAACTTCTAATGGAG

>AquaOBP 2

ATGATCGGTTGGAAAATATATTTTCTACAAATATTAGCATATATCCTTCTGGTAGGAGGAGAGAGTCCACCACCAGTATTCATAAAATGCATTGAAGAAACTAATGTCAATCCTGAAGAATTTTTTCCAATGAAAAAGGTTGCTGAGTTGGAAGAACCTATGCTCTGCTTTTTCAAATGTGTAATTGAGAAACTAGAGATCCTGAAATCGGATGGAACCCTTGATTTGAATTCATTGGAAAAATGCCCGGTCTTTACAAAGTTTAGTGACGATGATAAGAAAAAAGTGAAAACCTGTTTGGGAGGTATCGAGAAGATAAATGATTGTACAGATATGAAATATTATTTGGACTGCTTGATGATGACGATAAAGCCG

>AquaOBP C2

ATGAAGTGTTTTGTTCGGTTCGTTTATCTGTTCGCAATTGTGGCTCTGGTCACTGCACTAGACAAAGCTACGACCAGGGTAAAGAATGTCCACGAAGCCTGCCAAGCAGATCCTGCCACTCACGCTGACGAATACCTCATAGAAAAGGCCCGTTCGGAGCCTGTGGATCCGATTGTAGTCGGCCCTCACACTCTTTGCATGAACGTCCACGCAGGTCTGCAGAAAGAGAATGGAGATATCGACAAGGAAGACTTAAGAAGAGCTCTGAGCGAAGGAATACACGACGAAGCCAAAGTCGATGCCATTGTGAAGGACTGCGGAGTACGAGATGGTCGTACTCCTGAAGAAGCGTCTATTTCTCTCTTTAGGTGCATCTTTGGTCATGAGAACGCTTACGTGCACGAGTGGAAACCGCCTATGATAATACGCTCTTCTTCGACTGGTTCGGCGGCATCGTCTTTGGGCGTTTCCATGATAACGTTTTTTGTTTACTCAGTTTTATTTGTA

>AquaOBP 3

ATGAAGTTCTTCATTTTAGTTCTGTGCCTGGCACTGATAATGCACCATGCAGTCTCACAGATGAGTGAAAAGCAGATAAAGGCGACGAAAAAGTTAATTCGAAATACTTGCCAGAACAAATCCAAAGCGACAACTGAGGAACTCGATGCCATGCTTACTGGAAATTTCGATCAGAGCAAAAACGCACAGTGTTACCAGTTTTGCATATTGAACACTTACAAACTGCTCAAAAAAGATAACTCCTTCGACTGGGAAGCGGGAATCAACGCGCTCAAGGCGAATGCTCCCGAAAGAATAGCGGGACCCGGAAGCGTCAGCATCAAAAACTGCAAAGACGCTATAAAAACTACAAGCGACAAATGTAAGGGTTCGATGGAAATAGCCGAATGCATATACAAGGATAATCCTGACAATTACTTCTTACCT

>AquaOBP C3

ATGAAGTTCCCAGTTGTTTTCGTATGTGCAATTGCCGCAATTACTTTGGTTTGTGCGGACGAAAGCCCACAGCAAAGACTCGAGAGGACTCACAGTGAATGCCAATCCGATCCAGCCACAGTAGTGAAAGACTCCGTTCTCGAAGAAGCTGGCAAAGGCAATGTTGACGTGGAAGCCATCGGACCCCACACTCTCTGCATGAACGTCAAACTCGGGATGCAAAAACCAAACGGAGACATCGACAAGGACGAGCTGAGGCAAGCTGTCGGTAAACTGCCCGACGTCGATGCCGCCACCATCGATAAGATCGTTGTTGAATGTGGAAAACGTGATGGTGGCACTGCACATGAGGCTGCAGTTGCTTTATATGAGTGTTATGCAAAACTCACCAGCCAAAATCATAGCAAACAT

>AquaOBP 4

ATGAGAATCATGTGTAAGATGAATTGTACTATGATAATTCTAGTAACATTCATTATTCTCAATGTTTCTGGGATGAACGAGAAACAAATGGAGGCTGCACTGAAAATGGTAAAAAATGTATGTAAGCCGAAAACGAAAGCCACAGATGCGGACATTGACAAAATGCACAAAGGAGATTGGAATATCGATCACACAGCAATGTGCTTTATTTTTTGTGCCCTGAACATGTATAAATTGATGAATACTGATAACACCCTCAACTATGAATCCGCTTTGATACAACTGAAACAATTACCAGACAGCTTCAGAGAACCAACCAGGCAGTGTATGGAAAATTGCAAGGATGCCGCTGTCACGCTAGGAGACAAATGTATTGCAGCATATGAATTAGCGAAATGCATGTACATCTGCAATCCAGAGAAATTTTTTCTGCAT

>AquaOBP C4

ATGGTAATAATGGATGTCATTCGTAACGAAACACAACCAAATGTTCACAGCTGTCTTTTCAGGGGTTTCCTTCTTCACGGCGCATTTCTCCAAATATCCGTCGACCTTCGATTTATCGTGGGTCACCATGTCGATCCTGGTACGAATGAAAGGTTTGAGGAAATCGCCATTCTGAGTTTGCAGACCAGCTTTCACCGACATGCACAGCATGTGGATTCCTACTTGGGTATTTTCGGCATTGTCGCCAAGCTTCCTAAGCAGATCTTCGTCGCAGTAGGTCTTGGGATCCGCCTGACAGGCGAGATGGACCTGTTTCAGTTTTTCTTTCTCGTCCTCCGGAAGGTGATGAGCCATAGTAGCTACGACAACGCACATGAGAACCAACACAGCCTTCATCTTCAAGCCAAATTTCACGATCTCGTCACTGAAACTACTTTG

>AquaOBP 5

ATGACCAGTTCGGACATAATTCCAATTTGTTTTGCTATATTTCTTTGTGTGTTTTCCCAGCAAATAGTATCCTTTACAACGGAAGACCTCAATAACGATTTGAGATACATAAAGACCTGTAATAGAACTTCACCCATCAGTATGAGTACTATAAACGAGCTGCTGATAAACAAAAAACTAGTGAACGGCGAATCGAGCGCCTTCAAATGTTTCCTGCATTGCCTCTTCACCAAGTACGGTTGGATGGATGAAGAGGGCGGATTCCTTCTCCACGTCATCAAAGTGTCGTTAGAAGAAGCTGATGTAGAAATCGCAAGTTTGGAGTTCATTCTGTATATATGTACGGCTATAGAGTCGGCCGATAGCTGCGAAAGATCTTTCCTGTTCACGCAGTGTTTTTGGAATAAAATGGATGAGGTTGGCATCATGGTGAACTGCGATATAGCTACAAACTCCATCAAGCGAAAATCGGATTTGTACAATTTGAAATCTGCAGCGACCCCTTTTCAGCTGATCGAAACTTTGAGAAAGCAAGTTCTGGCCAAAGAGGATGAACTGGTGAAAGCCGAACAAATCAACAAGAACTTTGAGAGGATGATTCAACTGGTGAATATTCTAGGACAGGTGGATTCGTTTTTGACTGACCGAACCAAGACTATGATCAAGAAAATAGCCATGCTTGCGGACGCCGATGATGGAAAATACGAACAGGAATATTTCGGGCATAGTAAGAATAATCTGAAGAAGAAA

>AquaOBP C5

ATGGTCATTGTGGGATTTTCCTCATCATATGCATTCTTGGCAGACACGGATTATGGCGATGAGTTCCTTGCCCTGACCAAAAAATGGCACAACAAATGTATTGCTATTACAGGAGTGACCCAGGCCATGATAGATGAATTGAAAAACGGAACTTTTCTCGATAATGAAAAAGTCAAGAGGTACACCTTATGCCTGTGGTTGGTCTCGGAAGTGATGAACCCTGATTACACCCTGAATGAAGCATTATTGACGAAACTGATGCCAAAAAAAGTGGTCGAAGGAGTTGCAAGCTATTTGGCATGTGCGAAAAGTGCTAAAGAATCTGGTATTGCTGAGCCACACGAAAATATATGGGGACTGGTGAAATGTATTTACAACAGAGATCCTGCCACATTCATCATGTTT

>AquaOBP 6

ATGAGACAGTTCATTGTTTTCGCTCTGCTAATAGTAGCAGCAACAGCCAAATTGGATCGTAAACTGATTGGCGAATTGATGGGAATGGTGACTAAAGCAGCTGCAAAATGTGCAGATGAAGTGAAACCCAGCAGTGATGATATCTCTAAACTTCTGGAGCATAAAATACCGGACAGCCATGCGGGAAAATGCATGGTTTTGTGCGTGAACAAGGAACTCGGATTTGAAAAAGATGATGGTAGCGTCGATTTCGAGAATGGGAAGGTAATCATGGACAAAATCGAGCAAAGTGATCCCGAATTATTCCAAAAACTATACGACATATACAAAAAATGCGAAGCGTCTGATTACATGGATGCCGATGATCCATGTCAAACGAGTGCAAATTTAGCTGCCTGTGGTATCAAAGGGGCAGAAGAGGTGGGAATACCTTTCGACATATCGAGTATG

>AquaOBP C6

ATGAATGCAGCATACAAACTTATGATACTGGCTTTCATTGTTTACTCAGCAAAGGCCTTTCTGGAACGGAAGGATTATGGACCAATATTCACGAAAGTTGTTGATGAATGCGTCGACATATGCATAGATGTAACTGGCGCGCGTGACGAAGATATAAAACAAGTCGCAATTGGTAATTTTGTTGATGATGAAAAAGTGAAGAGATATATATATTGCCTTTGGAGGGTCTCAAGAGTGATGAACAGCAATCTGGAGGTGAATAGAACTTATTTGGATTATATTTTGCCAAAGAAAGAGGTGGAAGATAACTTCGATGATATGGAGAGCACTTGTCTTGCTGATGCCAAAAAAAAATATGGCAATGAAGAGCACTACGAAATAATCTATAAGTTTGAGGAATGCTTATTCAAGAAGAATCCAGAGGACTTCATTATGCCGGGAAAGTCAACA

>AquaOBP 7

ATGATAGTTCTCATTCATTTGGTTCTTCTCACACTAGTGCTTTCCTCTTCGGTTGGAGTCAGAATCAATAATGGAAACTTCGTCCAGAATTTGTCACCAAAGAAAACATTCGGGAGTTGTAAGACTGAGGCAGGAGCTACGATGGCTGACATAGAGGCGCTCAAAGCCAGAACAATTCCAAAAACGAAAACAGGACGTTGCTTCATGCAGTGCTTATTCAACAAAGCAAGAATAATGGACGACGGGAAATTTGATAAAAATGGAATGGTGGTCGCCTTCACACCTGCTCTCAAGGGCGATTTGACGAAGATCGGAAAGTTAAAAGAGTTATCGGAAGTATGTGAGAAAGAAATTGGTTTGGATAAACATGTGAACTGTGAGGGGGGCAAAAAAGTCGTCGATTGCATTGCAAAATATGGTAACTCATATGGACTGTCCTTAACTAACTCCCGAACGATG

>AquaOBP C7

ATGAGAAGAACTGTCTATTTAGTGTTATTGACAGTTGTCTTCGCTAAGGCCGCGCCTTCAATAGACCCATACGCAGACGACATGAAAGAAAGAGAAAAAATGGGTTTGGACTGTTTGAAAGACGTGAACATAGACAGACAGGTCATAGACAGAGCAGTGGCTACACTGAGCTTCCCAAGAGACGATGAGAAATACAAAGAATTTCTAGCATGCAGTTACAAAAAGCAAGGTTATCAAACAGATGACGGTGTCATTCAGTTTGACCACATCAAGGATTTTCTATCGAGGTTTTACAAAAGGAGCGATTTGAAGTTAATCGATAACTGCAGAAGCGATATCGAAAAAAAGTCATGGCGA

>AquaOBP 8

ATGAAACTGCAAGTGGCTTTGGTATTATTCGGTTTAGCTGTAGCGGCCTCGGCAGCAAAGGATTCTTCAGCTAAGTTTGAAGTGCGTACTCATGATGATGCCATAAAAGCTCACGAAGAATGCCGTGAAGAAAATAGTGTACCAGATGAAATATACGAGCAATTTTTGGAATACACTTTCCCAGACCATAAGCACACCAATTGCTATGTCAAATGCTTTGTCGAAAAAATGGGCCTCTTTACCGTAAGGAAGGGTTTCAACGAAGCCAACATCGTTTCTCAATTCGTTCAGGATAATCAAAGTTTTAGGAGCACCATCCAGCATGGCCTTGAAAAGTGTATTGACCACAACGAATGGGAATCGGATGTGTGCACCTGGGCCAATCGTGTGTTTTCATGCTGGTTGAAAATTAATCGTCATGTTGTACGCAAAAGTCTAGGCGATGGTAAGGATGAT

>AquaCSP 1

ATGGAAATCAAATATCAGAACTTTATCTCCATATTTCTCGGCGTGTGTGCTATTGCGACCATAGCAAGTTCTCTACCACAATCTTCTACAGATAGACCAAGTATTTCCGACGACGCTTTAGAGAGTACACTGCAAGATAAACGGTATCTACTGAGGCAATTGAAATGTGCTATAGGAGAAGCCCCCTGTGATCCCGTTGGGAGACGACTGAAAAGTTTAGCTCCGCTGGTTCTCCAAGGATCTTGTACACAATGCACTCCACAAGAGCAAAGACAAATCAGGAAGGTCCTGGGATATATGCAAGTTAACTTTCCCAAGGAGTGGAACAAAATACTGAAACAATACTCTGGA

>AquaCSP 2

ATGCCCTCAAGTCCGAGTGCAGCAAATGTAGCGACAAGCAACGTGAGAACTCCGACAAGGTACTGCGTTACATCATCGATAACAAGCCCGAGGAATGGAAGTACTTGCAAGACAAATACGATCCCGACAAGGTCTACTACAACAAATACAAGGGTGAAGCCGAAAAGCGCGGCATTAAAATCTAAATCGTTGAATGATTTGCAACAGCAGCAGAAGAAGAAGAGGCAGCAG

>AquaCSP 3

ATGAAACCAATTATGAGACGCGTCCTAGTGTCCTTCATGCTATGCCTGATTTGTGCTGCTGTGGTGGTTAACTGTGATGAAAAGAATATTAACAAATTGCTCAATAATCAGGTCATAGTGAGTCGTCAGATTATGTGTGTGCTGGAAAAGAGTCCTTGTGACCAATTGGGACGTCAGCTAAAAGCTGCTCTGCCTGAGGTCATTGTACGCAATTGTCGCAATTGTTCGCCGCAACAGGCGCAAAATGCTCAAAAACTTACAACATTTTTACAAACGAAATATCCTGACGTCTGGGCCATGCTCTTGAGGAAATATAAAACA

>AquaCSP 4

ATGAATATATCTGTAAGCATCTGCTCCTTACTCCTAGCAGTTTTCGTAAACTCTGACGAAAAATACACCACTAAATATGACAACATCGATTATGAGCAAATTCTACAAAGCGAGAGATTGTTGAAGAATTACGTCTACTGTCTTTTAGATAAAGGACCTTGCTCACCTGACGGAAAGGAACTGAAAAGTGTCCTTCATGAAGCCTTCGAAACCGACTGTGAAAAATGCAACGATCATCAAAAAATGGCATTGAGAAACATCATTCAGTTTCTCATAGAACACCATAAACATTGGTGGGAACAGTTAGCTGATAAATACGACCCGGATCATAGTTTCAAGCAGAAGTTCGAAAAAGAATTGGAGCACGAAAAAAATTCTTCTGGCCCT

>AquaCSP 5

ATGACTCGTTTGTTATTTTATTTCTTACTGCTCGGTGTTGTGGTAGTTGTGCTAGCACACGAAAAGAAAAATAGAGCGGATATACCAAAATATACAACAAGATACGATAATACTGACATCGATGCTATCATAAACAACGACAGACTTTTCATGAACTACATGTACTGTTGCATAGGCAAAGGAAAGTGCACTCCTGATGGTCTAGAACTGAAAAGTCATATACGGGATGCCTTGGAGCACGATTGTGACAAATGCAGCGAAACGCAAAAGAAGAGCATGAAGAAAATTGGTAAGAAATTGTACAAGGAGAAACCCGAATGGTGGAAGGAACTCTGTGACCATTTCGACCCTGACCATAAATACAGAACCAGATATGATTCATTCATCCAACAAGCTCTCGCTGAAAAAGATGAC

>AquaCSP 6

ATGAAAACCACCTTAGCCTGTTTATTGTTGGCCGCCATCTGTGTGGGTCTGTCCGCCGCCGACCAGAAATACACCAACAGATTCGATAATGTGGATGTGGACAGCGTCTTGGGTAACAACCGCATATTGAGCAATTACATCAAGTGCCTGATGGAAAAAGGACCTTGTACGCCCGAAGGACGTGAACTGAAAAAACTTTTACCCGATGCTTTGCAATCTGATTGCAGCAAATGCACCGACATCCAAAGGAAGAACTCCCAAAAGGTCATCACATTCTTGCGTGCCAATCGTCCCGGAGAATGGAAAATTCTCTTGGACAAATACGATCCTAATGGTGCCTACAGAGCCAGACATCACATT

>AquaCSP 7

ATGGTGCCGTTACTTCCTGTTTATGTCGCCGGCTTGTTTGGGTTAGCGGTGGCAGTTCCGATACAGCACTACGCCACCAAGTACGACCATATCGATATAGAGATGATCCTCAACAACCGGAGGATGGTCAACTACTACGCAGCGTGCTTGCTCAATAAAGGACCTTGCCCTCCGGAAGGACTGGAATTCAAGAGAATCCTGCCGGAAGCATTACGAACCAACTGCATGAAATGTACCGAGAAGCAGAAAACAGTGACAATGCGCACCATAAAACGTCTGAAGAAAGAATATCCGAAAGTATGGGCTCAGCTACAAAATGAATGGGACCCAGATGGCAGTTTCACAGCGAAGTTCGAGGAAACTTATGGTGATCGACAATCAGGACCTGTTCCCTCTGTATCATTGCAATTACTGAACAGGGTTGGTACCGATGACGGCTCATCAAAAGACGATGGCATAGCTATGAGTGAACCACCCAGCATATCAGCTCCAATAACTACCAAGGAAGTTGGTGAATCAACGAACAAATCGGAAAAGCCATCAACGAAAGACAATAGCATAGTGATGAAGGGAATAACTAGCACCATTGCCAGTACATCAATCCCAACAACCACTAAAAAAGCCATGGTATCTACAAAAAAATTGGAAAACAAAAGTGTGAAACCCAGTCTAGTTACAAAAAAGGTCACCACTTCGACTGAATCAGCAACGATAGTCAATAAAATCATTACTAATGCTCCCAGTGTAACCAGCACAATCACCAGTAATTATTACGCTCCTCCAGTTCGTTTTAGACCACTGGCTAACATTGGGGCTGGAATAGAAGCCACGGTAAGTTTGGGAACTGACATTGTCGGAAATCTAGTGAGGGGTATTGGTGCCATAGGAGACAGATTGGTACAGACTGGTGCTGAGATTGCAGGAGTTGTACTCAAAAGTATCACAAGGCCCCTT

>AquaCSP 8

ATGATCCATTTTTTGATGATATTATCCTGTTTGACCACAGCAGTCTTGTCAGCAGTACCTGAAAAGACGAAGTATACTACAAAATACGACAACGTAGACCTGGAAGAGATCATTAAAAATGACAGACTCTTGAAAAACTACGTGAACTGTCTCTTGGAGAAAGGAAAGTGTACACCAGATGGATTGGAACTCAAGAAAAATATGCCAGATGCTATAGAGACAGACTGCAGCAAATGCAGCGAGAAGCAGAAGGAAGGGTCTGAAATTATCATGAGATATCTGATCGACAATAAACCTGACTATTGGAATCCTTTGCAGGAGAAGTACGATCCTTCCGGAAGCTACAAGAAGAGGTATTTGGATACCAAGAAGACAGAAGTCAACGTTGAACCTATTGTGAAATCT

>AquaCSP 9

ATGAAAAGTGTGAGTGCATTGATTCTGTTCTGCTTTTTGGCAGTTATCGCTGCACAGAATACATATAATGGCAAATATGATAATATTGATGTTGACAAAATCTTGAAAAATGAGAGAGTGTTGGCTAATTATGTCAAGTGCTTGATGGAAGAAGGACCGTGTACACCAGAGGGAAGGGAACTGAAGAAAACCCTTCCCGATGCTCTGAAGAACGGATGTGATAAATGCAACCCAAATCAGAGAGGTACAGCAGAAAAAGTCATGAAACATCTCATGACGAAGAGAGCCAGGGACTGGGACAGACTCACGAAGAAATATGACCCTCAAGGACACTACAAGAAACGCTATCAAGAACAATTGGAGAAGGCTGCGAAGTCTCAAGAA

>AquaCSP 10

ATGGGTTTCACCCACTTGGTGTGTTTGTTCGTCATAGTTACCGTTGCCCTTTGTGACACCTATAACACAAAATACGACCATGTAGACGTTGATTCCATTCTAGCAAATAAGCGGGTCCTAGCGAGTTATATCAGATGCATCTTGGACGAAGGGCCTTGTACACCAGACGGCAGGGAGTTCAGAAAACATATACCTGAAGCTATAACAAACAACTGCGCAAAATGCTCGGATCCACAAAAGAAAATCATCAGGAAGACATCAAGATTCATCGAGAGAGAACGACCACAAGACTGGAACAAAATAAGCAAAAAATTCGATCCTCAACAAAAATTCACTGCAAGTTTCAGACAATTCCTAAATGAAAAT

>AquaOrco

ATGATGAAATTCAAAGTATCCGGCCTTGTTGCTGACCTTATGCCCAACATAAGGCTCATACAAGCATCGGGGCATTTTATGTTCAATTATCATGCAGACAATTCAGGAGCACTGCATGCATTGCGACTGGGATATTCTTGCCTGCATCTAGTGCTCTGTTTGGTGCAATTCGGATGTACCTTCGGTAATTTAGTAATAGAACGAAACGATGTGAATGATTTAGCAGCGAATACTATCACAGTATTATTCTTCACCCACTGTATAACGAAGTTCGTCTACTTTGCAGTTAGATCAAAACTCTTCTATCGGACATTAGGAATATGGAATAAGGCGAATAGTCATCCTCTATTTCTTGAATCGAACAATAGGTATCATGCCCTATCACTGAAGAAAATGAGGACCCTATTGATATGTGTGATGACAACAACGATTTTGTCAGCATCAGCCTGGACCGCCATCACTTTTGTTGGAGACAGCGTACACAATGTGAAAGATCCCGATAACGACAATGAAACCATCACTGAGGAAATACCCAGACTCTTAATAAAATCTTGGTACCCCTGGAATGCCATGTCTGGAACAGCCTACTATGTTTCAGTGTCTTTTCAGATCTATTACGTTTTCTTTTCATTGGCTCATTCAAATCTCATGGACAGCTTGTTCTGTTCATGGTTGATTTTTGCGTGTGAACAGCTCCAACATTTGAAAGAGATCATGAAACCGTTGATGGAACTGTCGGCATCTTTAGATACGTATGTTCCAAAGAGTGCAGACCTATTCAGAGCACCTAGTGCAAATTCTCAAGATAATTTGATAGAAAATGAATATAATGAAAAGAATGAGGGGCTCAACCTGAAAGGGGTCTACAATACAAGGCAAGAGATGGGTGCGAATTTCCGGAGTGGAGCTCTTCAGACATTTGGACAAGGTGGAGGAGGAGTTGGACCAAATGGATTGAGTAAAAAGCAAGAACTCATGGTGAGATCAGCTATCAAATACTGGGTGGAAAGACATAAGCATGTAGTGAGGCTTGTAACTGCAATTGGAGATGCATATGGTGTGGCCCTCCTGCTACATATGTTAACAGCAACTGTAATGCTGACCCTTCTGGCATATCAAGCGACTAAGATCGATGGGGTCAATAAATACGCTGCTACTGTGATAGGATACTTAGTTTATTCTCTGGCTCAAGTATTCCATTTCTGCATTTTTGGAAACAGGCTCATCGAGGAGAGTTCCTCAGTCATGGAAGCAGCTTATAGCTGCCATTGGTATGATGGTTCAGAGGAAGCAAAAACTTTCGTCCAGATTGTATGTCAACAATGTCAAAAGGCGTTATCGATTTCGGGAGCTAAGTTTTTCACGATTTCTTTGGATCTCTTCGCGTCGGTACTCGGAGCTGTGGTTACCTATTTCATGGTGCTGGTGCAACTCAAG

>AquaOR 1

ATGGATGACTTGAAGAACTTCACGGCTTGTGGATATGTGATTGCCATAGCTTGTATGGCCAACGTAAGATCCTTTTATTTCCTGAAAAACCGAAAAGAGCTCCTTCACCTCATCGACTCCCTGGACGACGCACAATTCTCACCAGTGAATAAAGAACAATATCGGATGGCGAGGAAATCACTACTGTTTTATCAAACAGTCAAAAGATTTGCTCGCAACACAGAAATACTCTTCAACAGAATATATCTCGGACAGTTCATTGCGTGTACTTCAGCTCTATGTATGGCGTTATTTTTGCTCACTCTGCAACAAGAAAGCAATTTTGAGTGTCTGTTCTTGGTTTTCTACCTAGCATCGATTTTTTCACTTCTTCTGATCCCCTGTTGGTTCTCATCGGAGATGTGTAGGAAGAGTGAGAATATTGCGAATGCAGCCTACAGTTGTCATTGGATTACAGCTTCTAAAACTTTCAAGAAAGATCTGACATTCTTTATACATAGATCTCAGACCCCCATACGATTCTATGCTGTGGGAATTTTCCATATTTCCGTAGAAACATTCATGTCGATTGTGCGGTCTTCTTTTTCTTTCTATACTGTATTGAACAATCTAGCACTGGAAGAAGAA

>AquaOR 2

ATGGATGCTAGTTTTTCTATCCTGGGATGTTACCTGGCTTCTTACGATACCACATTCACTTCGATTTTAATTTGTCTCAAAGTTAAGATTCAGATTTTGAACGAGGCAATCAAGAGTATCAGAGAAAGAGCTTTATCAAAACTGAACCAACATAGCGAGTCTGACTTGGAGCTAGTTGATCCGAAACTGGAAAAAATATTGTATGGAAAAATCGTACACTGTGCCAAACATCTGGACTCCCTCTTGAGTGTTTGTGTTGAAACGGAAAATATTTTCAAATATGTCACTCTGCTGCAAATGATAGACTCCCTTCTCGTCATGGCATCATGCCTCTTCGTGGCATCTCTCATATCCCAATCTGATCCTGATTTCATCGCAATGGCCCAGTATATGATTTCCGTACTAACTCAACTACTGACGATATGCTATTTCGGAAATGAGATTACTGAAGTGAGTTCCACTCTGAATTCTTCTCTGTATCAGAGCAACTGGCTGAACTGTAGCAAGCGGTACAAGCAGTGCATACTGATAATGATGTGTCGTATGCAGAAGAAACTTTGCATGACCATTGGAAACTTCTCACCTCTGACTTTGAATACATTTCTTGCTGTTGTCAAGGGATCGTTCTCATATTGTGCAGTATTCCAACGAGTCAACAATGAG

>AquaOR 3

ATGTTCAAAGAATCTATCAAGAAGATCAGCACTTTTGCCAGCCATATCGGAATGGTAATCACGCATTTTGCGGGAATCGTGAAATTGTGTTTACTTACTTTCGGCCACGGGAAAATACTGAGAATCATGCAAGTTCTACAGGATAAAAAATATCGCTATGAGTCTTTGGGAGAATCGAGGCCAGGCTATATGATGCAGAAAGAAAAGAAAGTTAATAATGTAACAACTTATTCGACGATCATCTTGTACACTCTTGTTGGCATCTCCGGACATATATCCTCCCTGATCAACTTGAATAGAGAGATCGAAGGGGATTCTTTCGAAGGAACAAACAAGACTTGCTACGATTTTTTGCCTTACATGTTCTATATACCTCTGCCCTCTTCGAAGAAGTGGCAATGTAAACTGGTTCTCTCTTTCATGGATATTGGTTTTGCTATCAGTGCCTTCGTGATTGCAGCACACGATGGAATATTTACTGGCCTACTCAATCAACTGAAAACCCAACTGCTCATAGTTTGTGACGTTTTTAAAACGATACGTTCGCGAAGTTTGAAGAATACTGGCTTATCTGAGGATTACCTAATCACCCGTGATGTTGATAACCCTGAACTCGAAAATGAATTATACAAATTGCTTACCCATGCCACTGAACACCTAAACATATTATTGGGGGTGAGGAATGATCTAGAGTTCATTTTCACGTACGTCATTTTGATTCAAGCTCTTGCCTCGTTGTTCATCTTGTCGTCATGTTTGTACGTTGCGTCCACTGTACCGATTGGATCTCCTGAAATGATTGCTCAGTTTGAATACTTCTTTTGCATTTTCCTACAACTAGCACTCATATGTTGGTTTGGAAACGAAATCACAAGAGCGAGCGAATTGATCAGATTTTCCTTGTTCCAAAGTGATTGGTTGAGCTCCAGTCCCCGTTTCAAGCAAGCCATGATACTGACCTTCATTCGAATGCAACGCCCTGTGTACTTGTCTATCGGAAAATTCAGCCCTCTCACATTGGCCACCCTTGTTGCAGTATGTCGCGGCTCATTTTCTTAC

>AquaOR 4

ATGTCTGATTCAGAGAACAATCTAGGTTATCCAAGTAATTTTTTTCACACAAATGAGGTGATTAGAAAAATTTCTGGTATTTGGCTCCCCGGTAGCGAATATCATTTAGCGCTAAGGGGATTGTATTTTCTCTATGTATCATTTCTATATGGTACCGGCCTGGCTTTGTTCATTTGTGAATTTTTGATTTTCCATGAAACCATAACAGGAATCAGTAAATTTGTCAGTCATATTGGAATGTTGTTCACTCATGTCGTTGGAATACTGAAGATGTCCATTTTGGTTTTTGGAAGGAAGAAATTACAGAAAATCATGGATGTTTTACAAGACAAAAAGTACTTTTACTCTCCTTCAGGTGATTCCCATCCTGGTTCGTTAGTTGTTGGAGAAAAATTCATCAGTTCAGGATTTTCCATATTGGTATTCGTCTTGTATACTTTCGTTGGAGTATCTGCACACATATCATCGCTGATAACCATAAATAAGGAAGTGAAAGGAGACAGTTTCAGTGAAACGAATAAGACTTGTTACGATTACATGCCATACTACCTTCATATCCCTTTTTCGACAGAGACGAAAGGACAATGTGGGGTTGCCTTTGCTTTCATGGATGTTGGACTTGGAATTTTCGCTTGGGTTATTGCTTGTCACGATGGAGTGTTTTTTGGCCTCCTGAATTGCCTTAAAACCCAACTACTGATAGTTTGCAATGTTTTTAAGACCATTCGCGTGAGAAGTTTGGAAGCTGTGAATCTGCCCAAAAATTACACGGTTCTGCAAGATACACATAACCCACTTCTTGAACTAGAGCTGTACAGACAACTGACTCACTGTACCGAACATCTTAGAATATTATTGAAGGTGAGAGATGACCTCGAAAATATTTTCACATTTGTGACACTGAGCCAAACCTTGGCATCATTACTGATTTTTGCATCTTGTCTATATGTTGCATCAACAGTTCCTATGACATCTCCTGAATTCTTTGCCCAGATGGAATATTTTATATGCGTCCTTGTGCAGTTCTCTCTGATTTGTTGGTTTGGTAACGAAATAACAAGTGCAAGTGAGCTGATCAAATTATCCCTATATGAAAGTGACTGGTTGAGCTCAAGTCCGCGTTTCAAAAGCTCTATGATTCTAACCATGATTCGCATGCAGCGTCCTGTCTATCTATCTATTGGAAAATTCACACCACTTACTCTCACTACACTCGTTGCAGTTTGCCGAGGATCTTTCTCATATTTTGCACTGTTCAAAAGTGTACAG

>AquaOR 5

ATGCATTTGGAAATCATTCAAGGAGCATTCGTTTCAATCAGAGAAAGAGCCGTTGAAAAATGTACTGGTCCTTCTGTAACACCAGATGGCCTTCACAATTCGCAAGAGTTGAAGACGATATTAAACCAGGAAATGAAAAAAGTTTGCAGACATCTTCAAACCGTTTACAAGGTCTGCGAAGAATTGGAAAATATACACACGTTTCTCACCCTGGCTCAGACTGTAGCAACCTTGTTCATACTTTGTTCATGCTTGTACCTGGTTTCTTCGACTCCCGCCAGTAGTAAGCAGTTTTTGGCGGAGCTAGTGTATATGACAGCTATGTGTTTTCAACTGATACTGTATTGTTGGTTTGGAAATGAAGTTACTCTGAAGGCAGACAAAATGTCCTTTTACATATGGCAATGCGACTGGATCACAGCAGACCCAGAATTCAAAAGAGCGATGGTTTTTACTATGGCTAGGGCAAAACGACCTCTTTATCTCTCGGCTGGAAAGTTTGCTCCTCTCAAGTTGGACACTTTTTTAGCAATTATCAAAGCGTCCTACTCATTTTATGCGGTAATAAAAACCACAAGTGAT

>AquaOR 6

ATGGAGGCACATGATATAAACTTTCGAAATATCATAATTGTGAATTTCAATTTCCTTTATTTCTTTGGCATCATGTATCCAGAATTTGATACTTTTGGTTCGGTGGTTATCTACATTATCAGAATCCTTATACTTTTGGGTTTCTTTTTTGCTGGGATCGTCACATGTGAGGTGATCAACTGGTACTTCAGCTTGGGTGACTTGGAAGCGACGGTAAACGCCTCGTTTCTAACCTTATCAAACATAGTTTCGATTGCAAAATTCTATGTCATCGCCAGACATCAAGAGAAAATACTCAAACTAGCGGAACTGATAAACAGAAAAGAATTCAAACCAAAATCCGAAGAGCAACGTCTGATCTTGAAAAATTACATCAAAACGTCGAAGGTCATTTCAGGACTGACGTATTGTGGATGCGTTTTCACTTGTGCCTTCTGGGCGATCTACCCCTTCACAGAAGATGGTGATGCTTTCCTTCCGATAGCGGCTTGGGTTCCATTTCGAACCGATTCGTCACCATATTTTGAAATCGCTTTCGTTTATGAAATAATTGCTACAGTCATTGGTGGTCTCACTGATTTGAGTGCTGACTGCCTGATTGCAGGTTTCATCATGGTTATCTGTGCTCAGTTGAAAATCCTCAATGATTCCTTAAGTAACATAAGGAAATTTTCCCTGGAGGAAATGAAAATTGAATTAGGCGAAAACGAAGACAAGATATCACCGAAACTTCAGGATACCATGAACAAAAAATTACTGGAATGTGTAATTCATCACAGATATATTTTGGAGTTTGCTGGGGAAGTTACCTTTTTGTTTACGACAAGTATACTGGGCCAATTTGCGGTGAGCGCAATAATCATTTGTACAACGTTATTCGAAATGACTTTGGTACCTTTCACTAGTGTGAAGTTTCTTTCTCTTATACTTTATCAATACTGCATGCTCATGGAAATCTTCATCGTGTGTTATTTTGGAAACGAAGTCATATTAGAGAGTTCGAAACTCACAAACTTCGCTTATCACAGCGATTGGCAGGACTGTTCGCAAGAATTCAAACGTAACCTGATTTTCTTCATGACACGATCACAGAGG

>AquaOR 7

ATGAAACCGCCAAGACGTTCGTATTTGGCTTTTACAATAGAAATTCTGGAGTTGGTCAACATGTGGACGGAGAAGAGAGGTTTTCTGAGTTTACTGAGGGAATACTACGCTATGATCATCATCGTGGGCTCTACTGTGGCGATAATGACTGACTTTTTTCTACAATTTTACGATGAGCAGTCTCATTTCACGAGCCTCATAGAAAGTCTCATCGGAGGGAGTGCTCTCTGCAGCGTCATTTACGTGTCGATATGTTTCCTTTTGAAAAAAGAGCAAATAAAACGTCTGGTTGCCAGTTTGGATATCTTCGAAGAGTACCTTCCCGAGAAAGGCATAGAGGAGGCGGAAGAATCGGCGAGGTTCTACACGAAATCCTTCCTCTTTTACGGAATCGTGGGCAACGGGCTCTATGAAGCGTCTCCGTTCATGTCGTTCAGGGAGTGCAATGAAGAAAGAACCGAAAATATGATAAAGATGGGGATACCCTGCAAAGTGATTGTGAGATATGTTCTTCCTTTCAAATATGACACCTCGCCGTTTTATGAGTTGGTGATTTTGGAACAAGTCACAGTAGCCATTTTAGGTACTATTGTTGTGATGACCATTTCGATGCTGGTCTGTGGTATCTTAACACATATTGCTGCAAATCTGAAACATCTCAAGAAAATGATAAAGTTCATATCCCAGATCGAGGAACATAAATTGAAAGAGCACGTCAATTTGTGTATCAAATACCACACCGTTATTCTAGAAGTTTCAGATAAAACCAACGAGGCTTTCAGTGGCATGATGTTAATCCATATAACTTGGACCAGTTTCATAATAAGTGTATTAGGCTTTGGAATC

>AquaOR 8

ATGTCGATTCAGGGAGAGAGCTCGGTGTCACCTGATTTGTATAAAATTGGGAAGAATAAGCCTTTTTCTGCGACCCTTGATACCCTGACTAGCTGTTTTATGTATCCAAAGAATCTTCAAAAATGTTACATGGTACGATTCTACACGTTTGCTGCTATTTTAATAGTAGCGTCATTTGTGTTTTGCTGTTTCTTGAGTTTGTTGCATTTGATTATGGCCAAAGTAGATGGCAAAAACACAGACATCAGCGAAGACGTGTCTCTCATTACAGCAGGCACCGGTCTCGTCTTCGCAAACGCGATGTTCTGTTACAGATGCAGAGGCTGGTCCGCGGTCATGAGCAGAATCGCACGCTTGTGTCAGCTAGAGGATTCCCCGAAGACAGAAACTACCATAAAAAGATGCAATAAGCTTTCTAAGATGTTCACAGGTTACTGTTTTCTTAGTACAATCATCTACGGAACTGCGGCGTACTACGAATCCTTCAACTGCGTTAAAATCAACGAGGAGAAAGGTCTCGATGAGATATGTCGTACTTTCATGACAGTTTGGCTACCATTCAAATTGAGCGTTTTGGGAGAATTCGTACTATTTGGTCTACAAGCAGCCTTTGCTATCGCGGTAGTTGTCCCTGCTGTTACGGTTTTCTTCGTTAACTGGGAAAGTGCAGAACTGATTTCTTTGTGCTGCGATCATCTGAAGGACTCGTCGCTATCAATACTGGAAGAAATGAATATCGATAGAAGGCATGAGAAGTTGAAACGGTGGATATTTCATCACCAACAGATTTTAGGGGTTATTGCCCAGTTCAATAAAGAAATGAGATTCAGCGTGGGACATTTATCTTTAATTGCTGCCCTGGTTTTTGCCTGTCTCATTAACCAAGCTACTAACTACAAAGTCTGTGGGGCTCTCTTCGAGCTTCTTGGCTGGCTAATTTCTCTATTTTTGATTTGTGACGCTGGTCAGAAGATATCTGATAGTACCCTTTCGATAAGTGAAGCAATATACGAGATGGAATGGTACTCTACCGATACCCAGACCATGAAATATCTTTTGGTCATATTGATGCGATCTCAGAGGCCGATGGTGTTGGATGCTCTGCCTCTAGGAACCTTGAATTACACATTGTATTTGATGATGTTGAAGGCATCATATTCATTCTGGACACTTCTCTCACACACCACC

>AquaOR 9

ATGGCCAAAATAATCAATTACACCAAATTTTTCTCAATCAATATGTTCGTATTCAGAGTGTTGGGCTTTTGGACACCTGATCATAATATGCGTTATAAGCAGTTATACAATTTATACACGATTCTGTGCACAATATCATGGTTTCTGTTCCTGTCATCCCAAGCTACATATCTGATTACTTCCCTAAATGGTATTGGAGAATTGACCATTGTATTTTTCACAGCAGTGACGTTTGGCGCCAATTTCATAAAGACGATGGCAATTTATAGAAAAAACGATGTGATCAAATCATGGATGAAGAACCTCCACCAACCACTGTTGCAACCAAAGTGCAAAAAACATCACAAAATGGCACAATCCACCGAACGGTTTCATATTAAATTATTCTACATATGTTTGTATTTGGGTGTACAAACATACCTCTTTTTCTCTGCATTACCATTTCTGCAAGAAGAAACTATAATGCTAAGTCAGGGATGGTTTCCTTTTGACTGGAGAAAATCCCCAAATTATGAGATCATTTATATATTCCAAAACTCTGTTACTTTATGGAATACCATCATGTGCCTGAACTTGGACACATTTTCTGCGGGTTTGCTGTCACAAATTGGTTTACAATGCGATTATTTGATTGTAACATTGAGTTCTTTGGATGAATTTTCGATTGAAAATGGATCATTGCGGGAAAGTGACGAGTCTAGTATTCATTTAAGTCGTAGGGATCCCGAAATTTTTTCGAGTAAGATGACTCATAACTTGATAATATGCATCGAACATTACCAAAAAATCAGAAGATTGTCAAAGGAAATCGAAAAAATTCACGAAACAAGTATTTTCTTTCTTTTTTCTGGAGGTGGGCTCATAATATGTTCCGGCTTGTTTCAGTTGACAGTGGTACGAATCGGGAGTATTCAGTTCCTTATGGTGGTCTCTTTTCTGATGTCTATGCTCACGGAACAATTTTTGTACTGCTGGTTCGGAAACGAGATTATTTACAAGAGCACCGAAATATCTAATGCCGCTTATAACACGCCGTGGCTTGATTGTGACATACGATACAAGAAAATTCTGCTCAATTTTATGATTCAAACCAAAAACCCTATAGAAATTATGGTGGGAGGTTTATTTTCGATGTCAATTCATGCCTTTAAATCGGTTGTTCAATCTTCATATTCTGGCTTCGCTCTACTGAAGAAATTACAAGATAAGAGAGCG

>AquaOR 10

ATGCAGGAATATGTCGACTTCACGAAATACTTTTCTTTCCATATATCCACCTTCAAACTCCTGGGATTCTGGAAACCCAACGAAGACTTACGACATAAAACATTATACAACGTCTACACTTTTTTCTGCACAGCAATATGGCTATCATTCGTTCTGTCCCAGCTTATATATGTCTTCACATCTTTTACCAATACGAAGGAAATGGCAGCAATTCTTTATGTTGCTGGCACTGTGTCATTAGACCTCATCAAGATGTTGGCAATATACAGCAATATGGACCTCATCAAGCATTTACTGAAAGAACTCAACAACCAGTTTTTTCAACCTAAATGCAAGGAACACTTGGATCTGGCAAGGAATGTGAAGAAATTCCATACGAGTTTATTTTATTTCTGTCTTTATTTTGGCCTGCAAACATACTTATGCTTCTCCTTCGTTCCTTTCCTCTATGACGAGACCGTTACGTTAACACCAGGCTGGTTTCCAATTGACTGGTCAAAGCCGTTCAACTATGGAATTGTCTACGCTTATCAAAATATCGTTATACTGTGGAACGCTCTGATATTTTTGAATTTAGATACGTTTTCTTCTGGACTCCTCATGCAGGTTGGCTTGCAGTGCGACTTTTTGAGTATAACATTGAGTAATATAGAGAATTTCCATGTTTGTGATGGAGTTCTGCATGAGAACGATGTCTCGGTTTTCGATTGGTCGGAATATAGTCCTGGAGAGTTCAGTGACCAAATGCTGGAAAACTTGATGATATGCATTGAACACTACCAGAAAATCAAAACGTTATCTAAAGAAATTGAAGATATTCATCACACGAGCGTATTTCTCCTTTTCTTGGGTGGAGCTATGATTATATGTGCCGGTCTATTTCAACTGAGTTTGGTGAGCATTTCAATCCATATGATAATTCGAATGGCCGTTCCCAGATTTCACAATCTCTTTGGACTGAATTTATTAAACATTCAATCA

>AquaOR 11

ATGCCTCGAATGATTCTGCGAAAAGTTCGCTGTCGTAACACAAAAGAACCGATGTTTCTACCTTTCGATACTTCCCTGGATGGGTACTACCGACTGGAATACTTTTTTAGTTTCTATACGCATCTTGGAAATGTCCTGCTATTTTTGCCCATAGAAACCACCCTCACATGTTCCATCATACATCTCATCAGCCAAACCGCTATATTGAAGGAAGCTTTCGGTTACATTGACAAAGGCGTATCAGACGAGCAGGAGTCGAAGAGTATCGCCATTATTAAAGAATTCAGAATCGTCAAATGCATCAACGAATTGCAGCAAATCTACAGGGCCGTACAGAAATTAGAAGATTTCTGTAATATTCAGCTCATGATTCAGTATGGATTTGCCACATTCCTGTTGTGTACCATCTGCCACGTTATTCCACTGATGAACAATATGGTGGAAGGTGTGAGCAACCTAATTTTCGTCTCCTTATCTCTGGGTCAGATTTTCGTTTTCTCGTACTGCTGTCAAACATTATGCTTGGATTTACAGGGTATCTGTATACCGATATACAATCTTCAGTGGATTGATTATCCCCTGAAAGTACGGCGTAGCTTGGCATTTCTGATCAGGAGATTACAAAAGCCCGCCAATTTGACCGCTGGGAAGATGGTTATCATTGACTTGCTGTTTTTCATACAGGTTATTCAGAAGTCATACTCGTTTTATACACTGATCACCAATACTAACAAGCGAAAA

>AquaOR 12

ATGTTGAATCCTATCTTCTTGATTCACTCTGGAGCGAGTCTTTTCAATATGTGTTGCCTCTCATATACGGCAACGAAGGCCGAATCGTTTGCTATGCCGCATTTGATGATGATGACTTTGGTCGTGCTAATCGAAGTGTTTACCAGCTGTTGGTTTTGTAACGACTTCACGTTGGAGTTTTCTAACACATTGAGAGCTGTTTACGACTTGGACTGGTTGGGATATCCGCCAAAATTGAGAAGAACAATAGTTTTTGCTTTGGCGAGACTTCAAAAACCTCCGTCTTTCACGCTGGGAAAGTGGATTAACATTGACATGCTTGCCTTCTTCAATCTGCTGAAAATGGTTTACTCATTTTACATGCTTATATCGAAGGTGAATAAG

>AquaOR 13

ATGTTGGATGTTTCTCTTGGAAGCACCCAGTTCATGAGTTTATTCGTCTATATGAATGCCATGTTCATGCAAGTTTTATTATATTGTTTCTTCGGACATAGTATCATGACAAATAGTGAAGATGTAAACTTAGCCATGTACATGTCAAACTGGCATAAATCCGAGAAAAGAGTTCGCAGAACATTTCTCATCTTTATGGAAAGATGTAAGGTACCAATTATTTTGACAATCGCCAAAATTTTTCCATTGAATTTAGTAACGCTCAGCTGGATTATACGATCATCGTATTCATTTTTAGCGGTTTTACGAACCATATAT

>AquaOR 14

ATGTCTTGTTTTATAACTCTTTTACTCTTCAGTTCGATGCCAATTTTCACCCAGAGGGATTTACCAATGCCGTTTTCATTTGACATAGGAAAGTTCAAACCGGCTATGTATGTCTTTCAAATAATGGGAATGCTCACCACATCAGTAAATAATTCTAGTTTGGACTTACTAGCAATAAGTTCCATGGGAATTTGTGCAGCTCAAATCCAAATACTCAACAGGAAAATTATTGGTTTGAGTCAATGGAAAGAAAATGAAGGGAATACACTACACAACGTCAATTCTGACCTTTGCAAATGTGTGAAACATCACGTTGAAATAATAAGGTTCAACGGAATTGTGGAAAAAGTATTTTCTTATATATCATTCGCTCAGTATGCTGCAAGTGCTATCACAATATGCAATACAGGATTTCAATTAGTACACGTTCAGCCTACCAGTTTGAAGTTCATGATAATGGTGTTCTTCTTAACTGTATTGATGATTCAACTCGTAATGTACTGCTGGTGTGGAGATGAAATTATAGTGAAGAGTTTGGATACAACCAATGCATGCTATAATTTCAAATGGTATGAATCAGACCTCAAAACAAGGAAAACTTTGATTATCATAATGGAGCGCAGTAAAAGACCCTTGTTCCTTACAGCAGGGAAACTTTCGATACTGTCTTTACAATCTTTCACTTCGGTGATAAGGACGTCTTACTCGTATTTCACAGTCATGCAAACACTGTATACAGATCAGAAC

>AquaOR 15

ATGAAAATTGTTGGAATATACCCATACGATAACTGGCCCATGATGTACAAAATCTACGCCCAATTCTCCTACATTTTCTTCACCGCACCAACACCAATTCTTGCAGCGATGTCTTTCATAGTCTCCACAGAAAAAGACATGGAAAAAATTTGCGATAATGCCTTTCTAGTCGCACAGCTAGGCATTCTGCTGGTGAAGCTATGGCCTTTCAAAAATAACCCAGAAGCAGTAAAACGGACCGTGAATGGTTTGAACCGAGAAATTTTCAATTCCTATAGACCGGACCAGGAATATATCATACGAGGCGTTATCAGGGAGTATAATTTTATATTTTTTGGGACAGCTTTTGCGAGTTTAGCTAGTTTATTCACCTGGTTTGGAAAGGCCTTCTTCTACGAAAACAGGAGATTCCCACTCGACATCTGGCTGCCTTTTGAGCCATTTGAAGATACTGTGGTGTATGTGGCGATATTGGTTTACTTATTTTTCAGTGTACTTAGTGGAGCTTTGGATAACATTTCCTTAGATACCCTAATCGTGGGAATGATATTTCAGTCAGCAGCACAAGTAAAGATACTAAAAAATAACCTACAGTTACTCAACGAAAGAGTTGAACAGGACGTTAACTCTAATTATAATTCGAATGGTCCACCCAGGAATGATTTCAAAGCAAAACTTGTCTACGAAAACATTTGCAAGTGTATCGATCACTACGACGCAATTTATGAGTATACCAAAGAAGTAGAAACTGTATATTCATTGGTGGTTTTCACTCAACTATTTGCCAGTATCGTGGTGATATGTATTTCATGTCTGCAACTGTCTATAGTTCAACCATTCTCTTTCTCATTCTTTGGTATGGTTACGTATATAATAACAATGTTGATACAACTATTTCTGTATTGCTATTATGGTGCAATACTCTATGAAGAGAGCAATACTTTGTCCACCGCTATATATGGGAGTGAATGGTACAACTACGATCAGAAATCTAAGAAATGTTTGGTAATATTGATGGAAAGAGCAAAGAGACCTCTCAAACAAACCGCTGGAAAATTCTTTGACCTTTCTCTTGACACCTTCACAATGATTTTGAGACGGTCCTATTCCTTGCTGGCCGTAATGAAGAATTAT

>AquaOR 16

ATGGGAAGTGGAGACAAAGATGTTATTGAAATTTATGAATCTCACACTGCGCAGAGCAAGATATCCAATGTCATCTTTCTAGTGAACATATTCATAGTTACAGCATTGTATTTCATACATCCATTGTTCGTCGAAGACATCATCAAGATTGATGCTGATAAAAATGAAACGATTGTGATTAAGGCCCTACCTCTCTCATCCTGGTTTCCATATGATGAGCAAAAACACTACCTAGCAAGTTATCTATGGCACGTTTTAGATGGATGTATCGGAGCTTCGTTCGTAACGTACACCGATATTTTCACTTTTAGTCTCATCATATTTCCTTTGGGCCAAATAAATATTTTGAAACACATCTTGGCTCATTTCGGTGAGTATGCCATAAAGATCAAGAATCAAATCGATGTAACTGAAGAGGAAGCAAGTTTTATCACAGTGAGAGAGTGTATTTCGAAACATAATAGTATTATAAGATATATCAACAACTTCAACAAAGAAATG

>AquaOR 17

ATGGTACTAATTTTAGTGAAATCGACAAATATCCAAAATTCAATTGATTTTATATTGAATTATGAGAAGACTCAATTGAAAAATGAAGACATTGGGATAAGACATCTATATTTGAAACATGCCAACTTGAACAACAGGATGACTATTCTGATATTCATGCTCATTTCGTCTGTGGCAACTTTCTGGTATATCACTGGAATAAGGAAAACCTTCAGCGCTGAAGAATCAGAAGAATGTCCATTGATGAAGGGAGTTTTATATCAAATTTGGTACCCAATGATTATTAGAAAATACTATTGGTTAGTATTAATAAACGACTTAGGACTATTATTCAACGCTCTTAATATTACCGTATATTGTAAAGTGATGGTAGTCTCTATGATGGTTTTTATGCTGAGCCAAATAAAAATTTTACAATTCCAATTAACCACAATAGGAAAGAAGTCGCTTGAACAGTCTATGAAAAAAGATTCCACCGAAATTGCTGCCATTATTGGATGTTGCAGAAGACACCAACAGATTTTGAAGTTGATGGACCTTGTAAAATCTGCAGTTAAAGAAATAATCCTCATACAATATTTCGGCAGTACATCAGAAATTGCAGCATATCTTATTCAAACTCTTACTTCGAGAAATGTTTCCGATATACTTAGAAACTGTGCCGCTTTATCAATGCTGATAACAGAAGTTTTCATAGTTTTTTGGTTCGCCAACGAAACGAAAATTCAGAGTGAAGTGATATCTGACATAGTTTATGATGGAATGCCTTGGTATCTATATGAAAAAGATGCTAATATGATTCTTCTACTAATGATGAGACGATCTCATACACCAATGTCCTTCAAAGCAATATTTCTTGGAGATATATCTTTAGCAACGTTCACAAAGCTGATGAAGTTATGTTATTCAGTAGTGATGTGCTTCTCATCGCTAATAGACATA

>AquaOR 18

ATGATGTCAACGTATTTCCTGAAATACACCAAACGCTGCATGTTACTCATGGGTATAATGAAACCAGAGTTTTCCACAATTTACCTAACACATATATACAGGACATGGGGACTCTTCATCGTATGTTGCTACTTTCTCTTCTGTGTATCTTTCACAATAGGAATGCGCTCCGTGCCAAAGGATCTGCTGTTGAAGAACGACGAACTGACTTACTGTTGGACTTTCGCCATGATCATAGTCAAACTTGCCTTTTCACAGACCGATACGATGAGGAATGTAATAGATGAGATGTTCAAGCTGGAAGATGGCATAGACAAGAACGATTCGGGTGCGAAGAAGAAGTATTTGATGCATTCAAGATATAATTTCAAGATCTTCTACGTCCTGGCCAGCATATACTTCGGAACACTGTGCCAACTCTTCTGGCTGATGAAATATAATTGGGCGCAGAAATCCCTTTTCTTCACGGCTTGGTTTCCATTCGACCGTACTTACTATTACACCTTCACGTTCCTTTTTCAAATAGTCAATGGGATCATCATCACTTTCTTCACGCTCACCTTCGATACTCTCTTCACTGCTATCATAATCTTTCCTTCAATGCTGCTGGATATTCTCGGACACAAGTTCGAACACTTCGAGGACTATTGCAAGAGAGAACTGCATCCGAAACTGGTACTTCGAAAACTGATCTTGGAGCATAAGGCTGTTATAAGATACGTTGAGAAGCTCGACCACTCCTTGAAATGGTTCTTCTTCATAGACTTTCTGGTCAAGTCGTATCATTTATCAGTGATGATAATTACATTGGCAACTGCAGCCGATGAGATGGATGTAAATTTTGCATTCACCATGTCCAAATTCGCCTATTGGTCATTGGAAGCATGGTGTTTATACTACCACGGAAATGAATTGATAGTTAAGAGTCTGGCTATTACCAACAGAATTTTCGGAAGTAACTGGTATGAGCACGACCTGGTGGTGAAGAAAATGTTCTTAATCATCATGCTGAGATCCAAAAAGCCTCTGAAACTCCAAGTGGGAGTTTTCCAAACATTGAGCAATGATCTTATGGTGAAGTTTTTCAAAGCTGGGTACTCGGTGATGGCATGGTCAGATAAATTGAGAGGC

>AquaOR 19

ATGGTTTTAGATTTTTTGCAGAGTTCACTGCAACTTGCTTCAGTTGTTATAACACTACTTGTGACGCAAATGACTCTACTAAATTTTATATTTTTTGCTCAATTTGTTCTCTGTATGCTGATAAGGCTGTTAGTTTACTACTGGTACGGCAATGAGATAATTGTTGAGAGTTCAAATATCGCCCTCGCTATATGGGAAAGCAAATGGTATGAAGAGCCAAACAGGGTCAAAAACTTGATGTTGATCATGATGATGAGATGTAATAGAGTTCTTTGTTTGGAAATTGGTCCGTTCAACACGATGTCCCTGAACACTTTAATAGGAATATTGAAAGCAACGTACTCCTACATGATGGTTATTTACAGAACA

>AquaOR 20

ATGAATATCGATACTATCAAAGAAATAGCGGTGCTAAAAAACTCCATGTATTTCCTGAGAATACCGTTTTTGTTTCCACAAAAAGATGAAATCAATGATCCGAACAGAAACGTTTATTTCAAGTTTATCCTGCTGAGCCTGACCACGATATATTTGTCGGTAGGAGCTGCAATACATCTGGTGCTGAGAATTCAAAATGGTACCTATGTCAACCTAGACAGAGATATTGGAACTATCATATCATACCACGGAGCACTTTACTTCACTTTTCGATACTTGGGGAATATCAAAGATATCATTACACTGTATAAACAGTTCTCAGATTTCAAAACTTATGGAACTCCAAAAAACTTCGAAAGAAAAAATAAACTATTGAACAAATATTCGAGGTGGTACTTTGCCTATCACATGTGCGTGGTAACAGGGATGACAACATCCGCACTTCTAACGGTCGACAAGTGTGAAGCAGAAAATATTGAAAAAAACCTGAATGAAGTTTGTGGACTAATTGGCCCAACATGGTTGCCATTTGAATTCGATTATTTTCCTCTCAAGTTTATTGTATACGGCTATCAGGTATACTGCTCCTTTGTTATCTATCAAACTGCGGGGGTCCTTTCTTACACCATGATGGAAACCGTGGAACACCTCATTATCAGATTTGAACACGTGGGAGACACCTTCAAGGAAGCTTTGGCTGAGGAAAACCTTTCCAAACGGAGAGAAAAATTCTACGTGGCAGTCCAATACCACAAAGATGTCATAAAGATGGGGAAGTTATTGAACAGCTGTTTTGGTCCTTGCATGATGGTCCATATAAGTTTGACTGGGCCAGTTCTGGGGGTCGCAGGATATAGATTTGTTACAGAAATTGCTTTGGATTCTACCAGTCTATTCTTCGGGTGGATGTTCTCTACTTTTATTGTTTGTCGGGGTGGACAACGTCTATCAGAAGCGAGTGTTGCTGTTGGTGATGTTATTTATACAGTGGAATGGTACAACTTGGAAAGTGATCTTCAAAAAGATTTAAAAATGGTTATGATGCGATGTCAAAAGCCTGTACTTCTGAGGGCTGGTCCTTTCGGCCCCATGACATTTTCAACGATTGTGGCTATCCTGAAAACTTCATACTCCTATATAACACTACTCAAACAAACGATG

>AquaOR 21

ATGGTAAAACTATTACAGACCTTGAAGATAATAAGAGGCATGGCATGTATCCTTAATGACTCCTTTCGAGGATGTCTTTTCGAGCACCTCTTTTTGGCAGCTGTTGTTCTTGGTAGTGTGGGTTACAGATTGGTTCAGAGCTTCTCACTTGGAGGATTTTGTTTGTATGTGGGTTGGATGTTTTCCTTAGTTATGGTAGGGTACAGCGGTCAACGACTCATAATTGAGAGCACTGCTATTGGAGAAGCCATGTACAACTTCAAATGGTACAACTTGGAATGTGCCCTACAGAAAGACCTGCTGATGGTGATGATGAGGAGTCAAAAACCAATTTTTTTGGAGGCGGGACCATTCGGAAACATGACATATTCGATATTAGTGACAATATTGAAAACGTCCTATTCTTACCTGACTCTCCTCAGAGGGACCAATCATCATAGACAA

>AquaOR 22

ATGAAAATGTTCGAGAAAGAATTTCGAAATGTTTTTAGATTACTGAGTTTTGTCGGCATGCATCCAATGAAGAAATTTGTCAAGCCTTTGGTGATATTTAATAGCGTTCTAACATTTTATGTAACGGTGCTGATCACTCTCAAACTCTTCTTGGGGAGAGAATTGGTTGCTGTTGAATCCTTAGGTGTTTTTTCTCAGATTTGGCTGAAATTCTTCATTCTCACTACGAAAAGAGCGAAAATAAAGCAAGTCATCGAGGATACCCAACTTTTCTGGAAAAATGATCCACCGAACAGCCAAAATCAACAACAACTAAAATACCTCGCTAAATTCGAAAGGATATTCTTAACGTATATATGCTGCAGTACTTGCATGTTCTTGTTCAAACCCCTGTTGGTGAAAGGAACCACTATCTATTACTATTATAGAATACCTCGAATTCCGTTTTACGTTTCCTATGCCATAGAGTTCTATGTGACTTTAGTAACAATGTCTATGGTGATAGCTGTCAATTTATTCATAGCGATAGTTATTGTACTAGGAGCAGGTCAATTCAGTAATTTGAACGCGAATATGAAGCAACTGGACTTGAGTAGAGCCGAAGGAAATGACGAAGGGCTGAGATCTTGTCTTTTGGAAATCAACAAAAATGTTGAATATCACGATTTTCTGATCAGGTACGTACGACGATTAGATGATATATTTTCGATGTTGTTCGTTGTACTCATTGGAATAATAACTGCATTGCTTTGTATGAACATGTACGTCCTGTCCCTGCCTCATACAACAGTGGTTGATATTATTCGTTGTGGAACTATGGTGTGTGCCTTTACTATCGAATTTTTGTTGTTATATGGTGTTCCTGCCCAGAGATTGATGGATGAAGCTGAGGAAGTGGCCAACTCTGCCTTCCATCATTGTCAGTGGTATCTTCCCAATATTATACCAGCAAGAAGATCTCTGACTTTCATAATACATCGAAGTCAGAAATCAGTGTGTCTTTCGGCAATGGGATTTATAGACATCAACCGACAAACTATATTAGCAATGCTGAAAACGGCCTACTCATTTTTTACATTTCTGCAAACCGTTGAATCAACAGGAGAAGCGAAAAAG

>AquaOR 23

ATGATTGATATCTCAGAATCGATGATTTCTATATTCTCGTTCTCACACGCTGCAATGAAATTAATAAACCTCTACCTCAAACGCTCCGTTCTCCTCGACGTTCTGGACAGCATGCGGGACAATTTCTGGAAACTCGAAGATATTGAAGACCACGATGGAACGAATATTTATCTAACGTTTACGAAGGATCTCAAAAGAAAGTTTCATCTATTCCTGACTATGACTACAATTTGCGTCGCTTACTATTTTGTAGCAATACTTCTCACAAACAAGGGTCGTACCGGAGATAACTTACCGTTTGAGTCCTACATTCCTCCCAATGTTTCATATGAAGTTTTATTCAGCCTCCAGTTCTTTGCCGCAATCCTGATGATCATCCCTTTCTTCACAACAGATGTTCTGGTCCTAACCATCATCACGCTGACGTCAATTCAGTTCAAGCTCTTGAACGTGGTTATTGCAAAAATGTTCGAAGGCATGACGGATTCCGATGAAGATGCTCACATTATCAACATGAGACTCAAGAAGTATTGCGATTACCATTCATTTTTGTTGATTTTCAGGAAAAAACTGAACAACATGCTTTCAGCAGGAACGATGGGTTATATGGCGTTAACCATACTGACGCAATGTTTCGTAATGTACGTCGTCTGCTCACAGGGTTCACAGAAAGAAAGCATCAAAGCTATACTTTATGCTACAACGCTGTTTTTCCAACTTTTCATGTGTTACTGCATACCTGCGCAAAATTTGGCAGATGAGGTTGAAAAACTTCCTGACGTCATTTATGGTAGTAACTGGAACCAATATCCCAATCATTCCAAGGATATACTATTATTTTTGGGAAAATCTCAACTCAAAATGTCAATCAGTGCAGGGGGAATCGCAGATATCAATCTACAGACAGGTTTTTCAGCGATGAAAACAGTGGTATCTTACTTCATGTTTTTGAGAACCATAAGTGACAAG

>AquaOR 24

ATGCCATCCAATTTGTTGATGAAATATATCAATCAGTGCTGCACAATTCTGAAGTGGTTGGGAGTACATCCAGAGAAAGTGCAACCTTGGTATTTTTTCTTCACCTTCTTAAATATCGTGATGATATTCCTGGTGATTTTAGTATTATTCTACAAGGATCATGCAATCACTTTCACTGATATAATTGACACTTTCTCCAATTGTGCGCTGATGCTTCACGGAATCGCTAGACTCCTGAATTTCTACTTGAAGCGCGCCAGTTTGTTGGACTTGATCCAGGGAGTCCAACACCGTTTTTGGAAATTGGAAGGGCTGACTACCCAAGAAAGAACTCACTACTACGAGATGATACGCGTGACCAAACTCAAATTCTACTTACTCGCATTTTCCGCCGTAAGCAAATCAATCGTCAACTCTACAATGGCGCTGTTTTCCGACAATGATATGGTCATAATAAATTGTTATAGATTCGAAGCCATATCCCCCTATGTGACATGGCTGTATCAGACTATTTTCACGCATTTTTGTATCCATTTACCAGTGATATCTATGGATTCGCTTATAATGATTTTCATTTCTCTGACGCAAATGCAGTACGAAATGCTTAATAGGGAAATCGAGCGAGTTTTTCGCAGAGCAGTACGAGGAGATAGATATGACTCGAACATTGCAATTGGGAGAATTATCGACCACCACAACTTTCTGATAGAATACACAAATCGCATCAATGATACCTTCTCCAACGTCCTCCTAGCATATCTGTTCTTATATGGTTCATCAATGTGTTTGGAGGTGTACAATTCATCAGAAGTTTCTTCTCTGGAAGCTAGCATGAATGCTCTCACTTATCTCATTGCGGCGACATTTGGGTTCGTGTTTTTATTTTGCATACCAGCACAGAATTTGACAAATGAGGCGAATAAGACAGCCAACGCTGTCTATTTTAGTGATTGGTACCATGAAACAAAACATTCAACTGCGATAATGATGATGATAGCCAATGGTCAGAGGGAAGTATCAATAATGGCTGGTAGAGTTGTAAACATCAATTTGGCAACGGGTTTGGCAACTTTCAAATCTGTGGTTTCCTATTTCATGTTCCTGAGAACTGTTACTACTGTGGAA

>AquaOR 25

ATGTATTTTGGTATCCTCATATTTTCGATATGTGTGGAGATGTTCATGATATCTAACAGAACAACTGTGAAGGAGCTGTTGAAATGTCTTACTTATTCGGTTTCTCTGACGAATGAATTCGTAGTATTTTATTGCATACCAGCCCAGCTTCTGACTTCCGAGGCAGAAGAAATGTATAATTATGCATATGATAGCCAATGGTACAAAAATTCCTCGAGTTCGACAAAATGCACAATCAACATGATTTCTATGAGGGGACAGAAGAAAATTTTTCTTACTGTTGGTAAATTTGGGACCTTAAGCATGGAAAGTTGCTTATCGGCATACAAAGCTGTATTTTCCTACTATATGTTTTTGATGACAATGCAAAGAAAGAACGACAAAGTG

>AquaOR 26

ATGATATGCGCTCTCACCATGGTAACTTTGCTAATTTTGAAGTTCACTAACAAGCATGAAAGTGAAAGAGTAACGGTTATCAAAGTAACCGATGGCGTCACTTGTTTGTTTTTATTTTGCCACGGCATGTTGAAGTCGACTACAATGTTCGTCAAGAAACATAAAGTGCAGGAGCTCTTGAACAAAATGGAAGAACATTTTTGGAAACAAGACCATTTCGAAACCAGCACCATCTATACCGAAGTCCTGAGAATCTACAGAGTTACTACAAACACTTATAATTTTATCTTCACCTTGCATTTTCTCAATGCAATGGGTTTCCTCATAGGGCCTCTACTTACAACGGACACAATCTTACCTTTTGATTGTTATAGACCCGAATGGTCTGGTTACTACGTTCTTCTGCTATTCGAAGATTTGACATCTGTCATATCGATACTGTGTCCTGTGCTCTCCATGGATCTTTTCTTCATGAGTGTGATCAGATTAACCCAAATTCAGTGGAACTTGCTGAATAAGGAAAGTATTTCGATGTTTGATCGTGTGCACGCAAGGGATGGTATTGAAGAAGATACTGAGGCAAAAATTAGAAAATGCATTCATCATCATACCTTCTTACTACATTATGGAGACCTCATCAATGAAACCTTCTCGACTTCATTGTTCCTGTACATGGTGATAATTGTGTTGAGTATGTGTGTAGAGATGTATGTTGCAACTACCATGTCCGAATGGCAGACACTCCGGAAAGCCCTTGTGTACACCGCCACGGGATGCATTGAGTTTATGCTTTGCTATTGTTATCCTTGTCAAAATCTGATGGATGAGGCTGCAAAAATAAGTTCCAGCATATATTTCAGCAAGTGGTACAAATATCCAGAGTATTCGAAAGCCGCACAAATGATTCTGCTTAGAGGACAGAATATGGTTATCATAAGAGCTGGGCGATTCATCACAATGGACTTGAAGACCGGTTTGGCGACGCTGAAGACAATGGTTTCTTATTCGATGTTCCTTCGGACCATGAGTTCAGTTGATAAC

>AquaOR 27

ATGACTTTGAAAAAAGACCTTTTCTTCTTTCTGATATGGCCAGTTGGAGCATGGAAATTGAGAGCATGCCATATAGCTGTGGTGATATTTACAGGAACACTTCAAAGTCTGCAAATTTATTACATCTCTCAAGTTGGACTGCAAGACCCCTGCGTTCTCATAATATTCTTTCTTCACTGTTATGGGATTATTTGCAGTTTGTCATGTATCATATTTGAAAAACAAATGGAAAAATGGGTCGTCAGAATCCTAAATGCGTTCTTGTCGATTGAATTAATGAGCGAATCTGCCAAAGAAGAAATGAAAAAACTCATACGATACGGAAGAATATACAAGGCGGTAGTTGGATTACTGATGTTATTTATCTCCTGCTCAGTGACACCGCGCTACCATTTATGTGTTGTGATATTCAAGGAATATTTTCCTGATGTTGCAACCATTTTGATCGTCGTCTATTTCTTAGATGCGTACTTCATTGCATACGCAATTTCAGCATTGAGTTTCATCATTCTTTATTGTCTATTCCACGTCAAGTTGCAAGAAAAAATAGTGTCTGATGCAATTGATCGAATTTGTGACGGGTTCGAAATCCATGTCGATCTCAATGACGATCTGGAATACCAAAAAATCGTTGAAGCTAGGCTACATTCTTGTTTTCGCCGAATAGTCTGGATCAGAAGGAGCATTTCGATGTTGAGGAAAACCTTGAAAAATGACATTTTTACACACACAATCTGTGGAATGTTAGCTGCAAGTTCGATCATCATTTTGTTCAGTGTGACTGCAGAAAAAAAGCGATACCATAGGGATTCTCACGATTTCTTTGCCATTGGAAGTTGGATTTATCATTTTTGGAATGTTATCGAGCGG

>AquaOR 28

ATGGTATCCTTTTCTGAAGAAGATTACAAGTTACCCGACGTTTTTTATTTCGAAAGAAAAATTCTTTGTTATTTGGGATGCTTCCCCTACCACGGAGAGTGTAGATTATCTTATATGTTGACAGCATTTTTGACTCTTTCAATTTCTGTTTCTATTTATGTGATGATGGTAGTTGGCATTTTCATCGAATACAAGAATGTATCTGTTGTTGCTGAGACGCTTCTTATCGCAGCAACGCAAACTGCGTGCCTCTGCAAAATATTTAATTTAATTATCAATAAGAAAAACATATTAGAATTGGAGCAAATCTTGTTGAAACCGGAATTTTGTAGATTCTCCAAGGAAAATTTGAAAATAATGAAGAATTCCGTGAACGTCGTTCAATACTTTGCGAGAACCTATCGAATTATCTGCTCCATTGCTATCACGTCCGATATGTTCTGGCCACTTCGG

>AquaOR 29

ATGCTATTGTTTATGAAACTCAAACCGGCATTGCCGAAATGGTTGGAACTCATCACCAATGTTTTGGGGTATAGAAAACTATGTAGCAACAAATTAAAGAGACGTATTGAACATGAATGTGGCGTCAGAAGACTACTGATATATACCAGTATAATTGCACCAATCTTCGTCTGTCTCCCATACGGATGGAGGGGAGCAGATGGCCAGCATTTCTTTTTGCTATTTATTATCGAAAAATTTGTGAAAGGGATTGGCATCACTGTGATTTTTGTTGGTTGTTTCCTTTATCCAGTGTTTGTGGCAACCGTTTCTTTCAATGCGAGTATTTATGGATGTACCCATATTTATTTACAGCTGTTGATTCTAGCAGACAATTTGGAAAAACTCAACGTCAGATATACAGAAAACGAGAACTATCAGTTGCATAATGATCAGTATCAGAAAATTATCAGAAGAAAATTGATACTCTGCTGGGAACAGTATTTGAATATAAGAAGGCTCAAAGCCCTAGCTAATGATACATCATATTGGCCAATTTATTCGGGATATATGCATGCTATATTGATTATGGGAACTTCATGCATTTATTTTTTGCTAGGTGGACACCCTTCAGAGAATCCTGGCATCGTTCTTACGCCCATTTTCGTATGGGTGCAAATACTGCTGTTCTTAGAACATGGACAATATATAAAAAATGAGTCAGAAAACATACATATTGCTCTTGGGAAAACTCAATGGTACCAATGGAATACGGAAAATCGGAAATTTTTGTTGATATTCTTGATTGCTACCGAAGAACCAATGCAAATCGAAAGTTTCTGCTGCACTCAAAGTCGTGAGATTTATGTGAAGGTGTTCACTGGGCTATACAGCATCATAACGTGTATCAATACTTTGAGAAATAGAACT

>AquaOR 30

ATGTTGAATTTAAGAATGACATTCTTGATGTTTGCTGTTATCTTCATTGTCAATACATGTGTGCCCTTTCCGAAAATATGTGAGCCGTTCCACAATATGGCCCAAGAATATTTTCACGATTGGACCTTCGTCATCGACATGATAGTATTTTTTATTTTTATCCCATTTACTGGGTATGGACTAGCCTGTGGCGTTTGGAAGCTCACCTTTTTTTACAAACATGGGGATATTCAGTTTTTCATAGTCAATGCATTATTGAATAGTTTAACGACTGAAGCCAATAAAAATCTCAGTGATGACGAATATCAGAGAATAGTATATGAAAAATTGATCCACATAGTGATAGCACACCAAAACGCAAAGAAATTTAACAGAATGATGGTGAACCGTTTCGGATGGATTTTGCACCCTACGTCTATATATGCCAGTCTGGGAGCAGTTTCCTTGATCTATCTTTCTGTTTCAGAATATCATACGATAGGAAATTCTCCCTACTGGGGGACAATCTCTACATTTGTGGTTTTAGGATGCGTTGTATCAGTGTTGATAGATGGTGGACAAAAATTGTTCGATGAGTCCGAATCTGTTTTCTTCAATGCCTATAAATGTCCCTGGTATACATGGAATGAAAACAACAAACGTGTTCTTCTCTTCCTGATGACAAATAGCGTGGAACCAATAAGAGTTTCTTCATATGGTATCGTAAATTTGGACAGATCTACTGCCGTTAAGATAGTGAGAATGGGTTGGTCACTGATGGCAGTCATG

>AquaOR 31

ATGCTGCAGTGGTTACTAGGGCAATCATTAACACTCTCAGGAGGTATTGAGATTTACTGGAATGGAAATCCAAATATGTACAGGAAACAAACCCCATATCTGGTTCTGATTCAAACGGTGACAATGATGATTTTGGTGAGCCAAATAACGTATCGAGAGTATTCGAAGATAATTAAAAAGAGTAGTTGGTCGTGGACCATGGCTGGTGACGAAACGTTCAGAAAGCTCTCGTCGTATAGCAGACATTCTAAAGGGGTATGTTGTACAATTGCTGGCCTGGTCATAATATCGACAGCGCTTCTGATACCAGTCTTCGGAGACGAAACAGAATGGCAATTCACGGCGTATATCTGGAAAAAGTATTTGGACGACCGCATGCATATTTTGTTTACAATTTTCACCGGAACTTTGTCAATCAGTTCAGGGGTATTCATTTCTTCGTTGATCTGTCTGCGATTGTACTGTAACAGACATGTAGAGTGTCAGTTCCACCTTTTGAACGCCCTTATTTGTCGATTACACACGAAAGCATTATATCACGTTAATGATGTCAGATATCAAGAGAGGGTGGATCTCCAACTCAAATACTGCATCAGAGTGCATGTGGAAATAATAAAGTTGTTCAAGCTACTGATGCGTTATGCGAACAGTATACTTTTATCGATGACAATATTTGGCTTGAGTATATTCGTTTCCTCAACATATTTCATTGTGACCGACTTGAGCCCTGAGAGCAACTTTAGGATGTATTTTTTGTTGATGACAGCATGTGTTATACTCTGGGCCTTCTGTGAGAGTTGCCAGAGAATCACAGATGAGTCGGCGATGATTTTTCACAACGCCTGCAAATGCCCATGGGAGCAATTCGACAAACAAAACAGACGTACACTCCTGATGCTACTCCAGAACAGTGCCACGCCTACGAGAGTATCATGCCATGGAGTATATGATGCAAATTATCAGCTGGTAGCCAAGATGTGGAGAGTATGTTATACATCACACACTCTCTTTACCAGCTTGGAT

>AquaOR 32

ATGAATGCGTATCAGCAACTAAAAAGCGGATTGGAAGAAGACCCTTTACAGTTCGTATTTCGATCAAGCTTCCTCACCTGGTTTTCTGTGAAAAACACAAAAAGGATTTTATACTTCAAATGGTTTTCAGCCCAGTTACTAGCAGTCCTAGGTGGCATTGAGATCTATAATGGAAGCCCAGATGGTTACTGGAGATATACACCATATCTAATCATGGCGGAAGCGAATGCGCTGGTATTTCTGATGGGCGCGTCGAATTGTGACCAATGCCAAGATTATTCGAGACTTATAAAAATGCAAGCTTGGTCGTGGAAATTGGCCGATGATGAGACGTACAAGGAGCTGCTGTCGCAAGGCAACCGCCTCAAAGTACTCTGTTGCGCCATGACATGCATAACCATAACTTCACTAGTAGTGTCAGTACCCATCATTGGAGACGAAGCCGATTGGCAAATTACGACGTTGATGTCGAAGAAATACTTGGACGACTGGATGTATACTACGTACATTATTTTGTTTTCGGCAGAGATGCTCATTTTGGGAATATACCTCGCTTCGCTGTTTTCTGTTTGCATATACTGCACCTGCCATATGAAGTTTCAGCTTCAGGTATTGAACTCGATCGTACGCCGGTTACACACCACGTCAGTATTTCACGTAGATGACGTCAGGTATCAGGAGCGAGTGAACGTTCAACTCAAGTGCTGCGTCAAAGTAAATGTTGGGATTCTGAGGCTTATCCGGAACATTACAGAGAGGACGAGCCCTATCGTGTTAGCTATGGCTGGATATGGACTGTATGTATACATTTTCTCGGCATATTTCCTGATGGCTGAGCTTAGTCCAAGGAGCAACTTCAGGATCTATATGTACACCATCGATACTTGTATCGTGCTCTGGGCATTTTGTGAATATTGCCAACAATTCACAAATGAGTCCGAGGAAGTCTTCTTCAACGCCTGCAAGTGCCCATGGGAGCAATTCGACAATCGTAACAGACGTACACTCTGGATGCTGCTTCTGAATAACTGCAATCCTTTGACACTCTCTTGCTATGGAGTCCTGGAAGCAAATCGTAAGCTGGTAGTGACGATGTGGAGGATGGCTTACTCCGTGTGCACGCTTTTCACCAACTTCAAC

>AquaOR 33

ATGGAAAGGGCAAAACGTCCCATGCTGCTTACAACTGGAAAACTGTTGGACCTCTCTTTAACCACATGGACTATGGTATTCGTGATCACACTTCTGGCTCTCTCCTCCGGAGCCGATAAAACATCGAAGCCGAAGCCGAAGAAGAGACAGTCACTTCTATTCGATTCGGGAAACCAATTCTCAATCGATTCAAGCTTTGCCCAACCCAGCATAACTCCGGCTATTTCTTTCGAGTCTTCCGGCGGGCTTGTATCCCATGATTTAGGCACTACTTTCGGCTCCGGATTTGGCTCGGTTGGTTCTGGATTTGGGTCAGTTGGTTCTGGACTCGGCGCTGCTAGTTTCGGGATCGATTCTCTCAGATCCGGCATAGGTTCATTTCCGTCCAGTATTGGCATCAATGGTCTAGCCTCCACTGGTAGTTACGGAATTCCATACCAGACTATTCCAGTTGGTGGCTTCGGAATTTATGCTGGTGCTTACAATGTGCCTTTCCATGCTAATTACGCCTACGGAGTTCCAGCTTTCTTTCAAAATGGAAACTATGGATACGGACAGTTCGGAAATGGGTTAAATTACGGATCAGGTTATATCCAACCAATCCAAAATATTCGCACCATCAACCAGCACGTACCTGTACCAGTACCACAAGCAGTACCAGTGACTGTCACCAAGACTGTACCGGTACCGACTCCATACAAAGTGTCTGTACCAAAGCCATACGCAGTTCCTGTAAAAGTGAAAGTACCAGTAACGGTACCGAAGCCTTACGCAGTCAAAGTAGACCAGAAAGTTCCTGTTCCTGTTGCAGTCAGGGTTCCTGTCGAGATCCCGACGCCATATATAGTCTACAAAACCAAGAAAGTGGAAGTTGAATCAGAGACACCCGTTTTCGTTAAGGATCCCGTGAACGTCAAAGTACCAGTACCTCAACCCTACCCATTCGAAGTACGAACTGGCGTTCCTGTTAAGGTGCCCCATCCAATTACAGTTAAAGTGCCACAATTAGTTGGCGGGCAGTGGATTGGACAAGGATCTTTTGGAACTGTTGAAAACGGTTTCCCAGCTCAAACTCTGCCAGCATTTGGTGGAAGTTTCGACGATCTGAGCGGCTTATATGGATTCGGCAACCCCGGTGGTATCTATGGTACAAATGGCATATATGGTACGAACGGTATCTACGGTACTGATGGTATCTACGGCATAAACGGTATCTAC

>AquaIR 1

ATGCTGAGCATCTTGGAGAGGTACAAGTGGCACCAGTTCTCGGTCGTGACCAGCTTAATCGCGGGCCACGACGACTTCATACAGGCCGTGAGGGAGAGGGTGTCTGCTATGCAGGACCGATTCAAATTCACGATACTGAACGCCGTGCTCGTCGCCAATAAAGGCGATCTGGCTGCTCTTGTCGACTCCGAAGCTCGGGTGATGCTCCTTTACTGCACCAGAGAAGAAGCCATCGACATCCTCACCGCCGCCAGAGATCTGCACCTCACAGGAGAGAACTACGTTTGGGTCGTGACGCAGAGCGTCATCGAGAACCCCCTTCAGGCGCCTTATCAATTTCCCGTTGGAATGCTCGGTGTTCACTTTGATACATCCAGCTCGAGCTTGGTGAACGAGATCACAACCGCCATCAAAGTGTACGCGTACGGCGTGGAAGACTTCACAAACGACCCGACAAATTCCAGACGCTCTCTAAACACACAACTTTCCTGTGAGGGTGCAGGAGCGGCACGCTGGGATACGGGCGACCGCTTCTTCCGCTATCTCAGAAATGTCAGCGTCGAAGCGGACCAGGGCCGGCCCAACTTGGAGTTCACGCCCGACGGAGTTCTACGGGCCGCCGAATTGAAAATCATGAATCTCAGACCGGGGGTCAGCAAGCAGCTCGTTTGGGAAGAGATCGGAGTATGGAAGTCCTGGCAGAAGGAGGGTCTGGATATAAAGGACATCGTGTGGCCGGGAAGTAGCCACACACCACCCCAGGGAGTGCCCGAGAAGTTTCACTTGAAAATTACGTTTTTGGAAGAGCCTCCGTACATCAATTTGGCCCCTCCGGATCCCATCACCGGGAAATGTTCGATGGATAGGGGCGTTCTGTGCAGGGTGGCCAGCGATGCGGATATTACAGAGGTGGACATGTCGCAGGCCCACAAGAACGGCAGTTACTACCAGTGTTGCTCGGGCTTTTGCATCGATTTACTCCAGAAATTCAGCGAGGAGTTGGGCTTCACGTACGAACTCGTCAGAGTGGAGGACGGCAAATGGGGCACCAACGAAAACGGGAAATGGAACGGATTGATCGCGGAACTTGTCAATCGTAAAACTGACATGGTCCTGACGTCTCTGATGATCAACGCCGAGCGAGAGGCGGTGGTGGATTTCAGCGTACCGTATATGGAAACCGGAATAGCGATAGTCGTGGCAAAGCGCACCGGAATTATCTCTCCAACTGCATTCCTGGAACCGTTTGACACGGCCTCCTGGATGCTGGTGGGCATCGTCGCCATTCAAGCCGCTACGTTCACCATATTCCTTTTCGAGTGGCTGTCTCCCAGCGGTTTCAACATGAAGGTGTCTTTCAATCAGAATCCGTCGGCGTCGCACAGATTCTCCTTGTTCCGG

>AquaIR 2

ATGAACAGGGCTTTCAGGGACCTCATGACGTTTCTGAATTGGACCAAGGTAGCCATCATCTATGAAGAAGATTACGGTTTATTCAAGTTACAAGAATTGGTTGAGACACCGGCGGCAACACGGATAGAGATGTACATACGGCAAGCTGGTCCAGCTTCATATCGACAAGTTCTTCACGAGGTCAGGCAGAAAGAAATTTATAAATTGATTGTAGACACGAAATCGAGGAATATCAACCAGTTTTTCCGAGCGATTCTTCAACTCCAGATGAACGACTACAGATACCATTACATGTTTACAACATTCGATCTCGAAACGTTTGATCTAGAAGAT

>AquaIR 3

ATGCAAGGAGTCGCCGACGTCCTCAGCGGGGACATGGACGCCTTCATCTACGACGGCACCGTTCTGGATTACCTGACGTCGCAGGACGAAGACTGCAGGCTGCTCACCGTGGGCTCGTGGTACGCCATGACGGGATACGGCTTGGCCTTCCCTCGGAATTCGAAGTACCTCAAGATGTTCAACAAGAGGCTGCTGGACTTTCGGGAAAACGGCGACTTGGAGAGGCTTCGCCGGTATTGGATGACTGGAGTCTGCAAACCGGGGAAGCAAGAGGTGAAAAGCTCGGATCCCCTCGCTCTGGAGCAGTTCCTGTCGGCCTTCCTGCTGCTGATGTCGGGCATCCTGCTCGCCGCTCTCCTGCTCTTCCTGGAACACCTCTACTTCAAGTACGTGCGTAAACACCTGGCCAAAACCGACAGAGGGGGCTGCTGCGCCCTGATCAGCCTCTCCATGGGAAAGAGCCTAACTTTCCGCGGCGCCGTCTACGAGGCTCAAGACATACTCAGGAATCACCGGTGTCGCGATCAAATCTGCGACACGCATCTGTGGAAAGTCAAGCGCGAACTCGACGTGGCCCAGATGAGGATCAAGCAGCTGGAGAAGGAGATGGAAGTGCACGGCATCAAACCGCCTCCGCCCTGTAAGCGTGTCATCGTTTCCGGAGAGCAAGCCAGAGCTAGATTAAGAACGTTGAACCCAGATAAGGGTAGCGACGTGGATTTATTCGGCCATCGGACGGAAATAGCAGAAATGGAAACTGTGCTG

>AquaIR 4

ATGAGAATGTTTTTGGCGGAAATACTTATAGTTTTGTTGATGCTTTACGTGCTTCGTACAGGTGGCTATGGAAAACCGAAATATAATATTGGTGGAATTTTCTACGATGAAACTCAAGAAATAGCATTCCGAATAGCAACTTATTCCATGAACAACAAGTATCCGGTGTCCCAGATTGAGCTGGTACCCCACACCTACAGAATATCCGCCCATAACACCCTAGAAGCATACAGAGCAACCTGTGAACTGTTCGAGAACCAAACGATAGCTTTATTCGGACCAAATTCTGCTAGTTCGTCGCCGTTCATCCAGTCGCTGTGCGATTCCAAAGAAATACCGCATATCGAAACGCATCGGAGCTTGAATTTGGAAAGGAACGACACCTTGGTGAATCTCTACCCCCATCCGGAGATGCTGTCTATCTGTTTCATGGATTTAATTGAAGCTTTCGACTGGAAGAAAGTCATAATCATTTATGACGACGAAGAGAGTTTGTTGAGTGTCAGTTCTCTGTTGGACCTCAACAACAGGAAAGGAAGGAAAGTTTTACTAAAACAACTGGAGTCGGCCGGTGCCAAAGGAAATAACTTCAGACCAATGCTGAGTGAGGTGAAGGCCTTGGGAGAAACCCAATATGTACTGAGCTGTTCAACTGACATCCTGGAGGATGTCTTGAAGCAGTTACAACAAGTCGGGATGATGACGGAAATATACAGCTATTTGGTCACGGATTTGGACATGCAGACTGTAGATCTGGAAGCTTTTCGATATGCTGGGACCAATATTACAGGGGTCAGGATTGTTGATCCAGAACAGACAGACGTCAAGCAAATAGCTCTGATCGTTCAACCGAATGGTGACGGGCACAAAGTGAGAGTTGAGACGGCTCTGATAATCGATGCTGTGACTTTATTCTACGAGACGATGTACGATCTCACGATTGACAAGAGGATGAAGTTGAAGGCGTTATCACTTGGCTGTGCTTCTAAGAACAGTTGGAGTGATGGATACACTATAATGAACCTGATGAAGTCGAAATCTATGCAAGGAATAACAGGTCTCGTGAAATTTGACCTCGAAGGTTTCCGAAAAGATTTTACGCTGGATGTCCTCGAGTTGTTTCCAGACGGGCTGCTCAAAATCGGGGAATGGAACTCGTCTATTCGGAATATTTACCTCAACCGGCCGGACAGAGTGAAGTACGGAAACAATAATCCCGGAGACAACCTCTTCAACAGAACGTTCACTGTTCTCATAAGTATAACTCCTCCCTATGGAATGCTTAAGGAAACGACGATGCAGTTGAATGGAAACGACAGATACGAAGGTTATGGGGTGGATGTCATCTGCGAGCTCTCCAAAATTCTTGGATTCAACTACACTTTCGTCCTACAAGAGGACGGACTTTATGGAAACTTCAACAAAACTTCGGGACAGTGGAATGGGATGCTCAGAAAAATTATTGATGAGAGAGCTGATTTGGCGATTACGGATTTAACTGTCACATCTGAGAGAGAGAACGCAGTGGATTTCACCATGCCTTTCATGAATTTAGGCATTAGTATTCTCTACAGAAAACCAGAGCCAGTTCCGCCCAGCTTGTTCATGTTCGTTTCGCCGTTTTCGTTCAACGTGTGGATCTTGCTAGCCGTTTCGTACTTCGTCGTCTCCATCAGCTTCTTCGTAATGGGGAGGCTATCGCCCACCGAATGGCAGAATCCTTTTCCGTGCGTGGAGGAACCCGAGTTCCTCGTCAATCAGTTTACCGTAAGGAACTCCCTGTGGTTCACGGTGGGAGCTTTGATGCAGCAAGGATCGGAGCTAGCACCAATCGCAATTTCTACAAGAACAGCTTCTGGGGTATGGTGGTTTTTCGTGCTGGTAATGGTATCCTCTTACACTGCTAATTTGGCTGCTTTTCTGACAGTCACAACTCTGGTCACCCCATTCAAAGACATCGAAGAGCTAGCGAATCAACAAGAAATCCAATTCGGAGCGAAAAAGGGTGGTGCTACAGCGCAATATTTCAGGGACTCCAATCTATCGAAATACCAGAAAGTCTGGAGCTACATGGTCCAGCACCCGGAGCTGATGATGGACGACAACGATGCGGGCGTGACCAAGGTGGAAAGAGAGAACTACGCCTTCCTCATGGAGTCCACCACCATCGAGTATAATACGGAAAGGCACTGCAGCTTGGCTAGAGTTGGCGGGTTGTTGGATGACAAGGGATACGGGATAGCAATGAAGAGAAATTCACCATATAGGAACGATCTGAGCACCGCTGTTCTACAATTGCAAGAGAAGGGAATACTCACCAGTCTGAAAATAAAGTGGTGGAAAGAGAAGAGAGGCGGCGGTAAATGTTCGACAAAATCAGAGGACAGCGAAGCCACCCCCCTGGATCTGCAGAACGTCGGAGGAGTATTTTTGGTATTGTTCGTAGGGGCAATACTGGGAACCATAGGTTCTTTCGCAGAACTGGCGCTGAGGCTTTACAGGAGATCGCATAGAGACAAAGTTTCGTTCAAGGAAGAACTGATGAAGGAGATGCGGTTCTTCGTTCAGTTCAAGCTGAACGTTAAAGAAATTGACGGTACTCCACACGATAGTACGAGCAATAAGAATCTCATCAGTAGTACAAAATCTTTGGATACTGTGAAAATTAGT

>AquaIR 5

ATGTTTTGGGAAATATGTTTTCTGAGTTTGGTTTTTCACCATGGATTGGGTACTATCACGAAACTCAATATTGCTGGAATTTTTGAAAAAGTTCAATTACACCAGTCAGCATTTTTGTACTCCAACCAGCTCCAGGATATCCAAAGACAAGTTTCCAACACAACCTTGCTACCTCTCGTCGACGTTCACGTTTTCCCAGACGACGCATTTTCTGCTCGGAAGGCAACGTGCTCTTTTCTGGAAAAACACATAGTAGGAATTTTCGGACCCCAGTCCAGCTCAAACTTAGATATAGTGCAGTCCATCACTGACAGAAAAGAAATACCGCACATCCTAACGAGATGGGTTCACCCTTCCCATATGGCACCAGAAACCATCAATTTCTATCCTAGTCCAACAAGACTAGCCGAAGCCTTCTTAGACATACTCAGGAAACTGGAATGGAAGACCTTCACCATTCTGTACACAGATTTCGAGAATCTCATACAAATCACCGATTTCATCAACGAGGCTAAAGATCAAGGGTTTATCGTTTATATGGAGGATGTCAATGCGTATCAGGATGGAAACTATAGGCCAATCCTCCAAGAAGTTATGAAATCTGGCCAGAAAAATTTTGTGTTGGATTGTCCCATTCAATACCTGACGGAACTATTGGCTCAAATTCAGCAAGTGGGAATGTTAACAACGGATTACAATTTCTTCCTAACGAACCTGGATGCCCATACAAAAGCTCACACTGGAGATCTAACTCCCTTCATGCATAGTGACGCCACCATAACAGGGGTCCATATCATGAAACCGGAAGACGATCTGTCAACACGGGTCAGTAAAGAACTGTGTCACCTGTACAAAGTGACTTTCAACGAGGACTGCGGATCAAATGCGCTCCACGACTACCCCGAGTTGGATTCGGAAACAGCTTCAATCATCGATGCTGTCCACGTATTTTCAGATGCCCTGAATGCAGCAGGAGTCAGCCAGAGTCAAGCCCTGGACTGTAACGGGCCAGATAGCTGGCAGAACGGACTCAGCATAATCAACGCTTTGAAATCGAACACTTTCGAAGGCCTTACTGGTACCATAGAATTCGGTAACGATGGTTTCCGCAGGACTTTTCAGTTGACCATATTCCAACTACAAGATGGAAGATTGGTGGACAGAGGAAGTTGGAACACCACATCAGGGATTGACATGAACATTGATATGAGGGCAATTGATGTCGAAGACAGCGAAGAAAGCATGATGAACAAGAAATTTGTTGTTCTAATAACTTTGACAAAGCCATATGCTATGTTGAAAGAAACTCCCGAACGTTTGGTGGGCAATGATAAGTATGAAGGTTTTGCAATAGACCTGATAGAAGAGATCGCCAAGATAGAAGGATTCGAGTACAGTTTCAAAGTCAGAGAGGACAATAAGCATGGTGTCTTTGATCCCGTTTCAGGAAAATGGACAGGAATGATTGGAGATATAATAGAAATGGGAGCTGATATGGCTATATCAGATCTGACTATAACTCAGGACAGACTGGATCCTGTGGAATTCACCCAGCCTTTTATGAGTACAGGTATAAGCATTCTCTTCCACAAACCAACTGAAATACCACCCAAGTTCCTGTACTTCACAGAGCCGTTTTCAGCAGCTGTATGGCAAGCGTTGGGGTTATCCTATCTTGCACTAGTTCTAACCTTATTCATCACCGGAAGACTAGCTCCGACTGAATGGTATCAGCGACCTAACAAAAAGTATCTGGTTAATCAGTTGTCTCTATCTAACTGTTTGTGGTTTGGAGCAGGCAGTTATTTCAGGCAGAACGCTGACGTGAGGATGAGTTCACTGTCTTCCAGAATCATTTCTGCCTGTTGGTGGATGATATGCTTTTTCACGCTGGCAATGTACATTGCTTTTTCAATATCTAGGAGCTCTATAGCAGAAAAAGAAGTGCTTTTCAACGATGCCAACGAGTTGGTTGAGAAATCAGAGCAATATGGGATCAAATATGGTGCTCTCCATGGAGGAGCAACACAAGCGTTCTTTGAATCTTCTGCTTCCGACGTTTACAAAGAAATTTCCACCTATATGGCCGAACACCCGGAAGATATGCCCTCATTAACAGAAGAAGGCGTAGAAAAAGCAGAGAAGGAAAATTATGCTTTCTTCATGGAATCAGCCACGATAGAGTATGTCATCAAGAGACATTGCAGATTGACTACTTATGGCAGCTTGTTGGATAATAAGGGATTTGGAATAGCTGTGAGAAAAGGTTCACCCATACTGACCCCACTGAATAAGGCTATCCTGACACTACACACGTCAGGTGATATTTTGAGAATTAAAAGGAAGTGGTGGGAAGAGAGAAATGTCAAAGAAATTTGTGAGGATGATTCCGAAACAGATGCTGCCCCCAAAGAACTTTCCCATGTTATTGGACTGCTTTGGATAACACTGTTTGGGACGTTATTGGCGTTGGTCTGCTCGGTTGTGGAATTTTCTCTCTACGTATACAGTTTGAGTACAAAACTTAAACAAACGTTTGGTCGAACATTTTTTCAGGAACTAGGAAGCTCTTTCAGGCGCAAGAGGATACATCGAGAAATTCAACCAATATTACTAGTCAAGACCGAAAATGACCAAAATGCATCCCCCGAATCAGTGGAAATGTCT

>AquaIR 6

ATGATGGAATCCTATCAAGGAGTGAGTGCAGTTTTTGGAGTGGATTCTCCCAACACGTTTCCCATACTCGAATCAATCTGCACAAATTTCGAAATTCCGTTCATAACAACATCATGGAAACCTACAATTATAAGAAACCCTGATCAAGCGAGGGCCTTGCTCAATTTTCATCCCGAAGCCGATCTATACGCAAAAGGCCTAGCTGAAATGGTAAAGAGTCTGGAATGGGCCAGTTTCGTTATTGTTTACGAAACTGAAGAAGGACTTATTAGGATGCAAGAAATACTGAAACTGCAGGAACTGAAGAAAGGCAATAAGAAAAATACAATTTTCGTGAAACAATTGGGACCAGGACCGGATTACAGGCCACTCTTCAAGGAGATTAGAAACACATCTGAAGATAATATAATTTTGGATTGCAAGACGGAAAATATATTACCTATATTATTACAAGCCAAAAGCCTCAATATGCTCAGTTTACATAACCGTTACTTCATCACATCTTTGGATGCACATACCATTGATTTCACGGTTCTGAACACTACAGCTAATATCACATCAGTCCGGTTACATGATCCCCATAGTGATGATTTCAAAAACATCATTCATCGGTGGGAGCTGACCGAGTTCGAAAATCGAAATATTCACATGCAACTAGACCCAAGATCTATAAAGACGGAAACAGTATTATTTCATGACGCTATTCTTCTGCTTAGCGATTCCATCAGTGATTTGTCAGTTAAGCATGGATTGAGGACAAATCCTATTTCCTGTAGAGAAAATGAAACCTCGCTCAATGGTTTTTCGCTGAGGAACTACATGAGAATAAGGACACCATCATTCACTCTCTCAGGACCAATCAGCTTCAATGAAAATGGGGATAGAATTAAATTCAATCTTCATTTGGTTGATACAGTGGACGAAACAGTTTTAGCCACCTGGTTTGCTAGCAATAAGACTGTGAAAGTGACCAGAAATTATGATGAAACAGTTGACGCAGCCATTTTGAACTTGCAGAAGATCAAAGTGATAGTATCGTCAAGACTTAGTCCTCCATACCTTCAGAAGAGAGAACCAAGTTATGAGGGTGAAGAACTAGTAGGAAATAGAAAATACATGGGATACTCAATGGATCTTATAGACGGAATCGCAAAAATTATTGGATTCCAATATGAGTTCCAAATAACTGAAAAACATGGTAGCTACGATTATGATCTTAAGAAATGGACTGGATTGATAGGAGATCTTCTGGAAAAAAAAGGCTCATCTTGCTATTTG

>AquaIR 7

ATGTGTAATAATGTTTTCCGGGTAAATCTGATTAAAACTTCTCAGTTTTTAGTAATGGTGGAAATGTTTTTGGGTATGATAACATTATGGGCCTTCATCCAGGGTTTAATATGTGAAGAAGTTAATATTGCAGGTTTATTCGAGAATGACGCGAATCTTGAGAAGGCTTTTCTTCACGCAATCGACCTACTGAACGAAAACCAAAACGAAGATGAGTTCACCTTCACACCACTGACCCAGAACGAAATTCTCGAAAATGAACCGTACTCAGCTCTCCACCACACGTGCCTACTTCTGAACCTAGGGGTGGTTGCCGTTTTCGGACCGAGATCCTTCGACAATATCGACATCGTTCAGTCCGTTTGCGACTCCAAGGAAATACCGCACGTAATCACCAGGTGGAATTACTGGTCTTCGAGACAAAGCTCTGAGATCAACTTCTACCCTCACCCTCCCTTGCTCACCAAAGCATACTTGGATATAATATTATCGTGGGAATGGAAGACGTTCACTGTTCTTTATGAGGATGATGAGAGTCTTGTGAGGGTAACTGGTTTGATCGAGACAGCGAAATCACGAGGAGTAGTGGTACAAGTTTTCCAGTTAGAGCCAGGAAATTACAGGGCCATTATCAGAGATTTAAAGAACTCCGGTGAGAAATTCATAGTTCTGGATTGCCAAATAGAACACCTCATCGAAGTATTGACGCATTTACAGCAGGCTGGTGTCATGAATGAACATTACAACTATTTCATTACCAATTTAGATGCCCATACTGAAGATCTAACGCCATTCATGTATAGCAATGCGAACATAACAGGGATCCGAATGATAAATCCAGACAAGGAGTCCGTACAGCGTTCAACCAGAAAATTATTTTCCGAAGACGAATTCACAGGAGCTTGGAAGCTGAAGATAGAGCCTGCGCTCATTATTGATGCGTTACAAATGTTCGCTGACATTTTCAACCACAGACAGAATTTGAGTCCAGTTTCGATCGTTAAAAATAGCAACAGCTTGCCGTGTTATGATACTGGTAGTTGGGAGCACGGCTATAGTGTAGTGAATATGCTGAAAACTAGTTCCTATGACGGTCTCTCTGGCCTAATCAGATTCAACAATGAAGGTTTCCGAAGTGAGTTCTTGTTATACATTTATGAACTGAGAGAGGGAGGACTGACTGATGTTGGCAACTGGAATTCCACCAGTGGACTGAATCTGACCAGATCACATTCATCTCAAGAAATATCTGACGAAGAAAGTTTGAGGAATAAGTCTTTCAATGTTCTCATAACTTTGACTGAACCCTACGGTATGCTGAAACAGACAACGAACCTTCTCACAGGAAACGATAGGTTCGAAGGTTATACAATAGACCTAATCCAGAAACTATCTGAACTTGAAGGTTTCAATTACACTTTCATCGTCAGAGAGGACAAAAAAAAATGGAGCGTT

>AquaIR 8a

ATGCTTCTGCCGCAAAAAAAGTTGAGGGAGTTTCGCCCCACCCCGAATAATTATGCGATAATTGCCTCAACCAAGAATATGCAGAAACTAATCAGAGTAGCGTTCAGAGAAAACCTAGTGACTCTACCAGAAAGATGGAACCTGGTTTTTCTGGATTTCCAACACAAATCCTTCGACCGTTCTTTGATAATGAGCAATCCAGTGAACTTACTAACCCTGGATACTGAGCTTTGCTGTCAACTACTGAATCAAAACACTTACTGTGAATGTCCAGGAAGTTTTAGCGCCCCCAAAGAACTGTTGCGGATAGCGATAAATGTATTGGCGAACACCTTCGAAGAACTACTTCAGAAAGGCGTTCAACTGGGTGATATCGAATGTGATACCAACACTACTAATTATAATGAAACTAGTTTAAAACATTTCGAAGAAATCATGAATGATTTTGTCAGCAAACACAATACAATCGGGATGGATAATTCAATTCTACATTTGAAAGCTACAGGTTCCATTGGAATTGGTAACGAAGTTTTTGCCAAGTACAAAAATGAAACCATAAGCGTCATAGGAAATAAAACTGTGAAACCAATAAGAGCGTTCTACAGAGTGGGTATAACACACGCTTTACCTTGGTCCTACAAAATAAAAGATTCGGATACAGGAAAATGGATTTGGACGGGATATTGTGCCGATTTTACTGCGAAACTTGCCGAAAAAATGGACTTCGACTACGAATTTGTTGAACCAACCAAAGGAACTTTCGGCAAAAGAAAGGACGGTGTTTGGGATGGTGTTATAGGTGATTTGGCATCAGGACAAACTGATTTGGCTATCACGGCAATCATCATGACTGCTGATAAGGAAGAAGTCGTTGATTTCGTCGCCCCCTATTTTGAACAAACCGGCATCACAATTGTGATGCGGAAACCGGTGAGGAAGACTTCTCTCTTCAAATTTATGACCGTGCTCAAATTAGAAGTGTGGCTCAGCATCGTGGCCGCCCTGATTGTTACCGGGTTTATGGTATGGTTTCTCGATAAATATTCGCCGTATAGTGCCAGAAACAACAAAAAGGCGTACCCTTACCCATGCAGGAAATTCACGTTGAAAGAGAGCTTCTGGTTTGCTTTAACGTCTTTCACTCCACAGGGTGGGGGTGAAGCACCCAAGGCCCTTTCTGGCCGAACTCTAGTAGCAGCATACTGGCTTTTTGTCGTTCTTATGTTGGCAACATTCACTGCAAATTTGGCTGCTTTTTTAACGGTGGAACGCATGCAGGCACCTGTTCAGTCTCTTGAGCAGTTGGCCAGGCAATCAAGAATCAACTATACGGTGGTAAAAAATTCCCAAACTCATAAGTACTTCATCAACATGAAGTTTGCTGAGGACACACTTTACAGGATGTGGAAAGAACTCACTCTGAACGCTTCCACGGACGACAGCAGATATCGCGTTTGGGATTACCCAATCAGGGAGCAGTACGGACATATTTTGCTGGCCATAAACGATTCCAATCCGGTAGCAAGCGCCGAAGAGGGCTTCCGAAACGTCGACGAGCATTTGGACGCCGATTACGCCTTCATCCACGACTCGAGCGAGATAAAACACGAAATCAGCAAGAACTGCAACCTGACGGAGGTCGGCGAAGTGTTCGCGGAGAGGCCCTACGCAGTGGCGGTGCAGCAGGGGAGCCATCTGCAGGACGCGATAAGTAAAATGATTTTACTTCTTCAGAAGGATAGGTTTTTTGAAGAACTGCATGCAAAATATTGGAATAATTCCGCCAAAGGTGACTGTCCCAATACAGATGACAATGAGGGAATTACACTAGAGAGCTTAGGTGGTGTGTTCATAGCTACCCTATTTGGCCTGGCTTTGGCAATGATGACCCTTGCAGGAGAGGTATTATACTATAGAAGAAAACGACGGAATACCCAACTCAAAGATAGAGAATCCAAGTCAAAAGTGTTTCCTGGAAAAGCACAGGAACTTTTTCATCAAAAACAGTTTCCATTAGAGAGCAATTCAATCACTATCGGCAGCACATTCAAACCAGTGAACTTGAATGAGAAAATCAGAAGAGAACGAGAGGAATTGAAGATATCCCATATAACTTTGTACCCACGTGCAAGGACTCGCGTATCTCAAGCTGAAAAGAAT

>AquaIR 9

ATGAATGACTACAAGTATCATTACCTCTTCACGTCATTTGATATGGAAACGTTCGACTTGGAAGATTTCAAGTATAATTTCGTGAATATGACCGCCTTTCGGATAGTAGACACCGAAGATTTGTCAGTGAGGGACATGCTCAGGAATATGATGAAATTTCAAGCGAGTGAAGGGGTTCAGCTGATAAATTCCAGCAATATTCAGGCGGAAGCCGCATTGATGTACGACTCAGTGTTCGTTTTTGCTGTTGGACTCCAGATGCTCGATCAATCGCACAACCTGGAACTCTTGAATATATCTTGCGACAAGGCGCAGCCATGGGACGGTGGACTTAGTCTCATCAATTATATAAATGCGGTAGAGCTGAAAGGTATAAGCGGACCAATAGGGTTGAAAGAAGGCCGGCGTATTCAATTCAAACTGGATCTACTGAAGTTGAAACAGCATGCTTTGGTGAAGGTCGGCGAATGGCATCCTGGTTCTGGGGTTAACATCACGGACCGTGAAGCTTTCTTCGATCCTGGAATTATGAACGTGACTTTAGTGGTAACGACTATCCTGGAGACACCTTACGTTATGATGCACAGTGGAAAGAATTACACCGGAAATAGTCGATTCTATGGTTTCTGTATCGACATTCTGGAACGTGTCTCGCAAGAAGTCGGATTTGATTACCTACTCGATTTAGTACCTGATAGGAAATATGGAGCACAGGACCATCAAACGGGTTCGTGGAATGGAATGGTATTACAGCTCATTCAACACAAAGCTGATTTGGCGGTGGGCTCTATGACAATTAACTATGCAAGAGAAAGTGTAATAGACTTCACGAAACCCTTCATGAATCTTGGCATCAGCATTTTATTCAAGGCAGCCTCAACAAGAATCGTCGGAGGAGTTTGGTGGTTCTTCACGTTGATCATGATATCATCCTATACCGCCAATCTGGCAGCGTTCCTTACTGTTGAACGAATGATCACACCCATCGAAAACGCAGAGGACCTC

>AquaIR 10

ATGTACGGGGTATACGATCCCGACACGAAAGAATGGAATGGCATAGTCAGAGAACTCATGGAGAAGAGAGCGGATCTTGCTGTTGCATCCATGACTATCAATTATGCCAGAGAAAGCGTAATCGATTTCACGAAACCATTCATGAATCTTGGGATCGGAATTCTGTTCAAGGTACCAACAAGTCAGCCCACCCGCCTGTTCAGTTTCATGAATCCCCTGGCAGTGGAAATTTGGCTGTACGTCCTAGCAGCCTATATCTTGGTCTCATTTACCTTATTCGTAATGGCCAGATTTTCCCCATACGAATGGAATAATCCGCACCCCTGTCATCAGGAATCCGACATAGTCGAGAACCAGTTCTCGGTATCGAAC

>AquaIR 11

ATGATCCAATCAGTGTCTGCCAGTCTGCAAATTCCACAGTTTCAAACATTCTGGAATCCAAAATTGAGGAGGCCTTTGGCAGAACCGAGTGCGTCCAATCAAATCTTCAACCTGCATCCCAGTCCACGAACCCTGTCTCAAGCGTTAGCGACTTTGGTGCGCGAGAACGACTGGAAGAGTTACACTGTCATTTATGAGAACGACGAAGGACTCCTGCGGATGCAAGAGGCCCTGAAGCAGCGAAGCCCCGCCGATCCCGCTGTAGCCTTCAGAGCGTTGGGGCCCAGAGAGAATCACAGGTCGGTGCTGAAAGAAGTTAAGAGCTCCGGCGTATTGCACGTAATACTAGACTGCGATGCGGGCCGTATAATGGACATTTTACGGCAGGCTAAGAAACTTAAATTATTATCGGAATTCCACAGCTATATACTGACAAACCTGGATTCCCATACCCTAGACTGGTCCGAATTCAAGCATATCAGAACGAATATAACCGCACTACGATTGGTGGATCCTGATAGCTCCAGTGCGCAGCATGCAGCTCTGGTCTGGAATCAAATTATGAAAATCGATATTTTGCGAAACATACAGAACCCTGCCATTCAGAAGGAACTTGAAAACTATCGGAAGCTCAACCCAGTAGCGGAGATTCTCCCACAGAATATACCGATCAGAACAGCTCTGCTTTATGATGCCCTGAATCTTTTCGTATCCACATTCTCTAAACTAGACGCAGAAGAGGAATTGATTCTGGATCCGCTGAGCTGTGGAACAAATGAGACTTCATCGCATGGACGGCGCTTTTCGGAAGCTCTGACAGAGAAAAAGAGGGACATAAAACTCATCGTGGAACCACTGACAGGACAAATAACTAAATTTGATGAGTCAGGTTATAGGAGAAAGTACAAACTGCAAATCATAGAGTTCGATTCAACGAAATTTCGAGTGACAGGTACGTGGGACTCGGATTTTCCGCAAGTAATAAAATTAAAACTGTCGGAAGAAGATCGAGATACAGAGTTGAAGAAAAAAATCCAACAAAGGACATTCAGAGTCATCTCGAGGTTGGGTGACCCCTATCTCATGCGCAGAACTGATCCGAACGGAAGACCTTTGTTCGGAAACGATAGATACGAAGGTTACACCATGGACTTGATGGGTGAGATTTGTAAACCTCAGAACCTCAACTGTAGCTTCTCCTTCGAAATAGTCGCAGATGGCAAATATGGAAATTACGATCCGATCACGAAGCAGTGGAATGGTCTCATTCGAGAATTACTCGATTATAAAGCTGATCTTGGCGTTTGCGACCTGACCATAACGTATGAACGAAGAAAAGCTGTAGATTTCACAATGCCTTTTATGACTCTGGGAATCAGTATTCTGTATGCGAAAGCTGTTAAGGAACCGCCGGAGTTGTTGTCGTTCAGCCATCCCCTATCATTCGAAGTCTGGGTCTACATTGCTACTTCGTATTTGATTATTTCCATGATGATGTTCCTGGTGGCAAGGTTGAACCCCAACGACTGGGAAAATCCTCATCCATGTAATCCTCGCCCCACGGAACTGGAGAATATTTGGAATGTGAAGAATTGTTTTTGGCTGACAATGGGCTCATTTATGGCACAGGGCTGTGATATCCTTCCCAAGGGAATTTCCACTAGGATGGTAGCGGCCATGTGGTGGTTCTTCACCTTGATAATCACTGCCTGCTACACGGCAAACATGACCGCTTTCTTGACCATGTCCCGGATGGGCCCCACAATAGAAAGTGCGGACGATTTAGCAGCACAGACGAAAATTAAATATGGATGTCTAGGAGGGGGTTCGACATCATCTTTCTTCAAAGACACGAATTTTTCCACTTACCATCGGATGTGGGTACAAATGGAATCTGCAGAGCCGAGTGTATTCGAATCGAACAATAAAGACGGAGTGAAGCGTGTTTTAACCAGTAAAAGGAAATACGCTTTCCTCATGGAAAGCTCTTCGATCGAATACGAAATGGAGAGGAATTGCGAACTGATGCAAGTTGGGAACAACCTGGATTCGAAGGGATACGGAATTGCAATGCCTACAAATGCCCCTTATCGAAAATCTATCAATGAAGCTATTCTGAAAATGCAGGAAATGGGCCTATTACATAAACTGAAGGATAAATGGTGGAAAGAAATGAATGGTGGGGGACAATGTACGAAAGACAAAATCGGCCACGATGAAACAGCAAACGAAATGGGGCTTGACAACGTTGGGGGTGTCTTCGTGGTGCTTGCTGCAGGTGTCGCATTTGCCTTCGTGATTGCCGTCTGCGAATTCCTATGGAACGTCCGGAAAGTTGCTGTCGTTGAAAAGCTGACACCGAAGGAAGCCTTGATCAAAGAACTCCGATTTGCAATGGACATAAGTTCAAGGAAGAAGGCCGTATTTGCTCCTTCTCGAGCAATTTCGTTGGAAAACATAGACAGA

>AquaIR 12

ATGTTTGCTTTCTTAGATCCCTTTGCTACAGCCGTTTGGATTTATTCAGCAACATTGTATTTGGTAGTTTCCGTGGTGCTCTTTTTCATAGCTAGAATGACCCCTGGTGACTGGGAAAACCCACATCCATGTGATGAACAACCTCCCGAACTCGAAAATATCTGGGATATCAAAAATTGTCATTGGGCAACTATGGGGGCGATAATGAACCAAGGTTGTGATATTTTGCCAAAGGGATGGTCATCTAGAATGGCACTCGCAATGTGGTGGTTTTTTGCATTGATCATTACGAATTCTTACATTGCCAATCTTACCGCCTTTTTAACCAAAGATAAAATGGATCCGCCCATCAATAATGCTGAGGATTTGGCGAAGCAAAATAAAATCAAATATGGAATGTTAGAAGGTGGATCAACAGAAAACTTCTTCAAAGATTCCAATGATTCTATCTTCGAGAGAATGTACATAAATATGAAATCCCAAAGACCCAGTGTTCTGGAGAAAGAAAACAAAGATGGAGTCGCCAGAGTTTTGAGTACCAAGAATGGATTGTATGCGTTTTTGATGGAATCCACTCAGATCGAGTATGAAATAGAAAAAAACTGTAGCCTGAGGCAAGTTGGTGATTGGCTGGATAGTAAGAGCTACGGAATTGCGATGCCTATGAATGCACCATATAGGGGAGCTATCAACAAAGCTGTTTTGAGATTACAAGAACTTGGAAATCTGACCGCTTTGAAAACAAAGTGGTGGAAGAAAGCCAAAAAAAGGGGAATCCTG

>AquaIR 13

ATGGGAATCTTTGAAGCAGGCATTATAACAAAAATGACCGAAAACGAATACGAAAAACTTGGGAAGCAGAAAGAATTGTCCTCATCAATAGCGGAAAATGTCCAGAAAGAAAATACGAAGGAGAGCCGTAGACAAACAAAAGTTAACGAAGAAACTGATGAACTCAAACCCATCAGTTTGAAAATGCTACAAGGATCCTTCTATATACTGTGTTTTGGTAATATATTTTCAGGAATGATCCTGGTAGCAGAATTGATGTTCCACAAAAATCAGATAACACATAAGTCTAAAAGAAAAACATCCATCAAAGTGAAGAAATTGGGGGAGCTCATCAGGGTCCATATCAATCAACTACGTTCGTTTCTGAGAAGACTTCATCAGAATATAATGCACGATGCTTTCTTATCCACTCTAGAATATATGGAA

>AquaIR 14

ATGACATTTTTCAGGGATTCGATGATTGAAACTTACAAAAAAATGTGGCGATTCATGGAAAACCGAAAACCATCAGTATTCATGCCAACATACGAAGAAGGTATCCAGAGAGTTATCGAAGGAAATTATGCTTTCCTCATGGAATCCACAATGCTCGATTTCATTGTTCAACGAAACTGTAACCTGACTCAAATAGGAGGGCTGCTGGATTCTAAGGGATATGGGATTGCCACACCCAAAGGGAGTCCTTGGAAGGATAAGATTTCTCTCGTCATTCTGGAATTACAAGAAAAGGGTGAAATACAAATGCTGTATAATAAATGGTGGAAAAAATCAGGGGAAACCTGCGAAAAAAATGAAAAGAAGAAGGGTTCCAAAGCTAATTCATTAGGAGTGGATAGTATCGGGGGAGTTTTCGTGGTACTTTTGTGTGGACTGGCTTTTGCGGTTTTGATTGCGATATTGGAGTTTTGTTACAATTCTAAAAAATATAAGAAGTTTCAGGGCCAATCCAGAGCTCCGAATCAGTCGCTCTGCTCAGAGATGGGGGGTGAATTCTGCTTCGCTTTGAAATGTTGCGGGTCAAGACAACGGCCGGCAATTCGCAGAAGGTGTTCGAAATGTCTTCCTGACATCACGTACGTTCCAGTCAAAAATTCCAGACATCAAACCCATCCGACGGTTGAATCAGTGCCGAACAGCCAGCAGATATTCGAAGAGACAAGGATACGAGAAGTGCAAGTGCGAAGT

>AquaIR 15

ATGACTGGAATAAACTTCAAGTCTGCTGTGGTGATGCCAACTTTGGACATGCCACTGAAGGAATATTTGGCGAGCGACAATGATAGACAGTTTAATTCCATGCATAGATTCCAAAGTGTCACCGTTAACCATTGCAAAGATTTATACAATTTCAGTTTGGATATTCAAAGAACGAACTCATGGGGCTATATTCAGGCAAATGGCCGGTTTGACGGATTGGTGAGTCTGCTAGAAAAAAGACAAGTGGATTTTGGTAGTTCCCCACTTCTATACAAGTTGGATAGAATGCCTTATGTGGATTACAGTTACGGCAACTGGATATTGAGGTCAACTTTCATCTATCGCAGACCAAAAGTTACTGCCAAATCATATGAGATATTTCTCAGACCCCTTGAAAAAGATGTTTGGATAACTATTTCGATAATC

>AquaIR 16

ATGAACAATCCCTTCGAATCAGTATCGGCAACCTGTCAGCTCCTACAGGAAGGAGTCGTTGGAATTTTGGGTCCATTTTCCGAAGACAATTCGAACGTGGTTCAATCTGTTTGTGACTTGAAGGAGATACCTCATATTGAGGTGCGATGGGACGATTATCCTCTCAATGGAACCGTGGTCAATATACATCCTTATCCAGACACCCTTACCAGAACGTACTACGATATTATAGTCGGATGGGGATGGGAGGATTTCGTTATCCTATACGAGAACAATGAGAGCTTGCAGAGGGTAGGAGAACTCTTGAAGCTTTTCGAACCTGCCAAGCAGAGAATTGTGGTCAGGCAGCTAGACGCAAAGGAGGAGTCGGAAGAAGGATTCAGAACAGTTCTAAAGGAAGTACGGAAATCTGGAGCAACACATTTCGTGTTAGATTGCTCCAACGAGATACTGGAAGAGGTCTTACGTCAAGCACAACAAGTGGGCCTGATGACCGACAAGCACAACTTCATAATAACTAATCTCGATTTGCACACAATCAACCTGACGCCGTTCAAATACAGCGAAACGAACATCACTGGGATGAGGTGTGTGGATCCAGACAAATTCTTAGCCGACGAAATGGATCCTCTAGCAGGATATCAACTGAAGTTGGAAGAAGCCCTCATTTATGATGCTGTTAAAATGTTCGCGGAAGCCATAAAATCAGTCGGTCGTATGGTACAACCTCTTTCTATAGACTGTTACAGTTACGAAGATAGACTGAAGTCTGGAACAACTATCATCAACTTCATGAGGAATCTGGAGTATCCAGGTCTGACTGGTCCAGTGAAATTTGACGTCAGAGGTTTCCGGACGGATTTTGGTTTGGATATATTCGAGCTCATGGAAGGGGGACAGACGATAGTCGGCAACTGGAATTCCACCAAAAGGCCCCACCTCAACGTATCGAGAGTCACTGTGAAAGGTGAAGATGTAAATGACGACATACGGAATAGAACATTCAAAGTTATGATAACTTTGACTGAACCCTATGGTATGAGAGTAGAATCGTTGGAACCTCTCTATGGAAATGACCAGTATGAAGGTTTCGCTGTTGATTTAATAAAGTTACTGGCGGAAATGAGAGGATTCAACTACACATTCGTTCTGAGGGAGGACAAAGCCAATGGAAAATTCGACAATAGTACCGGCAAGTGGACTGGGATTATCGGTGACCTTATCGATGGGAATGCTGACCTCGCAATATGTGATCTCACTATTACCATGGAAAGAGAAGCTGTGGTAGACTTCACGGTTCCATTCATGATGCTTGGTATCAGTATCCTCTACAAAAAACCTACCAAGGCACCCCCAAGCTTCTTTTCCTTCGCCGATCCATTCGCTTTCGAAGTTTGGGAACTACTAATGGTTGCTTGGATAGGAGTATCGTTGATCCTTTTTGTTGTGGGGAGGATTTCGCCTGGTGAATGGGAGAACCCTTATCCATGTATAGAAGAACCAGAATTTTTAGTCAACCAGTTGGATTTCAGGAACTGCCTTTGGTTTGTTACAGGATCCATCATGCAACAGGGATCTGAAATTGAGCTCAAGTCCTTTTCCACAAGAATGATAGCAGGTATGTGGTGGTTCTTCACGCTCCTGATGGTTTCTTCTTATACAGCAAATTTGGCGGCGTTCTTGACAACAGAAAATCCAGATCCACATTTCACCAATTTCAAAGAACTGGTGGAAAATGCTGAACGAAAAGGCATCAAGCTTGGCGCCAAGAGAATAGGAGCTACAGAGTCTTTTTTCGAAGACAAATGGAAAGCCGATCCCACTTCTGATTTTGGGAAAGCTTGGACTCTGATTCTGAAAGACAGGGACAAAATCAAGATACCTGATAACTCGGATGGTGTTTTCCACGCTCAGCAGGGATATTATGCATTTTTCATGGAGGACAAATCCATCGAGTATGAGACACAAAGAAAATGTGAATTGAATCAAGTAGGAGGAAAACTGGATGAGAAGGGCTACGGTATGGCTATGCGAAAAAATTCAACTTATAGGAATAGTTTGAGTACCGCTATTCTGAAATTGCAAAACTCCGGAAAAATAGACGAGATCAAGAGGAAATGGTGGGAAGAACGGAAGGGCGGAGGACAATGTTCGTCCGACGGAGAGAGTTCAGATGCTACACCCCTCAACCTCAAGGGAGTGGAGGGGGTGTTTTGGGTCACCATTGCCGGAACAATCATTGCCTTCCTCCTAGCCCTCTTGGAAGCCATCCTCCAAGTGACGAAAAAAAGCGATAAGGACGAAAACTTCCTTCGGCGCAGCACTCAAAGAAGAGATCAAATTTTATTTCAGATTCGGGGAGATGGAGAAACAAGTTTCTTA

>AquaIR 25a

ATGAGCATAAATGAGATATTTTTGATATTCCAGTTCGTTCTGATTGGATACTGCCATGGGCAAACAATCCAAAACATCAATGTGTTATTTGTTAATGAAGAAGGAAACGAAGTAGCAGAAAAAGCACTGGATGTCGCAATGACTTACCTAAAAAAGAATAATAAAATTGGGGTTGGTGTCGATGTCAGGAGAGTTGTTGGAAACCGAACAGATTCCAACGCATTTCTAGAATCATTATGTTCCACCTATGATTCAATGCTGGAGGCTCAAACGTACCCTCACTTGGTCCTGGATACGACCATGACAGGATTGGGATCAGAAACTGTGAAATCTTTCACGCAAGCTTTGGCTCTGCCAACGATAAGCGCTTCTTTCGGCCAAGAAGGGGATTTGAGACAATGGCGCAACATCGACGAGAACGAGAAGGATTTCTTAATCCAAATTTGCCCTCCTGCTGACATTATTCCTGAAATTGTAAGGACACTCGTTCTCAATCAGAATATTACTAATGCTGCAATTTTGTTCGATGAATCTTTTGTCATGGACCATAAATATAAGTCTCTCCTTCAAAACGTGGCCACACGGCATATCATAACAGCCATCAAACAAGGAAATCAGGTGGTGGAACAACTAAATCAGCTTCGTAAGTTAGATTTGGTCAACTTCTTCGTATTGGCGAGTCTGAAGAACATCAAGCGAGTTCTCGATGCCGCCGACTCCGTCAATTTCTTCAATAGGAAATTCGCGTGGCATGTCATAACTCAAGACGAAGGGGAAATGAAATGCGTCTGTCGCAACGCCACGATCATTTTCGTAAAACCATCCCCGAACGCCGCATTTCAAGACAGATTGGGGACTATGCAGAGGACGTACCAACTGAATATCGAGCCTATCATCTCTTCTGCCTTTTACTTCGATCTCACGTTGCGGTCCTTCATCGCAATCAAGGAAATGGTATCTGATGGCACTTGGAAAAATAGTGTTACAAATTACATAACTTGTGACGATTATGACGGAGAAAATAGTCCAAAGAGAGAGGGATTGAATTTGAAAAAATATTTCAATAAGGAAATTACAGAAACGCCCACCTACGGTCCCATAACTGTAGTCTCGAACGGCCTTAGCTACATGGAGTTTCAGATGCAATTGACCTCTGTGGGAGTAAGAGAAGGAGCATCAGACAAATCCACCATTTTAGGAACATGGTCTGCAGGTTTTGACAACAATTTGACGATCGTGGACCAACAAGTGATGGTGAATTTGACAGCTGACTTGGTTTACCGGGTTGTTACAGTTGAGCAAAAACCATTCATATTTCGAGACGAGTCGGCCCCCAGAGGATTCAGCGGTTATTGCATCGACCTCATCGACAAAATAGCGGACATCCTCCAGTTCGACTATGAAATCACAGCCGTTGATCATTTCGGTACCATGGACGAAAGTGGAAAATGGAATGGGGTCGTTAAGGAGCTCATGGAAAAGAGGGCTGATGTCGGACTGGGCTCCATGTCGGTCATGGCAGAAAGGGAAAATGTAATAGATTTCACCGTACCGTATTACGATCTGGTAGGGATAACGATACTGATGAAACTTCCCGAAACCCCTACAAGTTTGTTCAAATTTTTGACGGTTTTAGAAAACGAGGTATGGTTATGTATCCTGGCAGCGTACTTTTTCACAAGTTTTCTCATGTGGATATTCGATCGCTGGTCGCCATACAGCTACCAAAACAACCGAGATAAATACAAAGACGACGAAGAAAAGAGGGAATTCAACCTGAAAGAGTGCTTGTGGTTCTGCATGACCTCTCTCACGCCCCAAGGGGGAGGGGAGGCGCCCAAAAATTTGTCGGGCAGACTTGTGGCAGCCACCTGGTGGTTGTTCGGATTCATCATCATCGCATCCTACACTGCTAATTTGGCGGCTTTTCTGACTGTATCCAGACTGGACACCCCGATCGAGTCCTTGGACGATCTCTCCAAGCAGTATAAAATTCAGTATGCTCCGCTGAATGGCTCATCCACGCAAACTTACTTCGAGAGGATGGCGAATATCGAAGCGAGATTCTATGAAATATGGAAAGATATGAGCCTCAATGACAGCCTGTCAGAGGTGGAGCGAGCTAAACTAGCCGTATGGGACTACCCAGTCAGCGACAAATACACGAAAATGTGGCAAGCGATGAAAGAAGCCGGGCTGCCGAACACCATGGACGAGGCCCTGGAGAAAGTGAGATCGTCCAAATCGTCGAGCGAAGGTTTCGCCTTCCTAGGAGATGCCACAGATATAAAATATTTGGAGAAAACCAATTGCGATCTCATCGCGGTCGGAGAGGAGTTTTCCAGAAAACCGTACGCCATCGCTGTTCAGCAAGGGTCTCCTCTCAAGGACCAGTTCAATACAGCAATTCTGCAATTACTCAACCGACGTGAGTTGGAACGACTCAAAGAAAAATGGTGGAATAGAAATCCAGAGAAGAAGGATTGCGACACAGCCGACGACCAGTCAGATGGAATTAGCATCCAGAACATTGGCGGGGTGTTCATTGTAATTTTTGTTGGTATCGGATTGGCTTGCATCACCCTGGCTTTCGAGTACTGGTGGTACAAATACAGGAAGGTATCCAGAATCATCGACGTTCAAGGAGCAGCAAATCAGCCGGGTAGGCCTACCTTGAAGAAGAACCAGGTTATTTCAAAGCTCAAAACATCGGAGGAAGCCACTTCCAAAAAGAGAGAACGATTTTTTCCCAGATCAAGATTT

>AquaIR 41a

ATGTTTTCCCTGTATTTTCCAACAAAAAGTGAACTGTTCCCTTCGCTGCTCACCACAAATGCGTCATTGGCTATCGTTATTGATCGTGAATATCTTGAAGATGAATACGATACAGTAAAGACAGATATAGAGGAGTACCTTCTTTATGCCAAAAGAGAAATATTGAAACATGGTGGCGTTAACGTACACTTTTATTCCTGGACAGCAATGAATGTCAGAAAAGATTTGGCGGCGATTTTCAGTATCGCATCTTGTTCGGATACTTGGAGACTTTTCCATTCAACTGATGGTGAAGAATTGATCCATATGGCAATTACAGAATCTGACTGTCCCAGACTACCCACCGATGCTGCTCTAACAGTTCCATTGATAGCTAGAGGACAAGAATTACCACAGATTCTACTGGATCTTCGAATTGCAGGAGTATACAACTGGAAATCTGTGGTGATAATATACGATGCAACTTTGGACAGAGATATGACAACGCGCATCATCAAATCGGTTACTCAAATGTCCAATAATGACGGTGTCAAGGCAACGGGAATATCTCTGATAAAGCTCAAGAAGAATATATCTAAAAGTAATCTCAAAAAAATCCTTTCGGCTATAGATTCCAAAACTGTTGGAGGCAACTTCCTGGTTATAGCAAGTTACTATCTAGTTGGAACCATTATGGAATATTCAAAGTCCTTGAAACTTGTCGACACCAGAAACCAATGGTTGTACGTTATTCCTGATACCGATCGAAGATACCACGATATGCATGTGTTCAAGGATTTGTTGAAAGAGGGTGACAACGTTGCTTTTATCTACAATACTACGGTGACATCCAATAATTGCGTAGGCGGAAGAAAGTGTCAAATTGAAGAAATCATAAAAGCATTCACGAGGGCTTTGGACGAAGCGATCCAGGATGAATTTGAGACTGCGAGCCAAATCGCGGAAGAAGAATGGGAAGCTATCAGGCCGACCAAGATAGAAAGAAGAGATTTCTTGTTGAACAGGGCCAAAAAATTTGTTTCTAAAAACGGCGTCTGTGATAACTGCACATTTTGGGAGATGGAAACAGGTGAAACCTGGGGAAAAGAATACCAAAGTCTCGAAAAAAATGTCACGGCAGCATTGGTTCCCGTAGGAACATGGAGACCGAGTGATGGGACCACAATGACTGATGAATTATTTCTGCATATAGCTCATGGTTTCAGGGGAAAACTATTACCAATGGTTACCTTTCACAATCCTCCATGGCAAATACTGAAGTTGAGCGAATCGGGAAATGTCATAGAACACAAGGGATTGGTTTTCGACATCATCAGAGAGCTGGCCAAAAACCTCAACTTCACTTTCAGACTTGAAGTGGTGAACAAAACCTCATTTTCTGCAAACGCGACTTCTTTAAGCTCTTCGTACAATATCGTCGGAAACTCTTTGACGAACCGTATTCCCGCTACCATTCTGAATATGACGAAAAATAAATTTGTTGCTATGGGAGCCTGTGCCGTCACAGTTACTGACGAATTCAAGTATATCATCAATTTCAGCAGACCGATCAGCACGCAGACGTATACGTTCCTGGTTGCGAGACCTAGAGAGCTGAGTAGGGCTTTGCTATTCATCTCGCCTTTCACCGGAGATACATGGTTATGTTTGGCAGCATCCATAGTCTGTATGGGTCCGATTTTGTACTATATTCACCGATTTAGCCCCGTTAAC

>AquaIR 64a

ATGCAAACCACGGATCCGGATGCCATAAACTTGTACGAAAAGAAAATCAGAGGTTCTACGAATTCCTCTGGATTCTACTCGCCCTTCGAAGGTCTAGATCTGGTGAGGAGGGGTGGTTTTGCTTTCCATGTCGAAACGAGTACAGCCTATCCGATTATAGAGGCAACATTTTCTAATCAAGAAATTTGTGAACTTGATGAAATTCAGATGTACAGGACACAACCAATGCATACAAACTTGCAGAAGGGTTCTCCTTTCAGAGAAATGATGAATTTTTGTATGCTCAAGCTGGTTGAAAATGGCAATATGGATCGTTTGAGAAAACACTGGGACGCGAGGAAACCAACATGCATCGAGTCAGCCAAAAAACAAGAGATACACGTTAGCCTCAGCGAATTCTCCTGTTCCACTTCAGCTCTGATTATTGGCATATGCATTTCTTTGATATTCTTAATTAGTGAATTTGTTACACATTTCAGAATTTCTCTAGTGAATCTTCTAAAGCCACCTTTGGAAATCAGAGGGCATCCCGATCAGGTCTATCCCTTTGTAGAG

>AquaIR 75q

ATGAGAAAACATTGGGAATACAGCGATGAGTTTAACAATGCCCTCTTGGAGTTACTCGAGCAGGGAAGATTAAGTGAATTAAAGGATAAATGGTGGAAACAAGTTGGAGGTGGTGTATGTGCGAGTAAACCAGAACAAAGCGATCCGAAACCCTTAACCATGGAAAATCTCGGCGGCATTTATATAGTACTGGTGGTAGGCAGCGGTATGGCACTTATACATGCCATTATCAGTTGGTTGTGTTTCATTTTTCGAAAAGCTCGAAGTCATAAGGTACCTTTGAAAGTTGCGTTTAAAGAAGAATTAAAATTCGTACTGGAATTTACTCTGTATACCAGAGACTTGAAGGCGGCCGCTTCGATTTACTCACCTAGTCGAAAGCCA

>SNMP 1

ATGAAGTTTCGAGTGAAGTTGGCGATAGGATCTTTCAGTGCTTTGATTTTCATCATTTTGGTGGGTTTTGTGATTTTCCCCAGACTGCTCACATCGAAAGTGAAAGGGATGGTCCACCTAGGTCCAGGCAGCGATATAAGAGAATTGTTCATCAAAATACCATTTGGTCTATCATTCAAGGTTTACCTGTTCAACGTTACGAACCCAATGGAAGTACAGAATGGAAGCAAACCCATCATCAAAGAGGTTGGACCATTTTGCTACGAGGAATGGAAAGAAAAGATGAATGTGAAGGATTCGGATGCAGACGATACCATAGCGTACAATCAAAAAGACACATTTGTGAAACAAAGATGGCCTGGTTGCAGGACTGGCCAAGAAGAAATCACGATACCACACCCGATGATACTGGGTCTTGTTAACACTGTGTTCAGAGAGAAACCAGGAGCTCTGTCATTGGTCAACAAAGCATTGAAGACTATATACGGCAATCCTTCTTCCATTTTCGTTACCGCCAAAGCAGATGACATCTTATTCGACGGGATCATCATCAACTGCGGTGTCACTGATTTTGCCGGCAAAGCGATTTGCAGTCAACTGAAGGGTGCAGAAATGTTGAAGAAAATCAGTGAAGACGAACTGATGTTTTCACTACTGGGACCGATGAATGCCACACTGCAAAAACAAATCAAGGCTTTCAGAGGCACGAAAAACTACCGTGACGTAGGAAGGATTGCAGAGTACGACTACGCTAAAACAATGAAAGTGTGGCCAACTGAAGAATGTAACAAAATCAGAGGAACAGATGGTACCATTTTTCCACCTTTGATGGGATTTGAAGATGGGCTAGCTTCGTTTTCGCCAAGTTTATGCAGGTCACTTTTGGCATTTTTCGTACGGAAGAGGACATATGACGGTATCCCATGTGGAGAATACACAGCCAATCTGGGGGACATGTCCAAGAATGAGGAGGAGAAATGTTACTGTACTACGCCTGGTACTTGCCTCAAAAAGGGACTGATGGACCTGTATAAATGTTCTGGAGCGCCTATTTATGTTTCGTTACCTCATTTCTACGACTGTGATGACAGTTATCTCAAGGGGGTGGTGGGGCTAATGCCTAACAAGACGAAGCATGAAATAAGAATATTGTTTGAGACGACAACTGGCAGTCCTGTATCAGCAAGAAAGCGTCTCCAATTCAATATGCCGATAGAGCCCATTCATAAAGTGGATTTGTTCAAGAATTTCACGCCAACAATACTGCCAGTCTTCTGGGTAGAAGAAGGTGTCGATCTTAATAGAACGTACACGGGCCAACTGAAGAGTCTGTTCACACTGAAGAAAGTCGTGAAAGTGAGCAAGTGGATCATCCTCGTCGGTTCTCTGGGAGGTTTGGCAACATCGGGATATCTCTTCTTCAAAAGCAATGGAACGGCCGATCAAACCGCTGTTCATGAAATCAAAAGAAACGAAAATAAGAGTGCAATATCCATGGTCAATCATCACTTAGGGGGTCATGTGAATGATGGAATGTCAGGGGATGAACCGGATAAATAT

>SNMP 2

ATGATCAACGGAAGCAAATTCTGCACGGTGAAAGTTCTTCTAGTGACTACAGTGGTGCTACTAATGATTTTACTCGGAGTGTCAGTGCTCAGTTTCGTTGGGATGCCTCTAATCATTGACGACCAGATTGCACACCAATTACGTTTGGAAAATAACACGGAACAGTGGGATAGATTCAAAGAATTGCCCGTTCCCTTGAAGCTGAAGGTATTTGTGTTCAATGTGACGAATTCGGAAGAGGTGATGACAGGAGCAACCCCAGTTGTGAAGGAAGTGGGACCTTACTGTTATCAAGAGAAGATCAGAAGAAACATCTTGTCTGCCAATTCTGAAGAAGATAGTGTAACATATGAGCAAACATTCAATATATCTTTCGATGAAACCGGCTCAGGAGACCTTAAAGACACAGATACTGTTATTATTGTGAATCCATTGTTATTGATCTTGTCCCAAATAACAAATGTGGTTGAAAGATTTGTAATATTAGGATGCCTCGACAAAGTGATGCCACCCGAATATAACACATTGTTCATCAAAATCAACATAAAAACAGCAATGTTCGATGGTCTCGAATTTGCGGTGGCTTCAGATGATATTGGGCCTGCTTGCAATATTGTGCGTACTAAAATTCTGGAAAAAACTAAACCCATGAAGAATGTCGAAAGGATAACGAGCCCAAGCGACCCCACTAAGATTACGTCGTTGAAATTCGCATTTCTTCAATACAAAATTAGAGAACCTGATGGAGTTTACACGACGAACAGAGGAATAAACGATATAACAGAATTGGGACACATCATGAGATGGAATTATAATTCAGAATTGCCCTACTGGGGACGTTCACAATCCATCAACAACGAAACGTGCAAGAGAGTCAGAGGTACAGATTCGACTATATACCCACCCCATGTTACGAAAAACAGCGTTTTCGATATTTTTGCAACAGATATATGCAGAACTGTGCAAATAAAATACAAAGGAACTGGCAGTTACGGAGGAATCGACGGATATCGTTTTGGAGTTGACGATAATACTCTGCAACCAGCCACTCCTAACCCAGATAATGATTGTTATTGTGTTCAACAAACACAGGGTGTCAAAGGGGAACCAACATGTTGGATGGATGGCATCATAGACGTCTTTCCATGTTTTGGTGCTCCAGTCTTACTATCGTTTCCCCATTTCCTATATGCGAATGAGTCATACCTTGATGGCGTCGAGGGAGTGTCTAAGCCCGATTCTTCAATTCATGAACTATTTTTGTTGATCGAACCGCATACTGGAGTTCCGTTACAAGGCATGAAAAGGATACAGCTGAACGTAGTTCTGAGGCCTGTTGCCTACATACCATATACTGAAGGTTTGAAACCTACTCTCCTGCCACTAGTTTGGATCGAAGAGGGTGTCAACCTATCTGAAGAACTGATCGACAAGCTAAACAGTATGTATTTCGATGTATTGAAAATTGCTGATGGTGTCAAATATGGACTTATAGCTGTGACTCTGGCATCTTTATTGATTTCTAGCGGATTTCTCTTGAGAAAAAAATATTTT
