# Supplementary material for: Identification and Comparison of Candidate Olfactory Genes in the Olfactory and Non-Olfactory Organs of Elm Pest Ambrostoma quadriimpressum (Coleoptera: Chrysomelidae) Based on Transcriptome Analysis
Source: PLoS One. 2016 Jan 22;11(1):e0147144. doi: 10.1371/journal.pone.0147144 (PMC4723088; doi:10.1371/journal.pone.0147144)
Supplement: S2 Table — (DOC) [file pone.0147144.s002.doc]

**Supplementary material 2**

**Primer used in RT-qPCRs**

| **Primer name** | **Primer sequence** |
| --- | --- |
| AquaOBP 1 Forward Primer | ATATTCTACTAGCAGCCGGTGCATCATC |
| Reverse primer | CCACATCCATTCTAAAACCTTCTACATCTTCG |
| AquaOBP C1 Forward Primer | TGAGGGAAAGTGCCAGAAACCAGATG |
| Reverse primer | GGATCGTCGCTTACTTCACCATTATGC |
| AquaOBP 2 Forward Primer | GGAGGAGAGAGTCCACCACCAGTATTC |
| Reverse primer | AAGCAGAGCATAGGTTCTTCCAACTCAG |
| AquaOBPC2 Forward Primer | GCATGAACGTCCACGCAGGTCTG |
| Reverse primer | ACCATCTCGTACTCCGCAGTCCTTC |
| AquaOBP3 Forward Primer | CTGAGGAACTCGATGCCATGCTTACTG |
| Reverse primer | CGTTGATTCCCGCTTCCCAGTCG |
| AquaOBPC3 Forward Primer | GCCAATCCGATCCAGCCACAGTAG |
| Reverse primer | CCCGAGTTTGACGTTCATGCAGAGAG |
| AquaOBP4 Reverse primer | CAAAGCGGATTCATAGTTGAGGGTGTTATC |
| Forward Primer | GAAAGCCACAGATGCGGACATTGAC |
| AquaOBP C4 Forward Primer | CTTCGTCGCAGTAGGTCTTGGGATC |
| Reverse primer | GCTTGAAGATGAAGGCTGTGTTGGTTC |
| AquaOBP 5 Forward Primer | TTCCTGCATTGCCTCTTCACCAAGTAC |
|  | AACGACACTTTGATGACGTGGAGAAGG |
| AquaOBP C5 Forward Primer | TGATGAACCCTGATTACACCCTGAATGAAG |
| Reverse primer | TCGTGTGGCTCAGCAATACCAGATTC |
| AquaOBP 6 Forward Primer | GCGTCTGATTACATGGATGCCGATGATC |
| Reverse primer | TCCCACCTCTTCTGCCCCTTTGATAC |
| AquaOBP C6 Forward Primer | AGTGATGAACAGCAATCTGGAGGTGAATAG |
| Reverse primer | AGACAAGTGCTCTCCATATCATCGAAGTTATC |
| AquaOBP 7 Forward Primer | TCCTCTTCGGTTGGAGTCAGAATCAATAATG |
| Reverse primer | CGCCTCTATGTCAGCCATCGTAGC |
| AquaOBP C7 Forward Primer | CGCCTTCAATAGACCCATACGCAGAC |
|  | TCATCGTCTCTTGGGAAGCTCAGTGTAG |
| AquaOBP 8 Forward Primer | ATACACTTTCCCAGACCATAAGCACACC |
| Reverse primer | TGTTGGCTTCGTTGAAACCCTTCCTTAC |
| AquaCSP 1 Forward Primer | TCCATATTTCTCGGCGTGTGTGCTATTG |
| Reverse primer | GCGTCGTCGGAAATACTTGGTCTATCTG |
| AquaCSP 3 Forward Primer | GCGTCCTAGTGTCCTTCATGCTATGC |
| Reverse primer | TTCCAGCACACACATAATCTGACGACTC |
| AquaCSP 4 Forward Primer | CAACATCGATTATGAGCAAATTCTACAAAGCG |
| Reverse primer | AGTTCCTTTCCGTCAGGTGAGCAAG |
| AquaCSP_5 Forward Primer | CCGAATGGTGGAAGGAACTCTGTGAC |
| Reverse primer | CAGCGAGAGCTTGTTGGATGAATGAATC |
| AquaCSP_6 Forward Primer | ATGCACCGACATCCAAAGGAAGAACTC |
| Reverse primer | TGTCTGGCTCTGTAGGCACCATTAGG |
| AquaCSP_7 Forward Primer | TGTTTGGGTTAGCGGTGGCAGTTC |
| Reverse primer | GTAGTAGTTGACCATCCTCCGGTTGTTG |
| AquaCSP 8 Forward Primer | CCACAGCAGTCTTGTCAGCAGTACC |
| Reverse primer | TACACTTTCCTTTCTCCAAGAGACAGTTCAC |
| AquaCSP 9 Forward Primer | CGATGCTCTGAAGAACGGATGTGATAAATG |
| Reverse primer | GTTTCTTGTAGTGTCCTTGAGGGTCATATTTC |
| AquaCSP_10 Forward Primer | TCGTCATAGTTACCGTTGCCCTTTGTG |
| Reverse primer | AACTCGCTAGGACCCGCTTATTTGC |
| AquaOrco Forward Primer | TAATACGACTCACTATAGGGCTGACCTTATGCCCAAC |
| Reverse primer | TAATACGACTCACTATAGGGCAACCCATATTTCTCAGG |
| AquaOR_6 Forward Primer | CGATAGCGGCTTGGGTTCCATTTC |
| Reverse primer | GCAATCAGGCAGTCAGCACTCAAATC |
| AquaOR_8 Forward Primer | GCGGTAGTTGTCCCTGCTGTTACG |
| Reverse primer | TTCCAGTATTGATAGCGACGAGTCCTTC |
| AquaOR 9 Forward Primer | GTTCGTATTCAGAGTGTTGGGCTTTTGG |
| Reverse primer | GTAGCTTGGGATGACAGGAACAGAAACC |
| AquaOR 10 Forward Primer | TGACTGGTCAAAGCCGTTCAACTATGG |
| Reverse primer | AAGTCGCACTGCAAGCCAACCTG |
| AquaOR 14 Forward Primer | ACACGTTCAGCCTACCAGTTTGAAGTTC |
| Reverse primer | TCCACACCAGCAGTACATTACGAGTTG |
| AquaOR_15 Forward Primer | CTTCACCGCACCAACACCAATTCTTG |
| Reverse primer | CCATAGCTTCACCAGCAGAATGCCTAG |
| AquaOR_17 Forward Primer | GCTCATTTCGTCTGTGGCAACTTTCTG |
| Reverse primer | ACTCCCTTCATCAATGGACATTCTTCTG |
| AquaOR_18 Forward Primer | CATCATCACTTTCTTCACGCTCACCTTC |
| Reverse primer | TGTGTCCGAGAATATCCAGCAGCATTG |
| AquaOR_20 Forward Primer | CCAGTCTATTCTTCGGGTGGATGTTCTC |
| Reverse primer | ACCAACAGCAACACTCGCTTCTGATAG |
| AquaOR_22 Forward Primer | TGTACGTCCTGTCCCTGCCTCATAC |
| Reverse primer | GCCACTTCCTCAGCTTCATCCATCAATC |
| AquaOR_23 Forward Primer | CACAAACAAGGGTCGTACCGGAGATAAC |
| Reverse primer | CGGCAAAGAACTGGAGGCTGAATAAAAC |
| AquaOR 24 Forward Primer | ACCTTCTCCAACGTCCTCCTAGCATATC |
| Reverse primer | AATGTCGCCGCAATGAGATAAGTGAGAG |
| AquaOR_26 Forward Primer | CGAAACCAGCACCATCTATACCGAAGTC |
| Reverse primer | TGTCCGTTGTAAGTAGAGGCCCTATGAG |
| AquaOR 29 Forward Primer | ATTGCCGAAATGGTTGGAACTCATCAC |
| Reverse primer | TTCTGACGCCACATTCATGTTCAATACG |
| AquaOR_31 Forward Primer | CGACTTGAGCCCTGAGAGCAACTTTAG |
| Reverse primer | CGACTCATCTGTGATTCTCTGGCAACTC |
| AquaOR 32 Forward Primer | CGTTCAACTCAAGTGCTGCGTCAAAG |
| Reverse primer | ATAGGGCTCGTCCTCTCTGTAATGTTCC |
| AquaOR_33 Forward Primer | GCTGGTGCTTACAATGTGCCTTTCC |
| Reverse primer | TTAACCCATTTCCGAACTGTCCGTATCC |
| AquaIR_4 Forward Primer | TTGGCTAGAGTTGGCGGGTTGTTG |
| Reverse primer | GCGGTGCTCAGATCGTTCCTATATGG |
| AquaIR_5 Forward Primer | AAGGAAGTGGTGGGAAGAGAGAAATGTC |
| Reverse primer | ACCGAGCAGACCAACGCCAATAAC |
| AquaIR 8a Forward Primer | AGGACGGTGTTTGGGATGGTGTTATAGG |
| Reverse primer | GGGCGACGAAATCAACGACTTCTTCC |
| AquaIR_11 Forward Primer | GTTGTCGTTCAGCCATCCCCTATCATTC |
| Reverse primer | TTCAACCTTGCCACCAGGAACATCATC |
| AquaIR 13 Forward Primer | CGAAGGAGAGCCGTAGACAAACAAAAG |
| Reverse primerr | GTGGAACATCAATTCTGCTACCAGGATC |
| AquaIR 14 Forward Primer | TGCCAACATACGAAGAAGGTATCCAGAG |
| Reverse primer | CCTTAGAATCCAGCAGCCCTCCTATTTG |
| AquaIR_16 Forward Primer | GAACGGAAGGGCGGAGGACAATG |
| Reverse primer | CAAGAGGGCTAGGAGGAAGGCAATG |
| AquaIR 25a Forward Primer | ACGATACTGATGAAACTTCCCGAAACCC |
| Reverse primer | GTACGCTGCCAGGATACATAACCATACC |
| AquaIR 64a Forward Primer | CCTCAGCGAATTCTCCTGTTCCACTTC |
| Reverse primer | GATCGGGATGCCCTCTGATTTCCAAAG |
| AquaSNMP 1 Forward Primer | TTCACGCCAACAATACTGCCAGTCTTC |
| Reverse primer | AGAGAACCGACGAGGATGATCCACTTG |
| AquaSNMP 2 Forward Primer | CTCGGAGTGTCAGTGCTCAGTTTCG |
| Reverse primer | GCTTCAAGGGAACGGGCAATTCTTTG |
| Actin Forward Primer | ACCTCTTTTGCTTTGGGCTTCATCTCC |
| Reverse primer | CATCGTCGGTCGTCCAAGACATCAAG |

**Primer used in RT-PCRs**

| **Primer name** | **Primer sequence** |
| --- | --- |
| AquaOBP 1 Forward Primer | TTCGTGTTGTCAATACTAGC |
| Reverse primer | CTCTTCATATCCTCACATTCC |
| AquaOBP C1 Forward Primer | TCTGCTTTGGTGATTATTGTTG |
| Reverse primer | ATACTTTGGAAGGCGGATTC |
| AquaOBP 2 Forward Primer | AGAGTCCACCACCAGTATTC |
| Reverse primer | ATCGTCATCATCAAGCAGTC |
| AquaOBPC2 Forward Primer | TTGTTCGGTTCGTTTATCTG |
| Reverse primer | AATAGACGCTTCTTCAGGAG |
| AquaOBP3 Forward Primer | GAAATACTTGCCAGAACAAATC |
| Reverse primer | GTAAGAAGTAATTGTCAGGATTATC |
| AquaOBPC3 Forward Primer | CGCAATTACTTTGGTTTGTG |
| Reverse primer | CATAACACTCATATAAAGCAACTG |
| AquaOBP4 Forward Primer | TATTCTCAATGTTTCTGGGATG |
| Reverse primer | GCTAATTCATATGCTGCAATAC |
| AquaOBP C4 Forward Primer | GTAACGAAACACAACCAAATG |
| Reverse primer | TACTATGGCTCATCACCTTC |
| AquaOBP 5 Forward Primer | ATGGTGAACTGCGATATAGC |
| Reverse primer | CTTCTTCAGATTATTCTTACTATGC |
| AquaOBP C5 Forward Primer | CTTGGCAGACACGGATTATG |
| Reverse primer | GGCTCAGCAATACCAGATTC |
| AquaOBP 6 Forward Primer | AACTGATTGGCGAATTGATG |
| Reverse primer | TCATCGGCATCCATGTAATC |
| AquaOBP C6 Forward Primer | TCTGGAACGGAAGGATTATG |
| Reverse primer | ATTTCGTAGTGCTCTTCATTG |
| AquaOBP 7 Forward Primer | GTTCTCATTCATTTGGTTCTTC |
| Reverse primer | CTCACATACTTCCGATAACTC |
| AquaOBP C7 Forward Primer | AAGAACTGTCTATTTAGTGTTATTG |
| Reverse primer | ATCGCTCCTTTTGTAAAACC |
| AquaOBP 8 Forward Primer | AATAGTGTACCAGATGAAATATACG |
| Reverse primer | ATCCTTACCATCGCCTAGAC |
| AquaCSP 1 Forward Primer | TGGAAATCAAATATCAGAACTTTATC |
| Reverse primer | GCATATATCCCAGGACCTTC |
| AquaCSP 3 Forward Primer | ATGAAACCAATTATGAGACG |
| Reverse primer | ATATTTCCTCAAGAGCATGG |
| AquaCSP 4 Forward Primer | CATCTGCTCCTTACTCCTAG |
| Reverse primer | GAACTTCTGCTTGAAACTATG |
| AquaCSP_5 Forward Primer | TGTTGTGGTAGTTGTGCTAG |
| Reverse primer | AATGGTCACAGAGTTCCTTC |
| AquaCSP_6 Forward Primer | CCACCTTAGCCTGTTTATTG |
| Reverse primer | ACCATTAGGATCGTATTTGTC |
| AquaCSP_7 Forward Primer | AAGACAATAGCATAGTGATGAAG |
| Reverse primer | GTCTCCTATGGCACCAATAC |
| AquaCSP 8 Forward Primer | TTTGATGATATTATCCTGTTTGAC |
| Reverse primer | TACCTCTTCTTGTAGCTTCC |
| AquaCSP 9 Forward Primer | AAGTGTGAGTGCATTGATTC |
| Reverse primer | TTGATAGCGTTTCTTGTAGTG |
| AquaCSP_10 Forward Primer | TGTTTGTTCGTCATAGTTACC |
| Reverse primer | ATTTAGGAATTGTCTGAAACTTG |
| AquaOrco Forward Primer | TACAATACAAGGCAAGAGATG |
| Reverse primer | CTAAGTATCCTATCACAGTAGC |
| AquaOR_6 Forward Primer | AAACTAGCGGAACTGATAAAC |
| Reverse primer | GAGCACAGATAACCATGATG |
| AquaOR_8 Forward Primer | TTCTAAGATGTTCACAGGTTAC |
| Reverse primer | AGTATTGATAGCGACGAGTC |
| AquaOR 9 Forward Primer | AGTTGACAGTGGTACGAATC |
| Reverse primer | TAGAGCGAAGCCAGAATATG |
| AquaOR 10 Forward Primer | ATCTGGCAAGGAATGTGAAG |
| Reverse primer | ATTCTCTATATTACTCAATGTTATACTC |
| AquaOR 14 Forward Primer | CTCACCACATCAGTAAATAATTC |
| Reverse primer | CAGTTAAGAAGAACACCATTATC |
| AquaOR_15 Forward Primer | TCACTACGACGCAATTTATG |
| Reverse primer | TACCAAACATTTCTTAGATTTCTG |
| AquaOR_17 Forward Primer | CGAAATTGCTGCCATTATTG |
| Reverse primer | TCATCATTAGTAGAAGAATCATATTAG |
| AquaOR_18 Forward Primer | ATGAGGAATGTAATAGATGAGATG |
| Reverse primer | AAGATTATGATAGCAGTGAAGAG |
| AquaOR_20 Forward Primer | ATACTGCTCCTTTGTTATCTATC |
| Reverse primer | CGAAGAATAGACTGGTAGAATC |
| AquaOR_22 Forward Primer | AGTGGTTGATATTATTCGTTGTG |
| Reverse primer | TTCGCTTCTCCTGTTGATTC |
| AquaOR_23 Forward Primer | CATCTATTCCTGACTATGACTAC |
| Reverse primer | TGGTAATCGCAATACTTCTTG |
| AquaOR 24 Forward Primer | CCACCACAACTTTCTGATAG |
| Reverse primer | TACCAGCCATTATTGATACTTC |
| AquaOR_26 Forward Primer | TTCATCATCATACCTTCTTACTAC |
| Reverse primer | ACCATATTCTGTCCTCTAAGC |
| AquaOR 29 Forward Primer | GGGAACAGTATTTGAATATAAGAAG |
| Reverse primer | TTCGGTAGCAATCAAGAATATC |
| AquaOR_31 Forward Primer | TATTCGTTTCCTCAACATATTTC |
| Reverse primer | TATAACATACTCTCCACATCTTG |
| AquaOR 32 Forward Primer | AAGTAAATGTTGGGATTCTGAG |
| Reverse primer | CATAGCAAGAGAGTGTCAAAG |
| AquaOR_33 Forward Primer | GTGGAAGTTGAATCAGAGAC |
| Reverse primer | TATGCCGTAGATACCATCAG |
| AquaIR_4 Forward Primer | GATGTACGATCTCACGATTG |
| Reverse primer | GTTCTGTTGAAGAGGTTGTC |
| AquaIR_5 Forward Primer | AGGATGAGTTCACTGTCTTC |
| Reverse primer | CGCCTTCTTCTGTTAATGAG |
| AquaIR 8a Forward Primer | ATAATGAGCAATCCAGTGAAC |
| Reverse primer | ACTTCGTTACCAATTCCAATG |
| AquaIR_11 Forward Primer | TGATGGGTGAGATTTGTAAAC |
| Reverse primer | AATACGAAGTAGCAATGTAGAC |
| AquaIR 13 Forward Primer | GGGAAGCAGAAAGAATTGTC |
| Reverse primerr | ACGAACGTAGTTGATTGATATG |
| AquaIR 14 Forward Primer | ATGCCAACATACGAAGAAGG |
| Reverse primer | CCGATACTATCCACTCCTAATG |
| AquaIR_16 Forward Primer | TAGACTTCACGGTTCCATTC |
| Reverse primer | CACATACCTGCTATCATTCTTG |
| AquaIR 25a Forward Primer | TATTGGCGAGTCTGAAGAAC |
| Reverse primer | TCCGTCATAATCGTCACAAG |
| AquaIR 64a Forward Primer | TACAGCCTATCCGATTATAGAG |
| Reverse primer | TCACTAATTAAGAATATCAAAGAAATG |
| AquaSNMP 1 Forward Primer | AATCAGAGGAACAGATGGTAC |
| Reverse primer | CTTCGTCTTGTTAGGCATTAG |
| AquaSNMP 2 Forward Primer | GGATGCCTCTAATCATTGAC |
| Reverse primer | TGGATTCACAATAATAACAGTATC |
